# Vanadium Oxide: Phase Diagrams, Structures, Synthesis, and Applications

**DOI:** 10.1021/acs.chemrev.2c00546

**Published:** 2023-03-27

**Authors:** Peng Hu, Ping Hu, Tuan Duc Vu, Ming Li, Shancheng Wang, Yujie Ke, Xianting Zeng, Liqiang Mai, Yi Long

**Affiliations:** ‡ School of Physics, Northwest University, Xi’an 710069, P. R. China; † School of Materials Science and Engineering, Nanyang Technological University, 50 Nanyang Avenue, Singapore 639798, Singapore; ¶ State Key Laboratory of Advanced Technology for Materials Synthesis and Processing, Wuhan University of Technology, Wuhan 430070, Hubei, China; ⊥ Foshan Xianhu Laboratory of the Advanced Energy Science and Technology Guangdong Laboratory, Xianhu Hydrogen Valley, Foshan 528200, Guangdong, China; ∥ Key Laboratory of Materials Physics, Anhui Key Laboratory of Nanomaterials and Nanotechnology, Institute of Solid State Physics, Chinese Academy of Science, Hefei 230031, P. R. China; ∇ Institute of Materials Research and Engineering (IMRE), Agency for Science, Technology and Research (A*STAR), 2 Fusionopolis Way, Innovis #08-03, Singapore 138634, Republic of Singapore; § Singapore Institute of Manufacturing Technology (SIMTech), 2 Fusionopolis Way, #08-04 Innovis, Singapore 138634, Singapore; ○ Department of Electronic Engineering, The Chinese University of Hong Kong, Shatin, New Territories, Hong Kong, SAR 999077, China

## Abstract

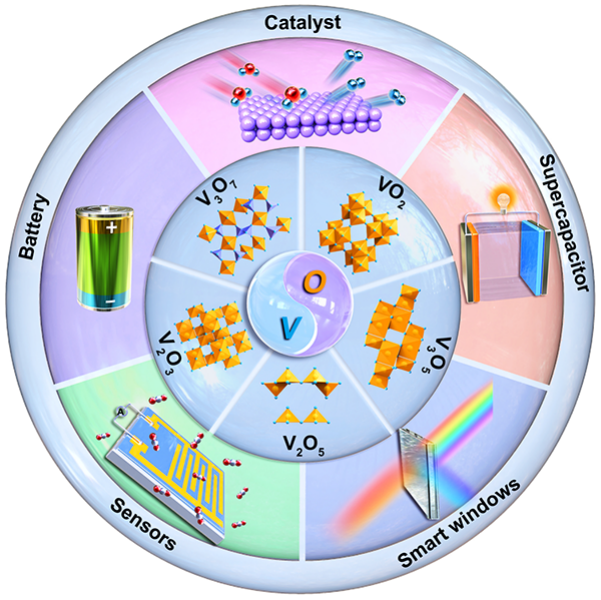

Vanadium oxides with multioxidation states and various crystalline structures offer unique electrical, optical, optoelectronic and magnetic properties, which could be manipulated for various applications. For the past 30 years, significant efforts have been made to study the fundamental science and explore the potential for vanadium oxide materials in ion batteries, water splitting, smart windows, supercapacitors, sensors, and so on. This review focuses on the most recent progress in synthesis methods and applications of some thermodynamically stable and metastable vanadium oxides, including but not limited to V_2_O_3_, V_3_O_5_, VO_2_, V_3_O_7_, V_2_O_5_, V_2_O_2_, V_6_O_13_, and V_4_O_9_. We begin with a tutorial on the phase diagram of the V–O system. The second part is a detailed review covering the crystal structure, the synthesis protocols, and the applications of each vanadium oxide, especially in batteries, catalysts, smart windows, and supercapacitors. We conclude with a brief perspective on how material and device improvements can address current deficiencies. This comprehensive review could accelerate the development of novel vanadium oxide structures in related applications.

## 1 Introduction

### 1.1 Vanadium

Vanadium was first discovered by Andrés Manuel del Rio in Mexico City from Pb_5_(VO_4_)_3_Cl in 1801.^1^ However, it was wrongly identified as a form of chromium by Hippolyte Victor Collet-Descotils in 1805.^2^ Until 1831, Swedish chemist Nil Gabriel Self-ström in Stockholm named the element vanadium, which is from the Norse Goddess Vanadis and means beauty and fertility.^3^ In the Earth’s crust, vanadium is the 20th most abundant element and the sixth most abundant element among the transition metals.^3−5^ However, some literature indicates that vanadium is the fourth most abundant transition metal after iron, titanium, and manganese.^5−8^ High purity vanadium (about 99.7%) was first produced in 1925 by reducing vanadium pentoxide (V_2_O_5_) with calcium metal.^1^ Pure vanadium exhibits a transition metal feature, which shows a high melting point and good corrosion resistance at low temperatures. Vanadium can be dissolved in nitric and sulfuric acids but is insoluble in hydrochloric acid.^9^ In nature, vanadium is difficult to exist in metal form because it easily reacts with oxygen, even nitrogen and carbon at elevated temperatures.^2,10^ Vanadium is an important component of specific steel alloys, which provides additional tensile strength and extra protection against rust and corrosion of these materials.

### 1.2 Vanadium Oxides

Vanadium has the electronic configuration [Ar]4s^2^3d^3^. Therefore, the oxidation state of vanadium can range from +5 to −3, and the valences of +5, +4, +3, and +2 are most commonly observed.^11,12^ Four vanadium oxides feature single oxidation states (+2 for VO, +3 for V_2_O_3_, +4 for VO_2_, and +5 for V_2_O_5_), and others have mixed oxidation states. Different oxidation states exhibit various colors: +5 (orange to yellow), +4 (blue), and +3 (green).^9^ The vanadium oxides exhibit crystalline structures with different oxygen coordinations, which result in the formation of octahedral, pentagonal bipyramids, square pyramids, and tetrahedral sharing corners, edges, or faces.^12^ The oxidation state of the vanadium cations dramatically affects the physicochemical properties of the vanadium oxides with different phases.

Due to the multioxidation states and various crystalline structures, the vanadium oxides exhibit excellent intercalation properties to host–guest molecules or ions,^5^ giving excellent catalytic activities,^4^ strong electron–electron correlations,^13^ outstanding phase transitions (metal–insulator transition),^14^ and high electrical conductivity.^5^ Furthermore, the abundant nanostructures of vanadium oxides can be achieved by different preparation methods, which not only shorten the transportation distance of ions or electrons and yield a faster solid-state diffusion in electrochemical energy conversion systems,^15,16^ but also provide more active positions for the interaction with other molecules or ions and more exposed active crystal facets for catalysis applications.^17,18^ Thus, the vanadium oxides provide promising applications in energy conversion/saving fields,^19,20^ such as ion batteries,^21−25^ water splitting,^26^ smart windows,^27,28^ supercapacitors,^29,30^ sensors,^31^ and so on.

A series of vanadium oxides with strong electron–electron correlations exhibit metal–insulator transition (MIT). The V_2_O_3_, VO_2_, and V_2_O_5_ with single oxidation undergo MIT at 160 K,^32^ 340 K,^33^ and 530 K,^34^ respectively. These phase transitions are reversible and accompanied by a change of crystallographic, magnetic, optical, and electrical properties. The mixed-valence vanadium oxides belong to either Magnéli series (V_*n*_O_2*n*-1_) or Wadsley series (V_*n*_O_2*n*+1_). For the Wadsley series, V_3_O_7_ and V_6_O_13_ exhibit the phase transition at 5.2 and 155 K, respectively.^35,36^ Except for V_7_O_13_ (metallic), all the Magnéli series show a transition from a paramagnetic to an antiferromagnetic state and consequently exhibit an antiferromagnetic ground state at low temperatures, including V_3_O_5_ (430 K), V_4_O_7_ (250 K), V_5_O_9_ (135 K), V_6_O_11_ (170 K), and V_8_O_15_ (70 K) with different phase transition temperatures, respectively.^37^

### 1.3 Scope of the Review

Vanadium oxides have a long history and rapid development in recent years as they are one of the most promising candidates in versatile applications in batteries, energy-saving smart windows, sensors, catalysts, optoelectronic devices, and so on. Therefore, many research papers and reviews have been published. Pioneering reviews on the chemistry of oxovanadium were published in 1965.^38^ The synthesis of vanadium oxides through hydrothermal and gas phase was reviewed by Whittingham,^39^ Livage,^40^ and Bahlawane.^41^ The atomic layer deposition of vanadium oxides was summarized by Papakonstantinou.^12^ The synthesis, properties, and applications of vanadium oxide nanotube were described by Kianfar.^6^ The catalytic applications of vanadium oxides have been described by Delferro,^3^ Hess,^42^ Carrero,^43^ and Granozzi.^44^ The Raman spectroscopy of vanadium oxides was recently reviewed by Shvets.^45^ The sensing properties of vanadium oxide nanostructures were described by Sheikhi^46^ and Madanagurusamy.^31^ The energy-related applications of vanadium oxides were reviewed by Xie,^5,20^ Jiang,^4^ and Streb.^47^ A large number of reviews described the progress in metal ions batteries, including those by Chen,^25^ O’Dwyer,^22^ Mai,^21,24,48^ Lowe,^23^ Whittingham,^49^ Rashada,^50^ Lee,^51^ Yang,^52^ Zheng,^53^ Kim,^54^ Liang,^55^ and Cao.^56^ The vanadium oxides in supercapacitors were reviewed by Chen,^30^ Dutta,^57^ and Li.^29^ Furthermore, our group also reviewed the multistimuli responsive properties and energy applications of some vanadium oxides.^19,27,58,59^

**Figure 1:**
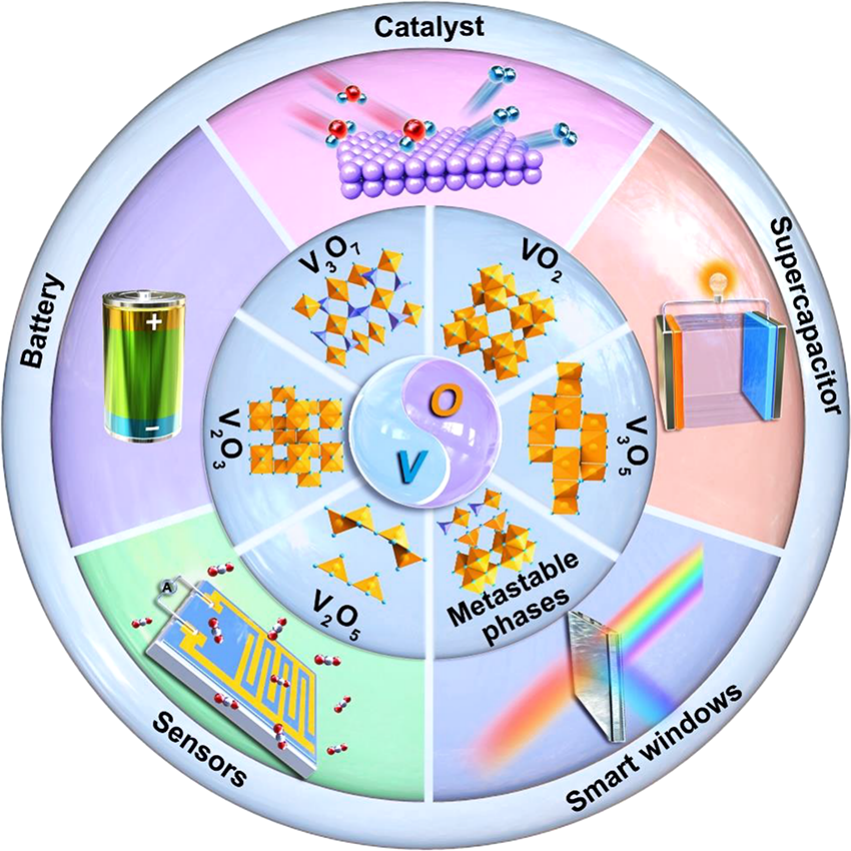
Schematic illustration of the thermodynamically stable vanadium oxides and their applications.

The great potential of vanadium oxides for new applications and accelerated industrialization led to the dramatically increase of the importance of vanadium oxides over the last 5–10 years. Furthermore, due to the complexity of various oxidation states of vanadium, vanadium oxides show a large variety of stable and metastable structures, which pose an inevitable challenge to synthesize vanadium oxides with high purity, well controlled stoichiometry, and meticulously designed nanostructures, a must for high performance devices. Even though lots of reviews have been published, most of them focus on a specific kind or application of vanadium oxides, such as batteries, spectra, supercapacitors, and so on. No comprehensive reviews illustrate the different vanadium oxides with different applications. In this review, we focus on the most recent progress on the structure, synthesis, and applications of five thermodynamically stable vanadium oxides (V_2_O_3_, V_3_O_5_, VO_2_, V_3_O_7_, V_2_O_5_) and some metastable vanadium oxides (V_2_O_2_, V_6_O_13_, V_4_O_9_, etc.), which can provide a better understanding of a specific vanadium oxide phase and their process-structure–property interrelationships. Figure 1 summarizes the main contents of this review. This review begins with the phase diagram of the V–O system to show the different vanadium oxide phases, followed by the vanadium oxides with different stoichiometries. For each vanadium oxide, the structures, synthesis methods, and applications in several fields will be covered. The last section presents the future prospects and a summary of this review.

## 2 Phase Diagram of the V–O System

The V–O binary phase diagram was compiled according to previously reported experimental data in 1989.^60^ The oxygen-rich phases are well-defined, which reveal more than 20 compounds.^41^ However, the vanadium-rich phases exhibit broad homogeneity ranges and high nonstoichiometry.^61,62^

**Figure 2:**
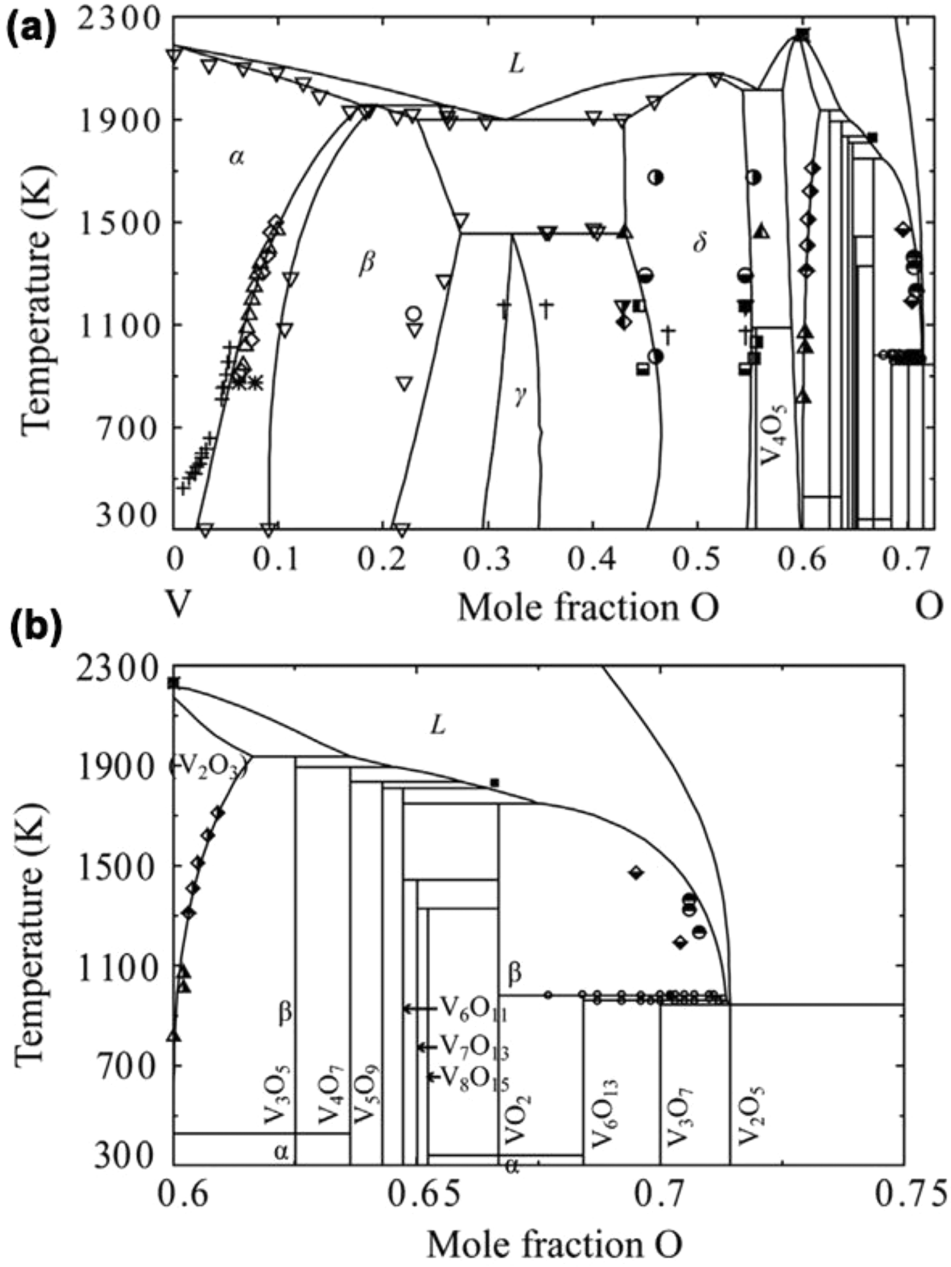
(a) Calculated phase diagram of the V–O system in the entire composition range at 1 atm. (b) Enlarged phase diagram of the V_2_O_3_–V_2_O_5_ system at 1 atm. Reproduced with permission from ref (61). Copyright 2015 Elsevier.

Several groups have calculated the V–O binary phase diagram.^61−64^Figure 2a shows a calculated phase diagram of the V–O system in the entire composition range at 1 atm. In the V-rich range, four types of solid solutions exist. The α and β solid solutions are formed by a certain amount of oxygen dissolved in the vanadium. The maximum solubilities of oxygen in α-V and β-V phase are up to 17.9 atom % and 27.4 atom %, respectively. The β-phase exhibits a wide range of homogeneities. With the increase of oxygen content, the γ and δ solid solutions phase can be formed. The γ-phase is monoclinic and δ-phase has the stoichiometry of VO with NaCl-type structure.

For the stoichiometric phases, only five phases are thermodynamically stable compositions as pure compounds, which include divanadium trioxide (V_2_O_3_, cubic, *Ia*3), trivanadium pentoxide (V_3_O_5_, monoclinic, *P*2/*c*), vanadium dioxide (VO_2_, tetragonal, *P*4_2_/*mnm*), trivanadium heptaoxide (V_3_O_7_, monoclinic, *C*2/*c*), and divanadium pentoxide (V_2_O_5_, orthorhombic, *Pmnm*). Other metastable phases or unstable phases are likely to decompose to stable phases. All the stable and metastable phases are listed in the enlarged phase diagram of the V_2_O_3_–V_2_O_5_ system, which is shown in Figure 2b.

There are two types of vanadium oxides with a mixed valence of vanadium. One is the Magnéli series, which is defined by the general stoichiometric formula:
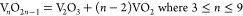


This type of homologous series has been reported for molybdenum oxides for the first time by Magnéli.^65^ The other homologous series is the Wadsley series, which has the general formula of V_*n*_O_2*n*+1_ (*n* ≥ 3). All the Magnéli phases maintain a triclinic symmetry (*P*1) and are metastable. They are expected to yield VO_2_ and V_3_O_5_ after lowering the system entropy. For the Wadsley series, V_3_O_7_ exhibits lower formation entropy compared with other Wadsley phases, which indicates the phase is more stable. Other Wadsley phases, such as V_4_O_9_ and V_6_O_13_, can be decomposed into a mixture of VO_2_ and V_3_O_7_.^41^ V_4_O_9_ and V_6_O_13_ show multiple metastable crystalline structures due to the close formation energies. Therefore, these compounds are candidates for polymorphism, where three crystalline structures were identified for V_6_O_13_ and two for V_4_O_9_.^60^

## 3 V_2_O_3_

### 3.1 Structures and Synthesis

**Figure 3:**
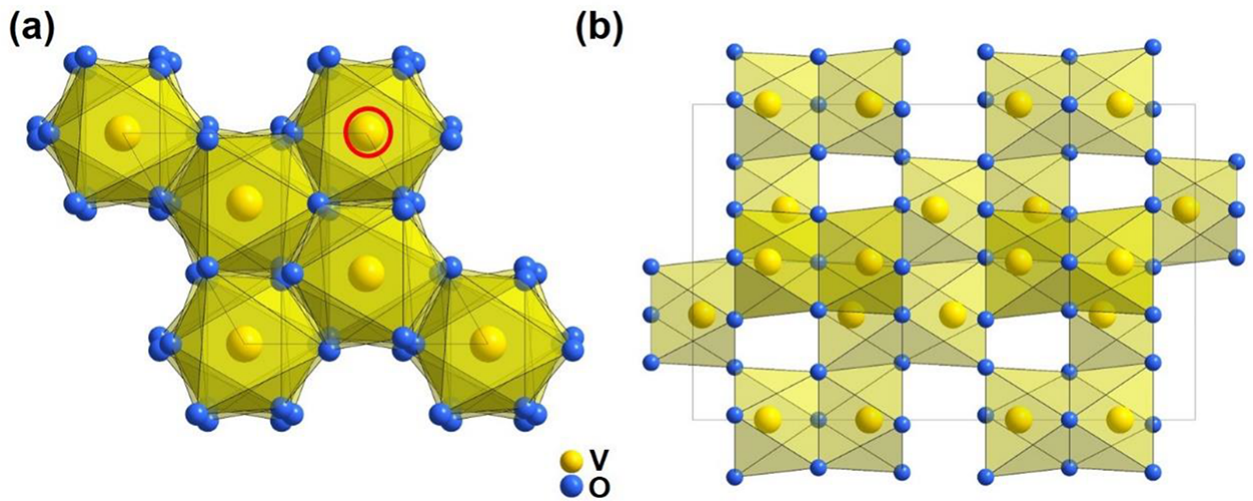
Perspective view of V_2_O_3_: (a) along the (001) direction; (b) along the (110) direction.

V_2_O_3_ has a typical corundum-type hexagonal structure (space group: *R*3̅*c*) with lattice parameters of *a* = *b* = 4.9492(2) Å, *c* = 13.988(1) Å at room temperature.^66,67^ The crystal structures of V_2_O_3_ viewed in different directions are illustrated in Figure 3. It is interesting that after being calcined at 600 °C for several hours, vanadium vacancies (red circle in Figure 3a) formed, which is suitable for aqueous zinc metal batteries.^67^ From another viewing direction, V_2_O_3_ possesses an open tunnel structure consisting of a 3D V–V framework (Figure 3b).^68^ Such tunnel structures could efficiently facilitate the insertion of alkali metal ions, which provide potential application in metal ion batteries.

V_2_O_3_ with different morphologies can be obtained by several methods, including reduction, oxidation, and hydrothermal approaches. For the reduction pathway, V_2_O_5_ was employed as initial materials, and hydrogen or ammonia gas was used as reducing agents, whereas vanadium metal was used as a starting material for the oxidation method. In the hydrothermal route, vanadium alkoxides or sulfides with some small organic molecules (e.g., thiourea, benzyl alcohol, etc.) were added together to the autoclave to grow V_2_O_3_.

**Figure 4:**
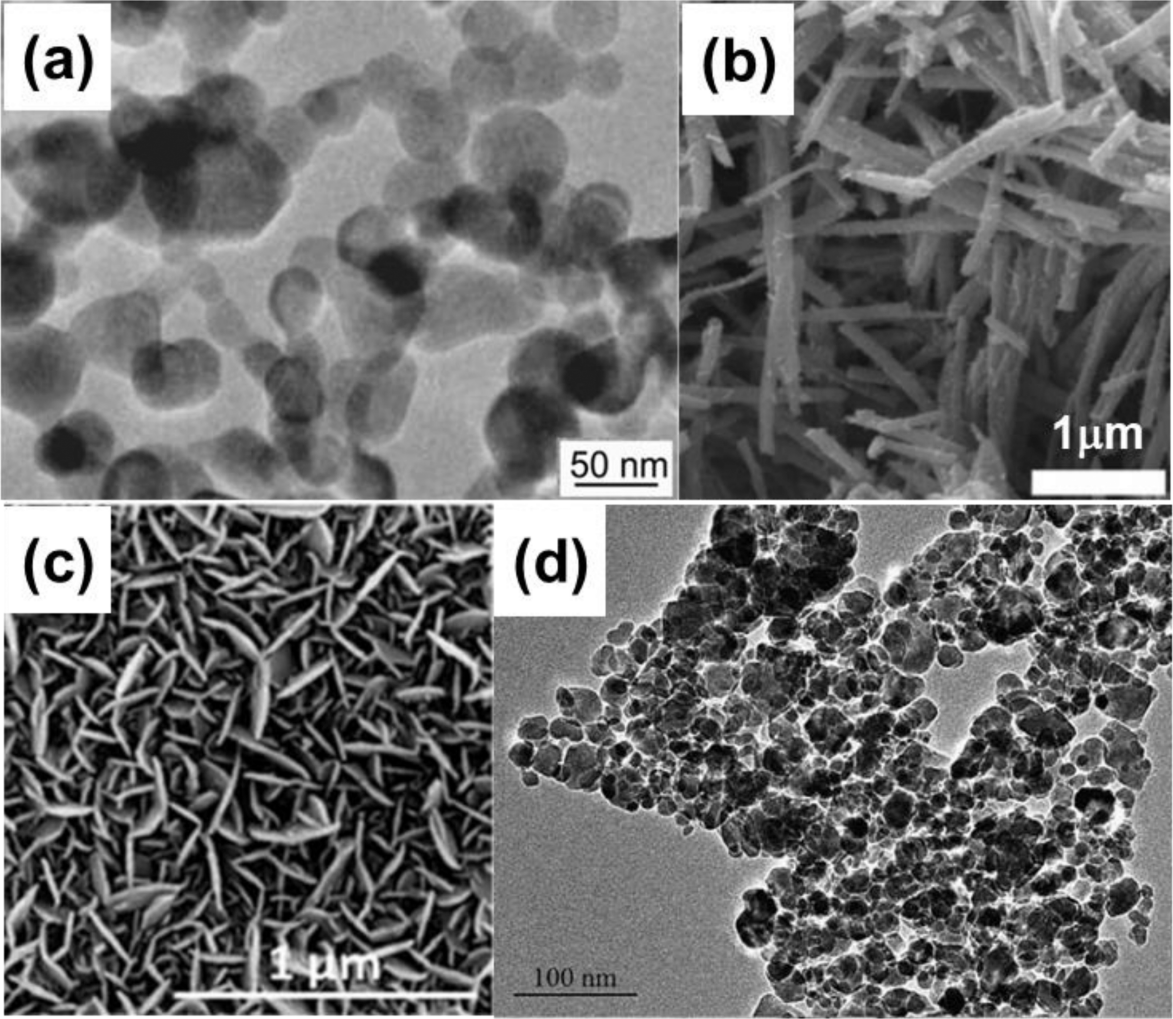
(a) Typical TEM image of V_2_O_3_ nanocrystals. Reproduced with permission from ref (69). Copyright 2012 Elsevier. (b) SEM image of V_2_O_3_ nanorods after reduction (time = 3 h and temperature = 600 °C). Reproduced with permission from ref (70). Copyright 2008 American Chemical Society. (c) SEM images of V_2_O_3_ nanosheets. Reproduced with permission from ref (73). Copyright 2018 Royal Society of Chemistry. (d) TEM of V_2_O_3_ nanocrystals. Reproduced with permission from ref (74). Copyright 2004 Elsevier.

Li et al.^69^ designed a plasma hydrogen reduction system to synthesize V_2_O_3_ nanocrystals via a single precursor of V_2_O_5_ powders. The coarse-grained V_2_O_5_ powders are injected into the hydrogen plasma by a powder feeder, reducing the V_2_O_5_ powders into V_2_O_3_ by hydrogen. Such single crystalline V_2_O_3_ nanocrystals have a spherical shape with average sizes in the range of 35–50 nm (Figure 4a). The morphology of the raw materials determines the V_2_O_3_ morphology. Seshadri et al.^70^ first synthesized the V_2_O_5_ nanorods via a hydrothermal reaction followed by reducing in 5% H_2_:95% N_2_ (reduction time = 3 h and reduction temperature = 600 °C) to obtain V_2_O_3_ nanorods (Figure 4b). The ammonia gas (NH_3_) is another agent to reduce V_2_O_5_ to V_2_O_3_.^71^ V_2_O_3_ shows a much larger size with several micrometers, and the morphologies of the V_2_O_3_ particles were micrometer layered structures that were assembled by nanometer or micrometer sheets. Tao et al.^72^ reported that the V_2_O_3_ nanoparticles had been synthesized by supercritical ethanol fluid reduction of VOC_2_O_4_ with an average size of 50 μm. Madanagurusamy et al. employed the oxidation way to obtain the V_2_O_3_ nanosheets.^73^ High-density vertically aligned V_2_O_3_ nanosheets on glass substrates were obtained via a simple one-step sputtering technique. Vanadium metal was first deposited on the well-cleaned glass substrates followed by oxidation in the argon and oxygen mixture gas with a ratio of 3:1. Well-ordered, ultrathin, vertically aligned V_2_O_3_ nanosheets with voids were obtained (Figure 4c).

Hydrothermal is one of the most popular methods for crystal growth, which is conducted under moderate temperature and a high vapor pressure environment in sealed containers with the unique advantages of being easy to handle and environmentally friendly. Compared with the reduction and oxidation methods, the hydrothermal reaction is more convenient for synthesis the V_2_O_3_ with various morphologies. Niederberger et al.^74^ adopted vanadium alkoxides and benzyl alcohol as precursors to synthesize V_2_O_3_ nanocrystals through hydrothermal reaction sizes ranging from 20 to 50 nm with good yields (Figure 4d). Without using any surfactant and template, Su et al.^75^ successfully synthesized dandelion-like V_2_O_3_ microspheres with core–shell structures. With increasing reaction time, the morphology of V_2_O_3_ can be tuned from a solid sphere to dandelion-like. If the reaction time is further increased, some broken V_2_O_3_ microspheres with core–shell structures could be observed with an average diameter of the core–shell microspheres of 2 μm.

### 3.2 Applications

#### 3.2.1 Batteries

**Figure 5:**
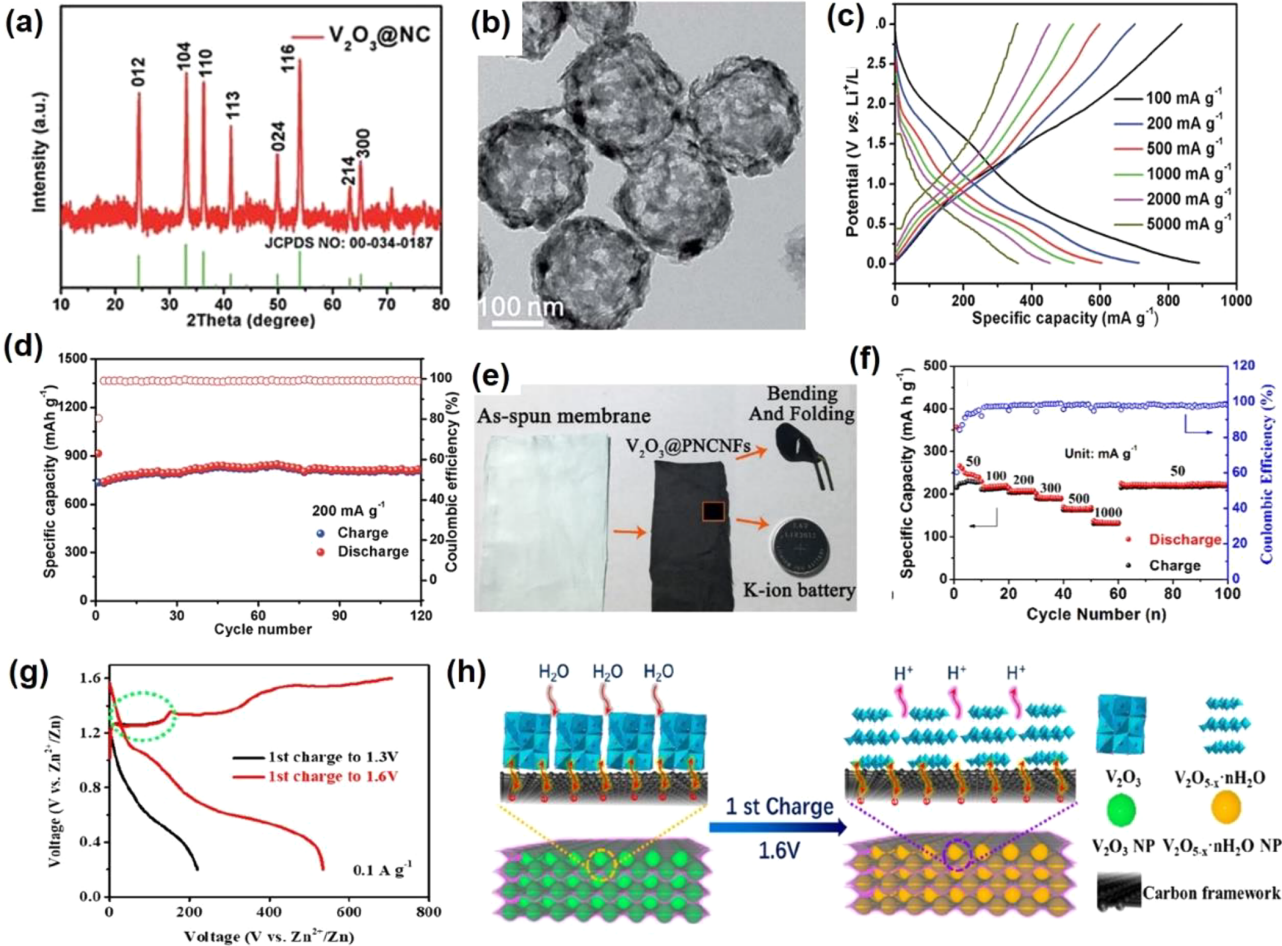
XRD pattern (a), SEM image (b), charge/discharge profiles (c), and cycling performance (d) of V_2_O_3_@NC hollow spheres. Reproduced with permission from ref (76). Copyright 2018 Royal Society of Chemistry. The digital photos of the as-spun membrane and the self-standing (e), and rate performance (f) of V_2_O_3_@PNCNFs. Reproduced with permission from ref (68). Copyright 2018 Elsevier. The charge/discharge profiles at different voltage window (g) and schematic illustration of the oxidation at full charge state (h) of V_2_O_3_. Reproduced with permission from ref (77). Copyright 2020 American Chemical Society.

As the low valence of V^3+^ in vanadium oxide, corundum-type V_2_O_3_ with a metallic behavior shows that the electrons in the V-3d orbital travel along the V–V chains. The 3D V–V framework provides an intrinsic tunnel structure, which is suitable for ion transport and intercalation. Mai et al. reported uniform nitrogen-doped carbon-confined V_2_O_3_ (V_2_O_3_@NC) hollow spheres (Figure 5a). The *in situ* carbonization hollow structure can provide high ion/electron conductivity, short diffusion distance, and excellent structure adaptability, which is beneficial for lithium-ion storage (Figure 5b). The V_2_O_3_@NC delivers an average discharge capacity of 785, 599, 528, and 361 mAh g^–1^ at a current density of 100, 500, 1000, and 5000 mA g^–1^, respectively (Figure 5c). Furthermore, 811 mAh g^–1^ can be maintained after 120 cycles at a current density of 200 mA g^–1^ (Figure 5d).^76^ In addition, V_2_O_3_ can also be used as other ions (Na^+^/K^+^/Zn^2+^) storage material. Jiao’s group^68^ fabricated a flexible and self-standing electrode of V_2_O_3_ nanoparticles embedded in porous N-doped carbon nanofibers (V_2_O_3_@PNCNFs) through electrospinning assisted high-temperature sintering method, which can directly be used as a KIB anode material (Figure 5e). V_2_O_3_@PNCNFs deliver a capacity of 240 and 134 mAh g^–1^ at a current density of 50 and 1000 mA g^–1^, respectively. High-capacity retention of 94.5% can be maintained after 500 cycles (Figure 5f). The density functional theory (DFT) results demonstrate that 1 mol K^+^ can insert into the V_2_O_3_ crystal forming KV_2_O_3_, with the K^+^ occupying the 6e sites of the KV_2_O_3_ crystal. The main capacity of V_2_O_3_ in KIB is main contribution from capacitance (surface or near-surface redox reactions), which is very different from the conversion reaction of conventional transition metal oxides for ion storage. When K^+^ inserts into the tunnels of V_2_O_3_@PNCNFs, the material exhibits no structural phase transitions, which indicates that V_2_O_3_@PNCNFs can exhibit excellent K^+^ rate/cycling performance. This work suggests that the pseudocapacitive electrode materials could be suitable for a large-scale energy storage system. Generally, the conventional low valent V_2_O_3_ cannot effectively accommodate Zn^2+^ intercalation during the discharging process due to its inherently unsuitable structure and inferior physicochemical properties. Luo et al.^77^ attempted to utilize V_2_O_3_ in aqueous ZIBs by the *in situ* anodic oxidation strategy, and the hierarchical microcuboid structure V_2_O_3_ can accommodate nearly 2 electrons intercalation. Moreover, they found H_2_O is a reactant that participates in the first charge oxidation process of V_2_O_3_. Furthermore, the porous structure with a higher specific surface area of the V_2_O_3_ leads to more reaction sites and a faster phase transition from V_2_O_3_ to V_2_O_5–*x*_·*n*H_2_O (Figure 5h). Meanwhile, the high surface and the small size of V_2_O_3_ nanoparticles benefit the first charge oxidation reaction process. The V_2_O_3_ delivers a Zn^2+^ discharging capacity of 625 mAh g^–1^ at 0.1 A g^–1^, corresponding to 1.75-electron intercalation (Figure 5g). Specifically, the capacities can maintain 87% and 78% when the current increases to 10 and 20 A g^–1^, respectively. The V_2_O_3_ can maintain 100% after 10000 cycles at 10 A g^–1^, which is better than some previously reported ZIB cathode materials.

#### 3.2.2 Catalysts

V_2_O_3_ and its composites have been widely used as catalysts for chemical looping dry reforming of methane,^78^ ammonium perchlorate decomposition,^79^ the hydrogen evolution reaction (HER),^30,80^ the oxygen evolution reaction (OER),^80^ water splitting,^80^ etc.

##### 3.2.2.1 Propane Dehydrogenation and Ammonium Perchlorate Decomposition

Very recently, Zhu et al.^81^ examined the catalytic properties of propane dehydrogenation through single transition metal atom doping of a V_2_O_3_ (0001) surface by self-consistent DFT calculation. The results indicated that the single atoms act as promoters and active sites, and Mn–V_2_O_3_ is a good candidate as a catalyst for propane dehydrogenation. Huang et al.^79^ synthesized V_2_O_3_ and V_2_O_3_/carbon composites by a facile hydrothermal route, which exhibited excellent performance for ammonium perchlorate decomposition. The decomposition temperature decreased by 49 and 73 K for V_2_O_3_ and V_2_O_3_/carbon composites, respectively.

##### 3.2.2.2 HER, OER, and Water Splitting

Electrocatalytic water splitting consisted of two half-reactions: HER and OER. Electrocatalysis is used to accelerate the rate of a chemical reaction through lowering the activation energy to reduce the electrochemical overpotentials.

**Figure 6:**
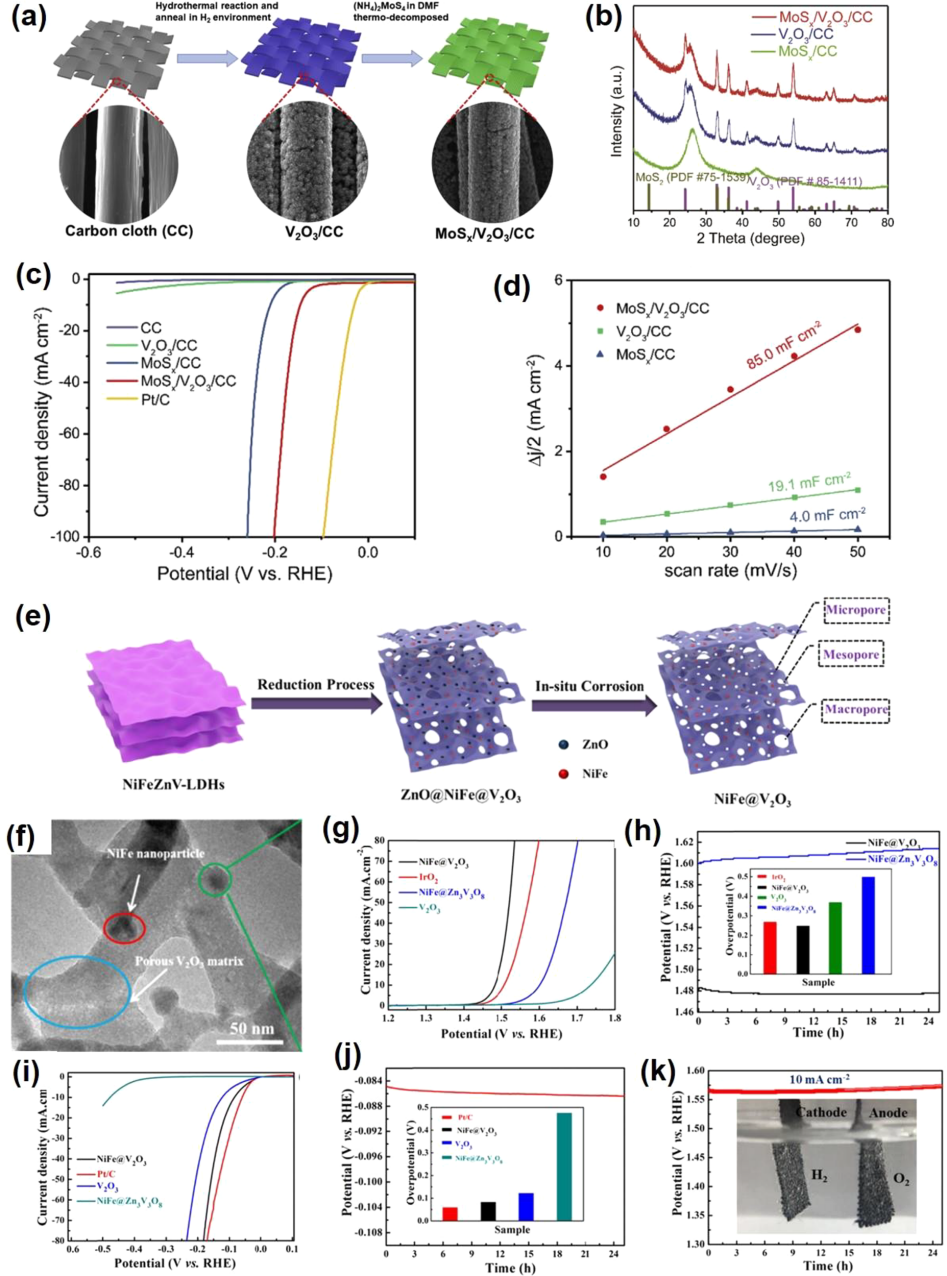
(a) Schematic diagram of the main steps in the synthesis of the MoS_*x*_/V_2_O_3_/CC. (b) XRD patterns of MoS_*x*_/V_2_O_3_/CC, V_2_O_3_/CC, and MoS_*x*_/CC. (c) Polarization curves for MoS_*x*_/V_2_O_3_/CC. (d) Double-layer capacitance for MoS_*x*_/V_2_O_3_/CC. Reproduced with permission from ref (82). Copyright 2020 Elsevier. (e) Synthetic strategy for preparing 3D hierarchical nanoporous NiFe@V_2_O_3_. (f) High-magnification TEM image of NiFe@V_2_O_3_. (g) OER polarization curves of NiFe@V_2_O_3_. (h) Long-term durability test conducted at 10 mA cm^–2^ of NiFe@V_2_O_3_. (i) HER polarization curves of NiFe@V_2_O_3_. (j) Long-term durability test conducted at 10 mA cm^–2^ of NiFe@V_2_O_3_. (k) Long-term potential-time curve set at 10 mA cm^–2^ of NiFe@V_2_O_3_||NiFe@V_2_O_3_ in 1.0 M KOH. Reproduced with permission from ref (80). Copyright 2019 American Chemical Society.

V_2_O_3_ composited with other functional materials can exhibit efficient electrocatalysis performance. Zhang et al.^82^ reported a self-supported MoS_*x*_/V_2_O_3_ heterostructure for the HER. Two steps were involved in the synthesis process (Figure 6a). V_2_O_3_/carbon cloth (CC) was first obtained by a hydrothermal method, and the dense V_2_O_3_ was uniformly distributed on CC fibers. The as-prepared V_2_O_3_/CC was immersed in an ammonium thiomolybdate solution and dried under a vacuum. Then, followed by a thermal decomposition process, the final MoS_*x*_/V_2_O_3_/CC was successfully achieved with the same morphology as V_2_O_3_/CC. The XRD pattern (Figure 6b) shows the composite contains the MoS_*x*_ and hexagonal V_2_O_3_. MoS_*x*_/V_2_O_3_/CC displayed an overpotential of 146 mV to achieve a 10 mA cm^–2^ HER current density (Figure 6c), which is lower than that of MoSx/CC (221 mV). The Tafel slope of MoS_*x*_/V_2_O_3_/CC is around 45 mV dec^–1^, suggesting the HER process obeys the Volmer-Heyrovsky mechanism. Meanwhile, the MoS_*x*_/V_2_O_3_/CC presents a higher double-layer capacitance (*C*_dl_: 85 mF cm^–2^) than MoS_*x*_/CC (4 mF cm^–2^), indicating V_2_O_3_ can create more active sites for the HER (Figure 6d). Furthermore, the MoS_*x*_/V_2_O_3_/CC electrocatalyst has great stability in the acid electrolyte for the HER. Two reasons for the MoS_*x*_/V_2_O_3_/CC electrocatalyst were given to understand the improved HER activity: (1) V_2_O_3_ enhanced the electrochemically active surface area with more active sites for the HER; (2) better electron transfer between MoS_*x*_ and V_2_O_3_. Qiu et al.^80^ synthesized V_2_O_3_ nanosheets anchored with NiFe nanoparticles as a bifunctional electrode for overall water splitting. A self-templated strategy was employed to synthesize the NiFe@V_2_O_3_ (Figure 6e). By a calcined reduction process, ZnO@NiFe@V_2_O_3_ nanosheets can be obtained. In-situ alkaline media corrosion was performed to dissolve the ZnO NPs and produce the porous NiFe@V_2_O_3_ nanosheets, which exhibit a clear V_2_O_3_ porous matrix and NiFe nanoparticles (Figure 6f). The porous NiFe@V_2_O_3_ exhibits good OER performance in an alkaline medium with an overpotential of 255 mV at 10 mA cm^–2^ and good stability (Figure 6g,h). Meanwhile, the NiFe@V_2_O_3_ also gave good HER performance in the same alkaline medium. It shows an overpotential of 84 mV at 10 mA cm^–2^ and good stability (Figure 6i,j). Furthermore, the porous NiFe@V_2_O_3_ delivers a small Tafel slope of 51 mV dec^–1^ for OER and a Tafel slope of 85.4 mV dec^–1^ for the HER, respectively. Considering the excellent HER and OER performance, two identical NiFe@V_2_O_3_ electrodes are integrated into a two-electrode cell to investigate the water splitting performance. The catalyst shows a cell potential of 1.56 V to reach 10 mA cm^–2^ with an ignorable cell voltage increase during 20 h measurements (Figure 6k).

The stability of the catalyst is a key parameter for practical applications, which is crucial to determine whether the catalyst can be commercialized.^83^ Several factors limit the catalytic stability, such as poor chemical/electrochemical stability of the catalysts under operation, abandoned gas evolution leading to the physical detachment of catalysts, dissolution of the catalysts in the electrolytes with different pHs, poor mechanical stability, and so on.^84−86^ For example, Schmidt et al.^87^ proved that the metal atoms are thermodynamic unstable during the OER process due to metal oxide released lattice oxygen, which leads to the low stability of the catalysts. From the theoretical perspective, there are three critical criterion of the catalysts that should be considered: excellent water dissociation performance, suitable Gibbs adsorption energy of H* (Δ*G*_H_*), and faster H_2_ desorption.^83^ Such properties enable the fast release of the active site without destroying the active site and further improve the stability of the catalysts.

#### 3.2.3 Supercapacitors and Electromagnetic Wave Absorber

**Figure 7:**
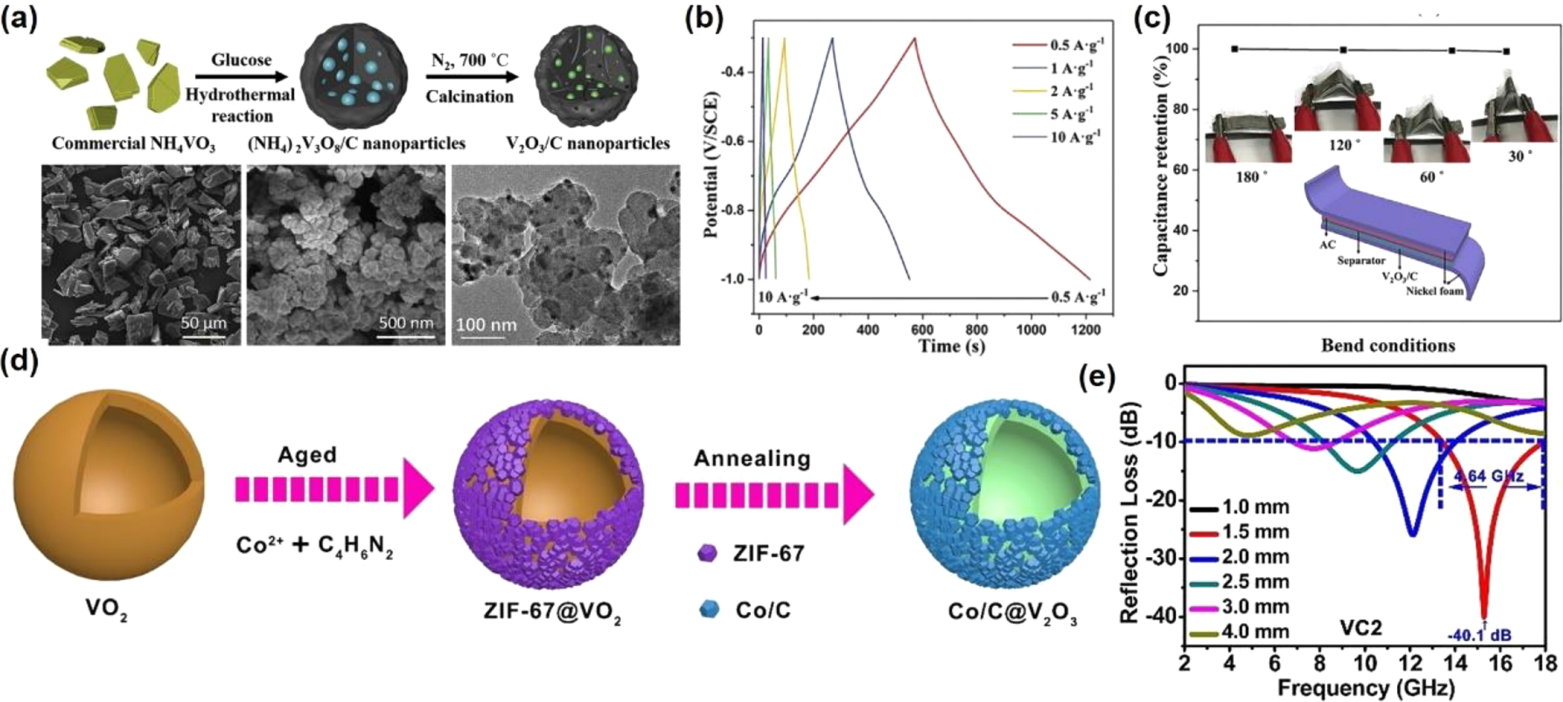
(a) A schematic illustration of the synthesis of highly dispersed VO-C. (b) GCD curves of VO-C collected at different current densities. (c) Capacitance retention of the VO-C||AC device measured under different bend conditions. Reproduced with permission from ref (93). Copyright 2019 Elsevier. (d) The synthesis process of hierarchical Co/C@V_2_O_3_ hollow spheres. (e) Reflection loss as a function of the frequency. Reproduced with permission from ref (95). Copyright 2019 John Wiley and Sons.

Supercapacitors (SCs) have attracted attention in recent years to bridge between a classic electrolytic capacitor and rechargeable battery, which are characterized by high power density and good cyclic stability. SCs are capable of storing electrical energy via two mechanisms: the electrochemical double layer capacitors having a nonfaradaic charge character and the pseudocapacitors based on faradaic electrochemical redox reactions.^88^ The theoretical specific capacitance of a pseudocapacitive electrode is proportional to the number of electrons involved in a specific redox reaction, and vanadium oxides possess four readily accessible valence states, making vanadium oxides especially promising for high pseudocapacitance.^89^ The conductivity of V_2_O_3_ (∼10^3^ Ω^–1^ cm^–1^) is higher than monoclinic VO_2_ (∼4 Ω^–1^ cm^–1^) and comparable with Ru (∼10^4^ Ω^–1^ cm^–1^).^90^ Meanwhile, V_2_O_3_ is stable in both acid and base mediums.^91^ Thus, V_2_O_3_ is a suitable material for energy storage, especially for SCs. However, the reported specific capacitances of V_2_O_3_-based materials are not good enough, suggesting that design and synthesis of new structured V_2_O_3_-based materials with high performance are required.^92^ Cao et al.^93^ synthesized V_2_O_3_ nanoparticles highly dispersed in amorphous carbon composites through the calcination of the (NH_4_)_2_V_3_O_8_/C precursor, which was fabricated through the hydrothermal reaction by using commercial NH_4_VO_3_ and glucose (Figure 7a). The as-prepared V_2_O_3_-based composites exhibit a specific capacitance of the electrode of 458.6 F g^–1^ at a current density of 0.5 A g^–1^ (Figure 7b), which is higher than other reported V_2_O_3_-based composites.^94^ Meanwhile, the asymmetric supercapacitors assembled by the as-prepared V_2_O_3_-based composites display good flexibility properties (Figure 7c). The capacitances are almost constant with the bending from 180° to 30°. The V_2_O_3_-based composites are one kind of high-efficiency electromagnetic wave absorber. Yan et al.^95^ produced the hierarchical Co/C@V_2_O_3_ hollow spheres by hydrothermal, aging, and annealing methods (Figure 7d). The VO_2_ spheres were first fabricated through a hydrothermal method followed by coating with ZIF-67 by precipitation. After that, the ZIF-67@VO_2_ composite was calcined in H_2_/Ar to form the hierarchical Co/C@V_2_O_3_ hollow spheres. The Co/C@V_2_O_3_ hollow spheres exhibited excellent electromagnetic wave absorption performance with a reflection loss of @40.1 dB and a broad bandwidth of 4.64 GHz at a small thickness of only 1.5 mm (Figure 7e). The good performance is mainly due to the impedance matching and low density, which come from the combination of hollow V_2_O_3_ spheres and porous Co/C.

## 4 V_3_O_5_

### 4.1 Structures and Synthesis

**Figure 8:**
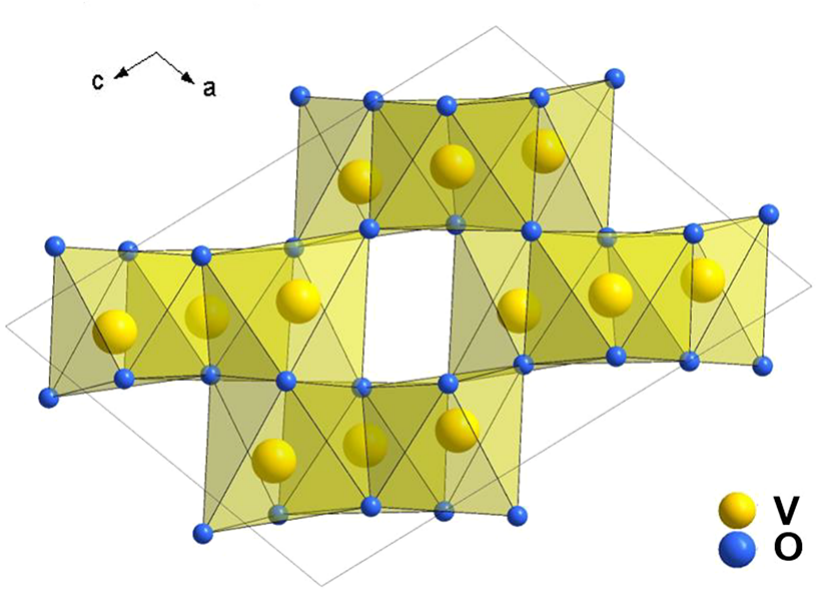
Crystal structure of V_3_O_5_.

V_3_O_5_ crystal structure was first reported in 1954,^96^ which possesses a monoclinic symmetry (space group *P*2/*c*).^97^ The crystal structure is shown in Figure 8. The oxygen atoms are occupied in the octahedral sites, and vanadium atoms are located in the center of octahedron. There are two types of octahedra chains along the *c* axis: face-shared octahedra via edge-sharing and corner-sharing octahedra. Such chains formed a framework with many large open spaces, which is capable of accommodating lithium ions.^98^

It is difficult to stabilize the V_3_O_5_ phase by using solid-state chemistry and to control the stoichiometry between oxygen and vanadium by using a solution method, which makes V_3_O_5_ an uncommon phase used for electrochemical and other applications.^98,99^ Therefore, the reported synthetic method for V_3_O_5_ is very limited. Reduction of V_2_O_5_ by different reductants is commonly used to obtain the V_3_O_5_ polycrystalline powders. Reisner et al.^100^ selected vanadium metal as a reductant to reduce V_2_O_5_, and the V_3_O_5_ polycrystalline powders were synthesized according to the following chemical reaction: V + V_2_O_5_ → V_3_O_5_. Vanadium powder and V_2_O_5_ powder were mixed together and pressed into bars, which were sealed in a quartz ampule and heated for 24 h at 870 K and for 100 h at 1220 K. The V_3_O_5_ polycrystalline powders exhibit a size around 10 μm. Alternatively, Yu et al.^98^ used sulfur powders as a reductant. The mixture of sulfur and V_2_O_5_ powders were vigorously grounded and calcined at 1023 K in a tube furnace under a vacuum for 2 h. The V_3_O_5_ microcrystals can be obtained by washing the calcined powders with nitrogen tetrasulfide several times. The obtained V_3_O_5_ microcrystals range from 1 to 3 μm. The large size single crystal of V_3_O_5_ was grown by chemical vapor transport in the 1970s.^101,102^ V_3_O_5_ powders or V_2_O_3_–VO_2_ mixed powders were used as starting materials, and TeCl_4_ was used as a transport agent. Both starting materials and transport agent were sealed in a quartz tube with low pressure (∼1 × 10^–5^ mbar), and then the tube was put into a two-zone furnace. Generally, the source zone has higher temperature, while the crystallization zone has a lower temperature, and the temperature gradient is around 100 to 150 K. The size of final V_3_O_5_ single crystal is as large as 1 cm.

### 4.2 Applications

#### 4.2.1 Batteries

**Figure 9:**
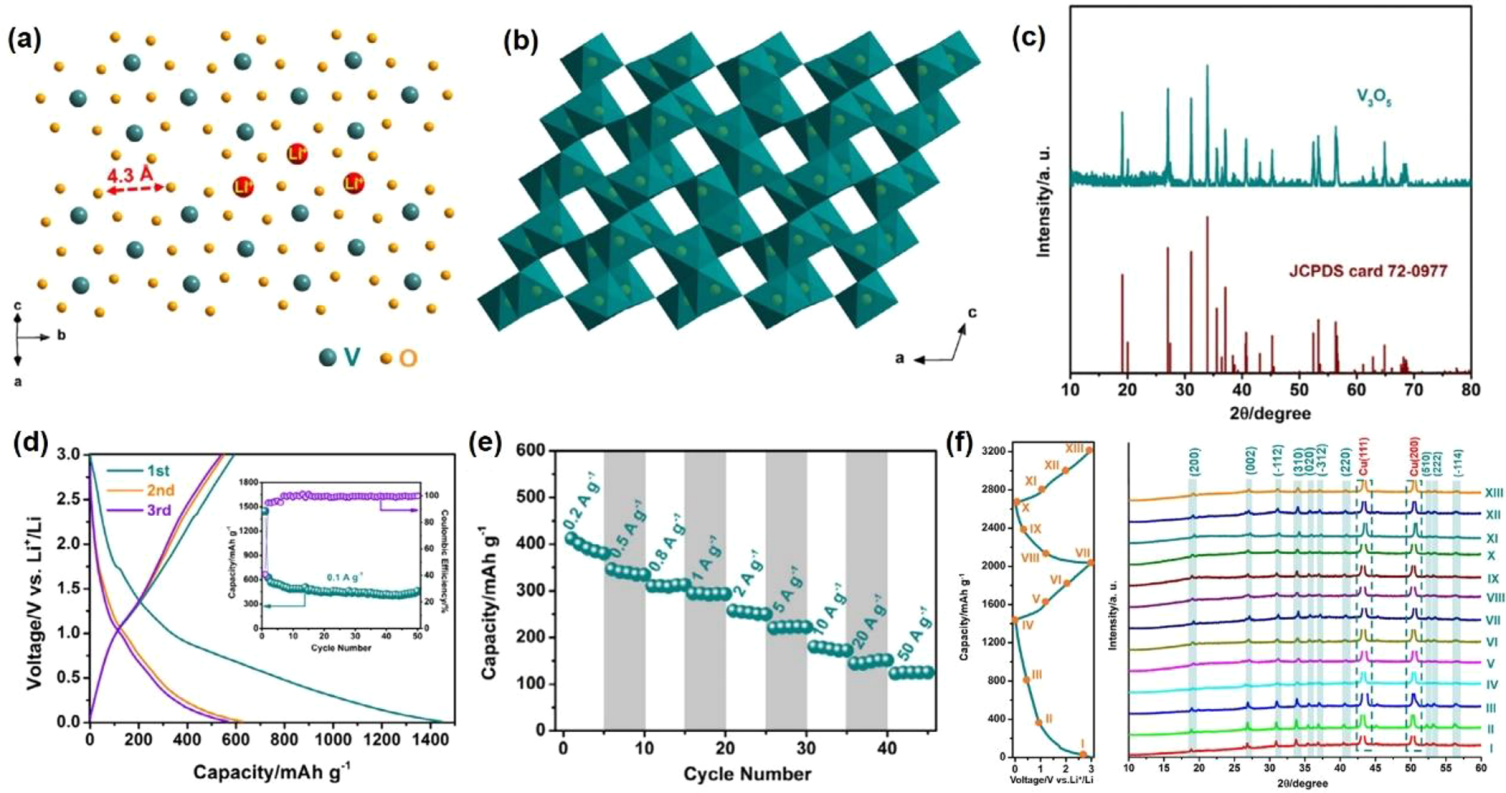
[101] projection illustrating the hexagonal close packing of oxygen atoms (a), the connection of VO_6_ octahedral (b), the XRD pattern (c), charge/discharge profiles (inset: corresponding cycling performance at 100 mA g^–1^) (d), rate performance (e) and *ex-situ* XRD patterns (f) of V_3_O_5_. Reproduced with permission from ref (98). Copyright 2019 under CC BY license.

V_3_O_5_ is relatively less-studied though it exhibits a three-dimensional open-framework structure, which is due to the strict synthesis condition. Yu’s group^98^ successfully synthesized V_3_O_5_ microcrystals via vacuum calcination and first employed it as an LIB anode material. The oxygen atoms are closely arranged in a hexagonal shape, and the vanadium atoms take up three-fifths of the octahedral interstices (Figure 9a). The 3D open-framework structure of V_3_O_5_ is formed with chain connections, which contain distorted, sharing corners, edges, and faces of VO_6_ octahedral, respectively (Figure 9b). The 3D framework of V_3_O_5_ with much large open space endows the capacity of Li^+^ intercalation/deintercalation. No impurity peaks from the XRD pattern are observed (Figure 9c), which indicates that the V_3_O_5_ powder shows a single-phase nature with a monoclinic structure within the *P*2/*c* space group (JCPDS card 72-0977). It delivers a high capacity of 628 mAh g^–1^ at 100 mA g^–1^, a good rate (125 mAh g^–1^ at 50 A g^–1^), and a long stable cycling performance (117 mAh g^–1^ after 2000 cycles) (Figure 9d,e). There is no obvious crystal structure change of the V_3_O_5_ with Li^+^ intercalation, which is the main reason for the good rate and cycling performance (Figure 9f).

#### 4.2.2 Other Applications

V_3_O_5_ thin films exhibit a photoinduced insulator-to-metal phase transition, which results in a strong nonlinear optical response.^103−105^ Fernández et al.^104^ deposited the V_3_O_5_ directly on the SiO_2_ substrates by DC magnetron sputtering to form thin films. The ultrafast nonlinear optical response was probed by using a pump–probe scattering technique. A reduction in the transient relative scattered light signal was observed, which showed an ∼10% decrease within 800 fs. Such a response is due to the changes in the material’s optical constants and very likely related to the photoinduced insulator-to-metal phase transition.^106^ The photoinduced screening of electron correlations followed by melting of polaronic Wigner crystal and coalescence of V–O octahedra is the main reason for the order–disorder structural transition.^103^

## 5 V_3_O_7_

### 5.1 Structures and Synthesis

**Figure 10:**
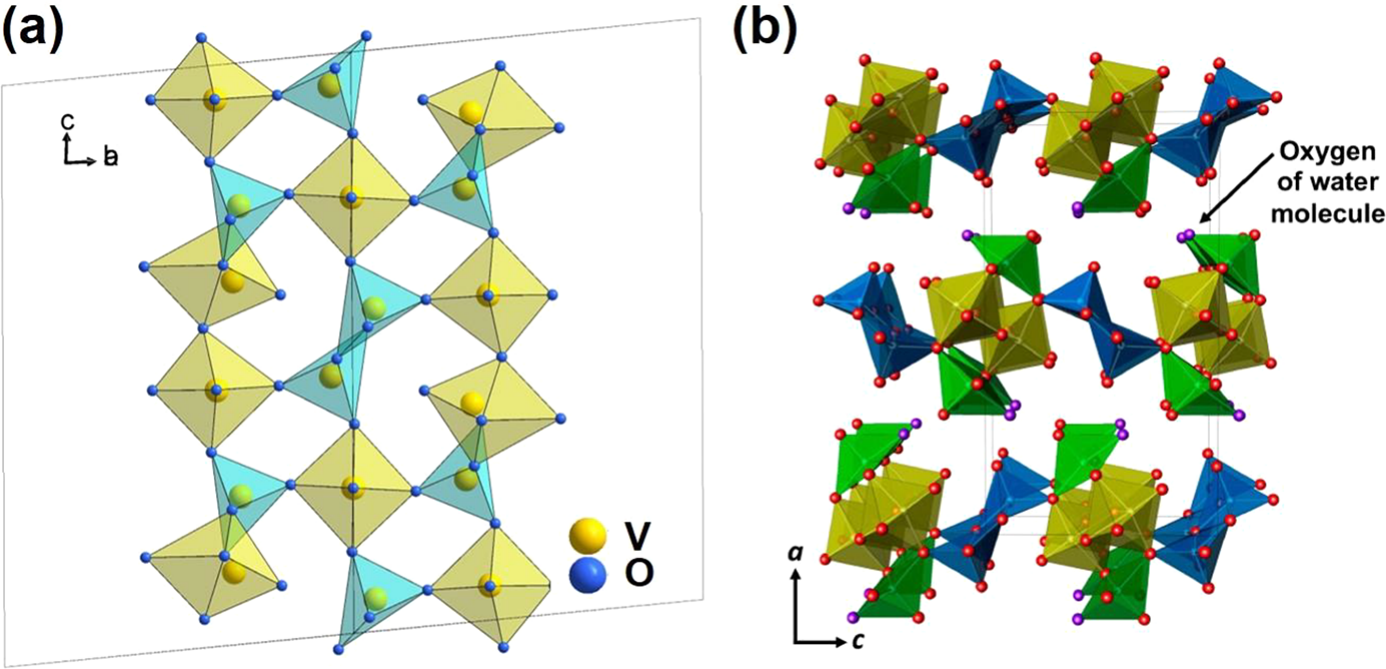
(a) Crystal structure of V_3_O_7_ with VO_6_ octahedra (yellow) and VO_5_ (blue). (b) Crystal structure of V_3_O_7_·H_2_O (H_2_V_3_O_8_), with VO_6_ octahedra (yellow) and VO_5_ (green) and VO_5_ (blue) polyhedra. Hydrogen atoms are bonded to the oxygen atoms (purple), which are not shown. Reproduced with permission from ref (108). Copyright 2018 American Chemical Society.

Figure 10a shows the crystal structure of V_3_O_7_. The unit cell contains 36 vanadium atoms (12 vanadium atoms are inside the octahedra and 24 vanadium atoms are five-coordinated).^107^ The V_3_O_7_ consists of VO_6_ octahedra and VO_5_ polyhedra, which are linked by corners and edges to form a three-dimensional framework. The crystal structure of V_3_O_7_·H_2_O shows a two-dimensional structure compared with V_3_O_7_ (Figure 10b). Each V_3_O_8_ layer consists of corner- or edge-shared VO_6_ octahedra and VO_5_ polyhedra.^108^ The water molecules are located at the sides of the V_3_O_8_ layer, where the hydrogen atoms are directly bonded to the vanadium atom of a VO_5_ polyhedron and hydrogen bonds connect two neighboring V_3_O_8_ layers.^7,109^

**Figure 11:**
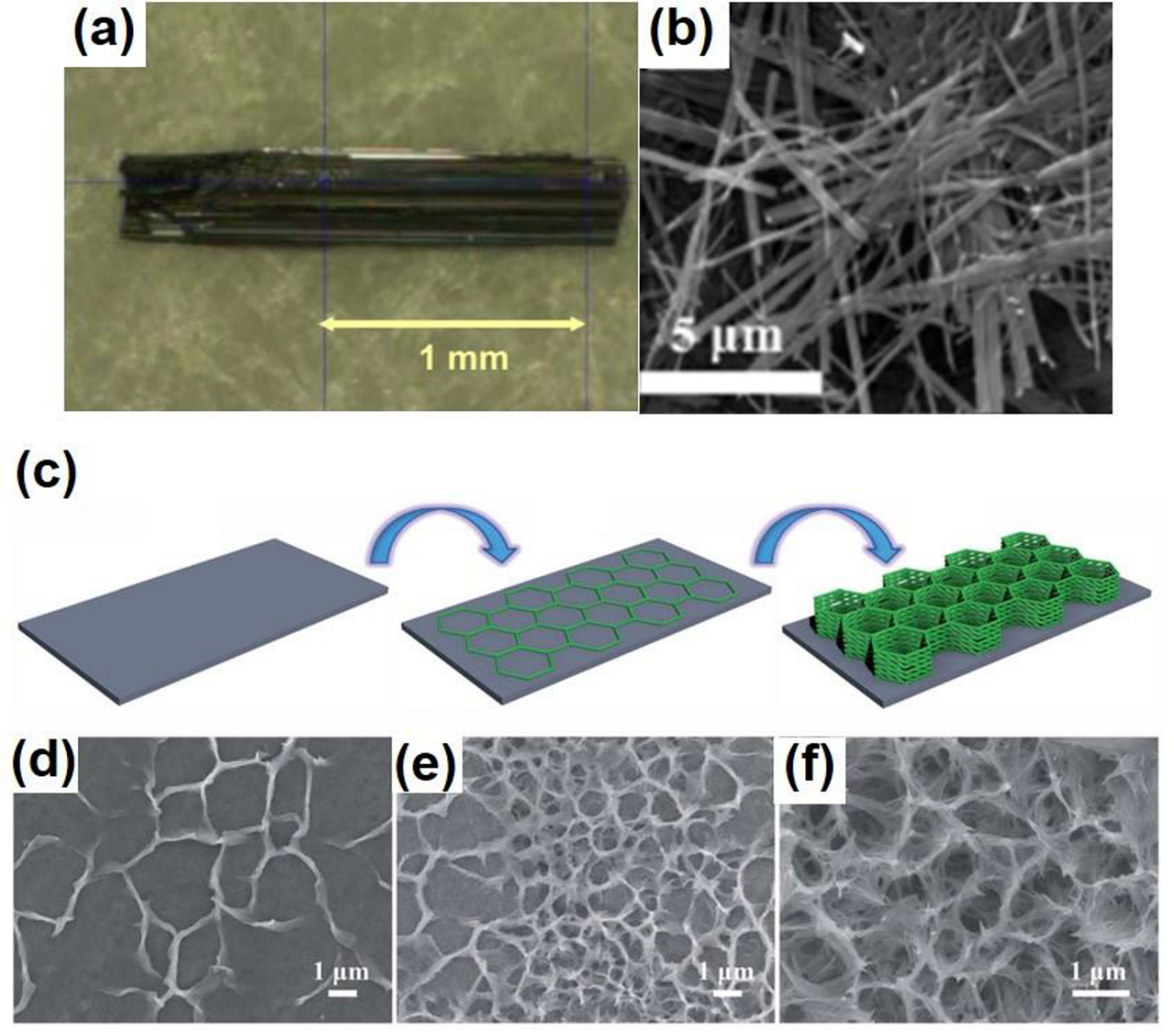
(a) Optical images of bulk V_3_O_7_ single crystals. Reproduced with permission from ref (110). Copyright 2009 Elsevier. (b) The SEM image of V_3_O_7_ nanobelts obtained through a soft chemical topotactic synthesis and hydrothermal process. Reproduced with permission from ref (111). Copyright 2016 Royal Society of Chemistry. (c) Schematic synthesis process for nest-like V_3_O_7_. (d–f) SEM images of V_3_O_7_ prepared with the hydrothermal times of 1, 5, and 10 h. Reproduced with permission from ref (112). Copyright 2018 Royal Society of Chemistry.

Bulk V_3_O_7_ single crystal can be grown by a typical chemical vapor transport method using V_3_O_7_ polycrystalline powders as starting materials and NH_4_Cl as a transport agent.^110^ The mixed V_3_O_7_ polycrystalline powders and NH_4_Cl were pressed into a pellet and heated at 823 K in an evacuated silica tube for 7 days. The black needle-like single crystals of V_3_O_7_ were formed with a length of 2 mm (Figure 11a). Nanostructured V_3_O_7_ was generally obtained by a typical hydrothermal method. The precursor solution dramatically affects the morphology of the V_3_O_7_ nanostructures. Wen et al. combined a soft chemical topotactic synthesis and hydrothermal process to prepare V_3_O_7_ nanobelts.^111^ In the beginning, layered structured KV_3_O_8_ plate-like particles were first prepared as the precursor by a hydrothermal method of V_2_O_5_ and KOH. The H^+^-form vanadate (HVO) nanobelt colloidal solution was subsequently obtained by reacting KV_3_O_8_ plate-like particles with HNO_3_. Finally, the one-dimensional (1D) pure single crystal V_3_O_7_ nanobelts were successfully prepared after hydrothermally treating the colloidal solution at 180 °C for 12 h (Figure 11b). By using NH_4_VO_3_ and HCl as starting materials, the nest-like V_3_O_7_ self-assembled by porous V_3_O_7_ nanowires on Ti foil was also synthesized through a hydrothermal method.^112^ In the beginning, a layer of V_3_O_7_ nanosheets was deposited on the Ti substrate, which was subsequently placed in the NH_4_VO_3_–HCl solution and kept at 160 °C for 10 h for a hydrothermal method. With the increase of hydrothermal time, the nest-like V_3_O_7_ is self-assembled by nanowires. The schematic synthesis process and SEM images for nest-like V_3_O_7_ are shown in Figure 11c–f. The V_3_O_7_ fibers can be obtained by thermal treatment of the electrospun NH_4_VO_3_/PVP nanofibers in the presence of reductant.^113^ The thermal treatment condition dramatically affects the final products, and V_2_O_5_ may be obtained together with V_3_O_7_.

**Figure 12:**
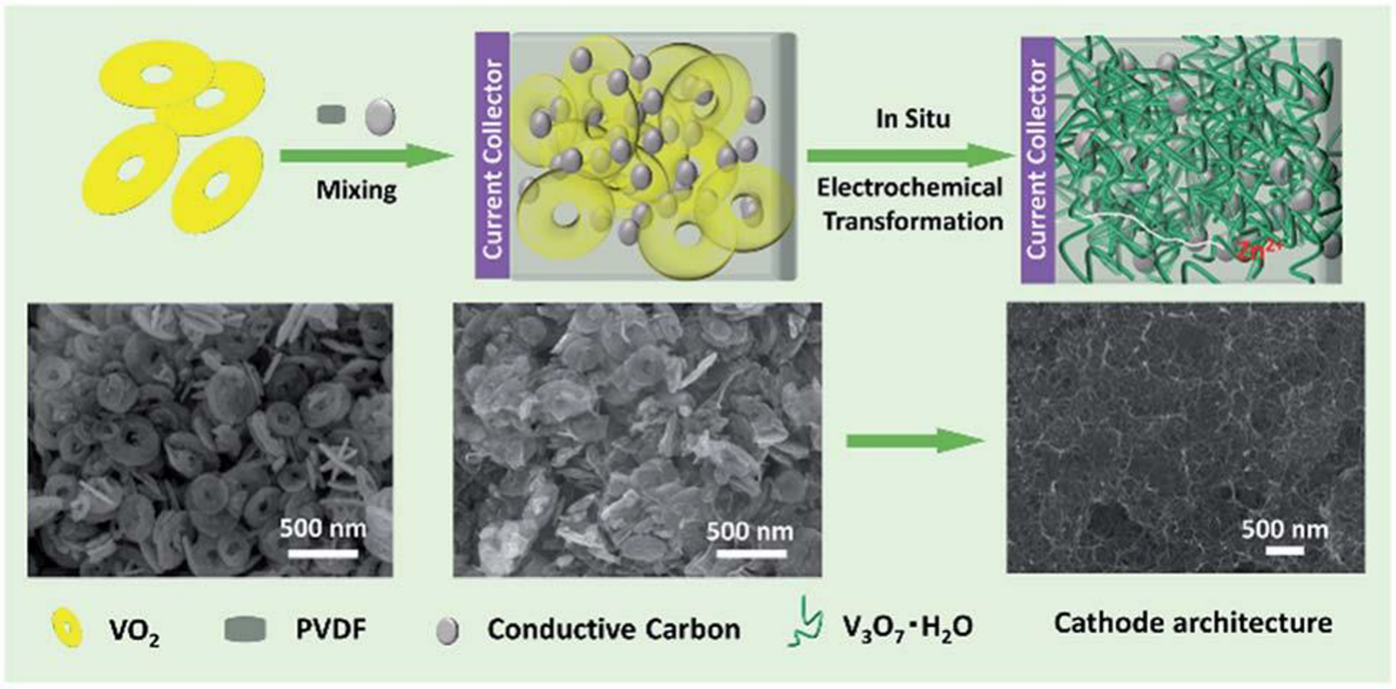
Schematic illustration of the preparation of the nanogrid-shaped V_3_O_7_·H_2_O. Reproduced with permission from ref (119). Copyright 2019 Royal Society of Chemistry.

The hydrothermal reaction is also a facile way to synthesize V_3_O_7_·H_2_O nanostructures. The V_3_O_7_·H_2_O nanobelts can be achieved by using V_2_O_5_, phenolphthalein and distilled water as starting materials through a hydrothermal method for 4 days of reaction at 180 °C.^114^ By changing phenolphthalein to ethanol or glucose, V_3_O_7_·H_2_O nanobelts were also successfully synthesized at 180 °C, while the reaction time was shortened to 12 h.^115,116^ In above-mentioned the methods, phenolphthalein, ethanol, or glucose is used as the reductant. Without the reductant, the V_3_O_7_·H_2_O nanobelts or nanowires can be obtained by a hydrothermal reaction of only V_2_O_5_ or NH_4_VO_3_.^117,118^ The key point is the pH of the precursor solution. After adjusting the pH to 3 by adding concentrated HCl, V_3_O_7_·H_2_O nanobelts and nanowires were obtained by a reaction at 190 °C for 24 h and 160 °C for 3 h, respectively. Furthermore, ultrafine V_3_O_7_·H_2_O nanogrids can be obtained through electrochemical oxidation.^119^ In the beginning, the nsutite-type VO_2_ black powder was synthesized by a hydrothermal method. Then, a three electrode system was employed to electrochemically transform the VO_2_ precursor to V_3_O_7_·H_2_O. The slurry, consisting of VO_2_ nanoplates, was coated on the working electrode. With a constant current density of 50 mA cm^–2^ and a cutoff potential of 1.7 V, V_3_O_7_·H_2_O nanogrids were obtained (Figure 12).

### 5.2 Applications

#### 5.2.1 Batteries

**Figure 13:**
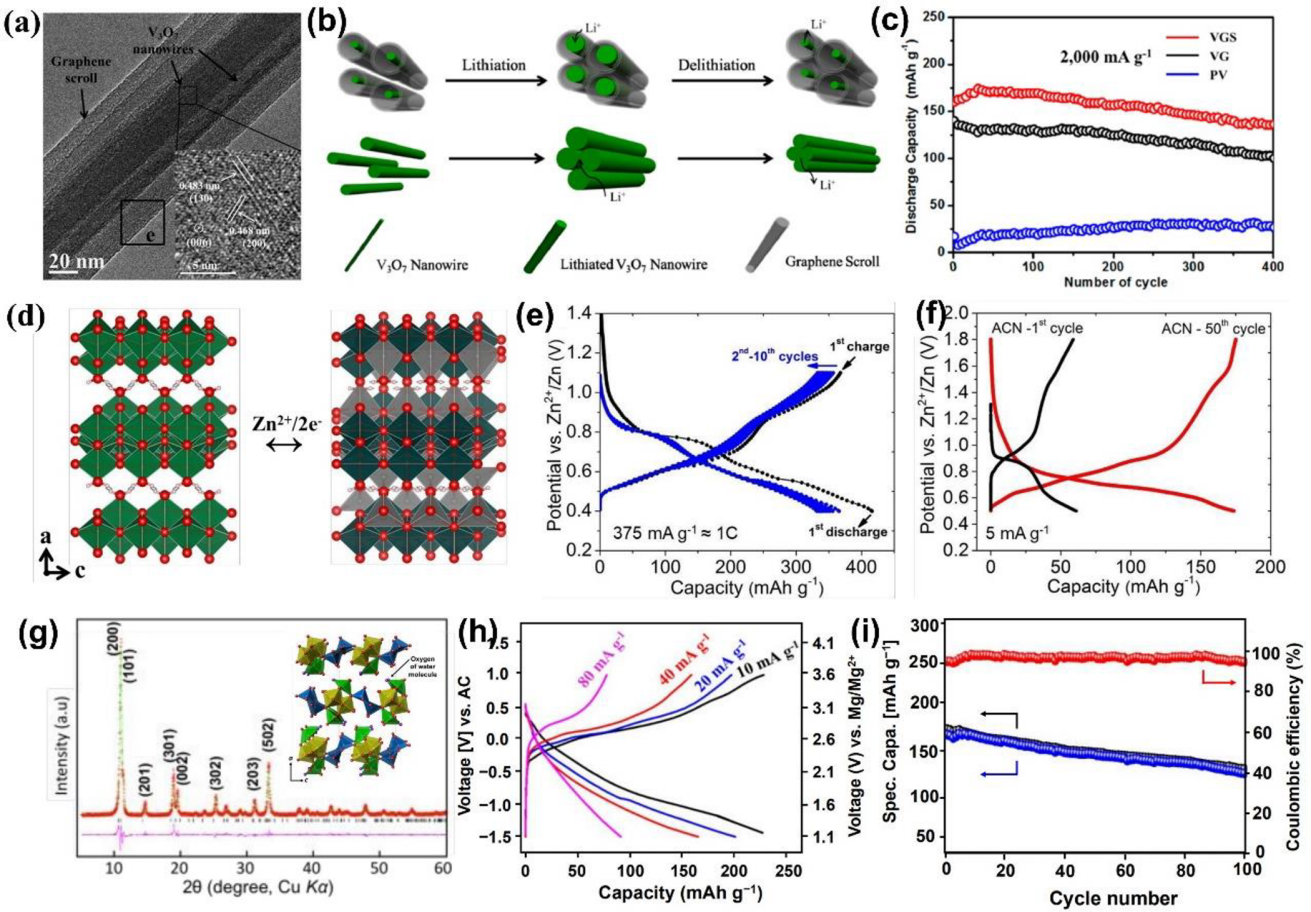
TEM image of VGS (the inset gives an HRTEM image of a V_3_O_7_ nanowire in graphene scrolls) (a), schematic of the VGS nanoarchitecture with continuous electron and Li ion transfer channels (b), and cycling performances (c) of VGS, V_3_O_7_ nanowire/graphene, and V_3_O_7_ nanowire. Reproduced with permission from ref (120). Copyright 2013 American Chemical Society. The schematic illustration of reaction mechanism (d), charge/discharge profiles in aqueous (e), and nonaqueous (f) electrolytes of Zn//V_3_O_7_·H_2_O batteries. Reproduced with permission from ref (121). Copyright 2018 Royal Society of Chemistry. The powder X-ray Rietveld refinement profile and crystal structure (g), charge/discharge profiles (h), and cycling performance (i) of V_3_O_7_·H_2_O nanowires in MIBs. Reproduced with permission from ref (108). Copyright 2018, American Chemical Society.

V_3_O_7_ is a mixed-valence vanadium oxide for metal-ion storage. Yan et al.^120^ designed and synthesized a V_3_O_7_ nanowire templated graphene scroll (VGS) via an “oriented assembly” and “self-scroll” strategy. They used joint experimental-MD simulation to investigate the construction and formation mechanisms of VGS. The systemic energy, the curvature of nanowires, and the reaction time determined the length and formation process of the semihollow bicontinuous structure. Through this strategy, the VGS with a length up to 30 μm has interior cavities between the nanowire and scroll (Figure 13a). The unique structure of VGS with the nanowire templated graphene scroll offers a continuous Li^+^/ion transfer channel and free volume expansion space during Li^+^ de/intercalation (Figure 13b). The VGSs exhibit a high capacity of 321 mAh g^–1^ and good cycle stability (87.3% after 400 cycles), which is better than the pure V_3_O_7_ nanowire and V_3_O_7_ nanowire/graphene structure (Figure 13c).

In addition, Nazar et al.^121^ synthesized layered V_3_O_7_·H_2_O nanobelts with single crystalline via a microwave solvothermal method and applied it to a ZIB cathode in nonaqueous and aqueous electrolytes. The electrochemical studies and *in situ* XRD results demonstrate the different electrochemical behaviors of layered V_3_O_7_·H_2_O in nonaqueous and aqueous electrolytes. Combining the DFT calculations, ∼2 mol Zn^2+^ can insert into V_3_O_7_·H_2_O per the formula in the ZnSO_4_/H_2_O aqueous electrolyte (Figure 13d). The V_3_O_7_·H_2_O delivers a capacity of 400 mAh g^–1^ (>2 mol Zn^2+^ insertion) with an average voltage of ∼0.65 V at the first discharge process, and maintains 375 mAh g^–1^ at the subsequent charge process (Figure 13e), while in the Zn(CF_3_SO_3_)_2_/acetonitrile nonaqueous electrolyte, the V_3_O_7_·H_2_O exhibits a poor Zn^2+^ storage performance (59 and 175 mAh g^–1^ for the first and 50th cycles at 5 mA g^–1^, respectively) (Figure 13f).

Magnesium-ion battery (MIB) as another multivalent ion battery has been attracting more attention due to its high abundance in the Earth and low redox potential (−2.37 V vs. SHE). V_3_O_7_·H_2_O with high electronic conductivity (V^+4.67^) has been widely used as a cathode material in LIB/NIB and hybrid Li^+^/Mg^2+^ batteries. Hong et al.^108^ synthesized V_3_O_7_·H_2_O nanowires via a one-step hydrothermal method and applied them to a high-energy MIB cathode (Figure 13g). The electrochemical tests and structural characterization results demonstrate that the structured water in V_3_O_7_·H_2_O will remain stable during the cycling. 0.97 mol Mg^2+^ inserts into V_3_O_7_·H_2_O, accompanying the formation of Mg_0.97_H_2_V_3_O_8_ at the first discharged state. V_3_O_7_·H_2_O exhibits an initial discharge capacity of 231 mAh g^–1^ at 10 mA g^–1^ with an average discharge voltage of ∼1.9 V, and the energy density can reach 440 Wh kg^–1^ (Figure 13h). Meanwhile, V_3_O_7_·H_2_O delivers a 171 mAh g^–1^ and maintains 132 mAh g^–1^ (77%) after 100 cycles at 40 mA g^–1^ (Figure 13i). The excellent Mg^2+^ storage performance is attributed to the unique crystal structure with direct bonding. This strategy of applying water-metal bonding and hydrogen bonding provides a new idea to search for new oxide-based MIB materials with stable and high energy density.

#### 5.2.2 Ammonium Perchlorate Decomposition

**Figure 14:**
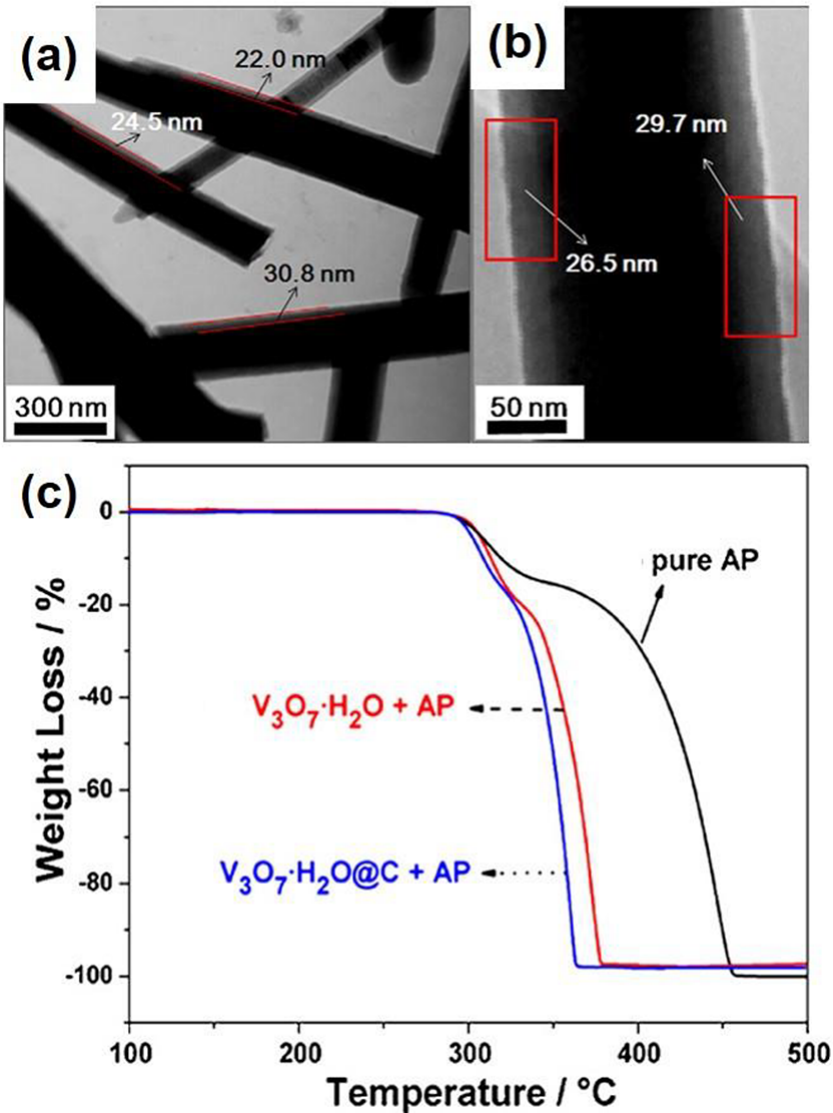
(a) and (b) TEM images of V_3_O_7_·H_2_O@C core–shell structures, (c) TGA curves of pure ammonium perchlorate, V_3_O_7_·H_2_O with ammonium perchlorate and V_3_O_7_·H_2_O@C with ammonium perchlorate. Reproduced with permission from ref (124). Copyright 2011 Elsevier.

Ammonium perchlorate, a common oxidizer, plays a key role in the combustion of composite solid propellants. Furthermore, the catalyst greatly affected the performance of composite solid propellants by the thermal decomposition of ammonium perchlorate.^122,123^ Huang et al.^124^ found that the thermal decomposition temperatures of ammonium perchlorate in the presence of V_3_O_7_·H_2_O nanobelts and V_3_O_7_·H_2_O@C core–shell structures can be dramatically reduced. Both V_3_O_7_·H_2_O nanobelts and V_3_O_7_·H_2_O@C core–shell structures were synthesized by a hydrothermal method. Especially, the core–shell structures are synthesized by using V_3_O_7_·H_2_O nanobelts as the cores and glucose as the source of carbon. A well-defined nanobelt morphology with a length up to several micrometers can be observed (Figure 14a), which consists of a V_3_O_7_·H_2_O core and carbon shell (Figure 14b). The thermogravimetric analysis (TGA) indicated that the addition of V_3_O_7_·H_2_O or V_3_O_7_·H_2_O@C in ammonium perchlorate exhibited a significant reduction in the decomposition temperature of ammonium perchlorate (Figure 14c). The thermal decomposition temperature was lowered by 70 and 89 °C by adding V_3_O_7_·H_2_O or V_3_O_7_·H_2_O@C, respectively. The V_3_O_7_·H_2_O@C core–shell structures exhibited a higher catalytic activity than V_3_O_7_·H_2_O, with two possible mechanisms proposed. First, the partially filled 3d orbit in the vanadium atom promoted the electrotransfer process by accepting electrons from ammonium perchlorate and further accelerated the thermal decomposition of ammonium perchlorate. Second, the amorphous carbon shell possessed lots of active groups (such as C=C, C=O), which could facilitate the thermal decomposition of ammonium perchlorate.

#### 5.2.3 Supercapacitors

**Figure 15:**
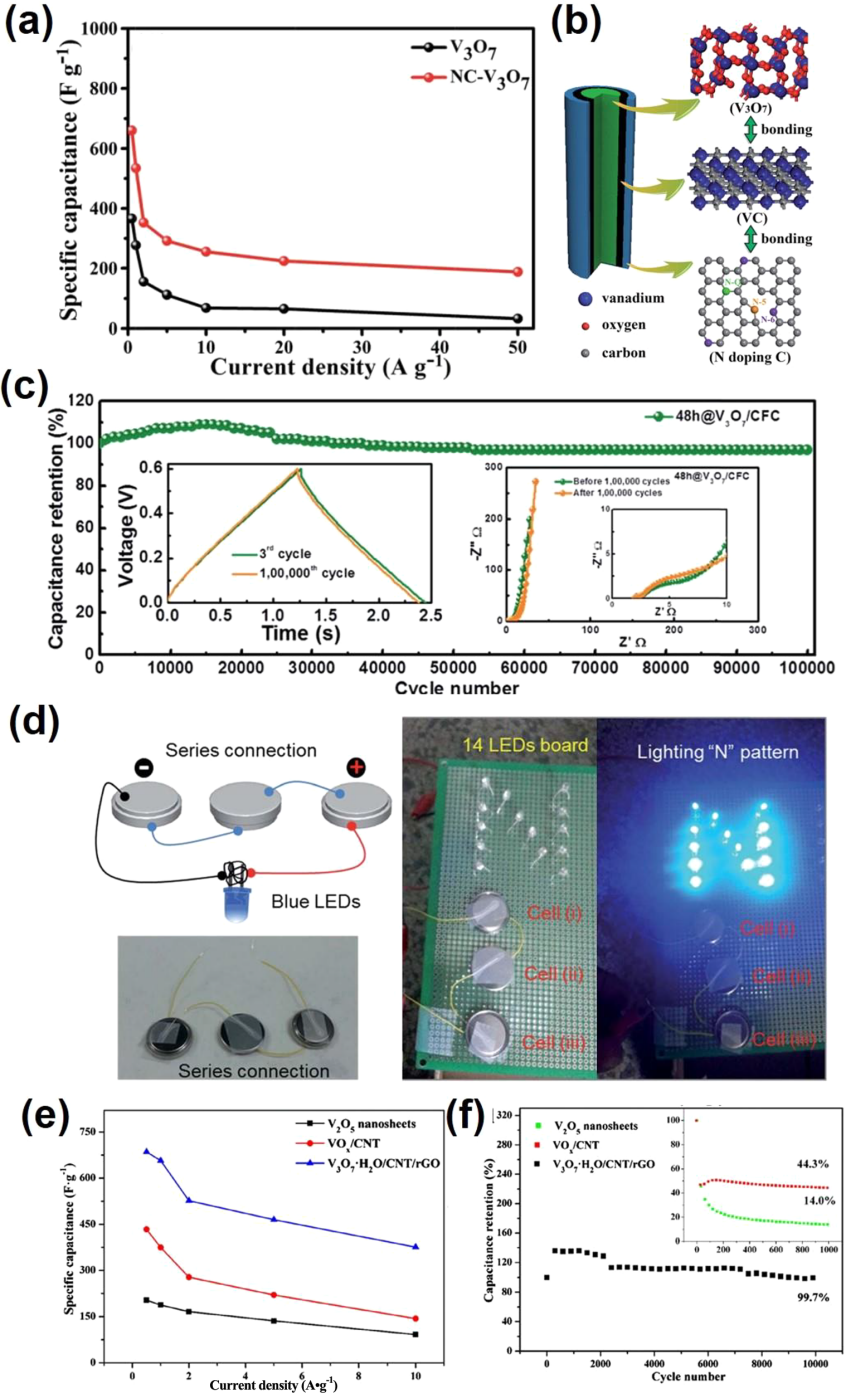
(a) Rate capability of V_3_O_7_ and N-doped carbon coated nest-like V_3_O_7_ calculated from the charge/discharge curves as a function of current density, (b) schematic diagram of the crystal structure and bonding in N-doped carbon coated nest-like V_3_O_7_. Reproduced with permission from ref (112). Copyright 2018 Royal Society of Chemistry. (c) Cycling stability of V_3_O_7_- carbon fiber cloth symmetric supercapacitors devices at a constant current density of 10 A g^–1^ for 100 000 cycles, (d) series connection of three-coin cells for practical applications, charging process of serially connected coin cells, and a demonstration of LEDs lit with charged coin cells. Reproduced with permission from ref (125). Copyright 2018 Royal Society of Chemistry. (e) Comparison of specific capacitance at different current densities from GCD curves, (f) cycling performance at 100 mV s^–1^ of V_2_O_5_ nanosheets, VO_*x*_/CNT, and V_3_O_7_·H_2_O/CNT/rGO. Reproduced with permission from ref (126). Copyright 2018 Elsevier.

V_3_O_7_ and V_3_O_7_·H_2_O are promising supercapacitor materials due to their layered structure and mixed oxidation states of +4 and +5. V_3_O_7_ can be converted to V_6_O_13_ at the lowest potential of −0.6 V and V_2_O_5_ at the highest potential of 0.2 V. Thus, the working potential window is in the range of −0.6 and 0.2 V. Huang et al.^112^ fabricated porous V_3_O_7_ nanowire self-assembled nest-like V_3_O_7_ and investigated the supercapacitor properties. The pure nest-like V_3_O_7_ exhibits worse supercapacitor performance compared with N-doped carbon coated nest-like V_3_O_7_ composites. The N-doped carbon coated nest-like V_3_O_7_ electrode showed a higher specific capacity of 660.63 F g^–1^ at 0.5 A g^–1^ compared to V_3_O_7_ (362.63 F g^–1^). Even at a higher current density of 50 A g^–1^, the N-doped carbon - V_3_O_7_ electrode still exhibits a better performance (187.72 F g^–1^) than V_3_O_7_ (33.18 F g^–1^), as shown in Figure 15a. Furthermore, the N-doped carbon coated nest-like V_3_O_7_ electrode also delivers better stability (80.47% capacitance retention after 4000 cycles) compared with pure V_3_O_7_ (23.16% capacitance retention after 4000 cycles)_._ The superior performance of N-doped carbon coated nest-like V_3_O_7_ is mainly due to the unique three-layer structure: V_3_O_7_ core/carbon/nitrogen doped carbon (Figure 15b). Such a unique three-layer structure can not only stabilize V_3_O_7_, but also provide high-speed ionic and electronic transmission channels, which is responsible for the good supercapacitor performance of N-doped carbon coated nest-like V_3_O_7_. Yu et al.^125^ reported the growth of V_3_O_7_ nanowires on a carbon fiber cloth through a hydrothermal method. The obtained V_3_O_7_/carbon fiber cloth composites show a spider web-like morphology, which exhibits robust adhesion. The composite electrode gives a maximum specific capacitance of 151 F g^–1^ at a current density of 1 A g^–1^ with ultrahigh cycling stability of 97% (after 100000 cycles) in a full cell configuration (Figure 15c). Meanwhile, the V_3_O_7_/carbon fiber cloth composites reveal maximum power and energy densities of 5.128 kW kg^–1^ and 24.7 Wh kg^–1^, respectively by using 1-ethyl-3-methylimidazolium trifluoromethanesulfonate as the electrolyte. Furthermore, coin cell-type configuration with the V_3_O_7_- carbon fiber cloth composites electrode was assembled. The symmetric supercapacitors successfully and effectively power light-emitting diodes to produce blue light (Figure 15d). Huang et al.^115^ reported that V_3_O_7_·H_2_O nanobelts exhibited a capacitance of 447.6 F g^–1^. However, the cycling performance is limited by the poor conductivity and high solubility in an aqueous electrolyte. Therefore, composing with another conductive phase could be an alternative way to fabricate high-performance V_3_O_7_·H_2_O based materials. When the V_3_O_7_·H_2_O nanobelt is incorporated with carbon nanotube and reduced graphene, the formed 3D hierarchical porous composites exhibit outstanding electrochemical performance with a high specific capacitance (685 F g^–1^ at 0.5 A g^–1^) and excellent cycle stability (99.7% after 10,000 cycles) (Figure 15e,f).^126^ Meanwhile, the composites also give relatively high energy densities and power densities of 34.3 Wh kg^–1^ and 150 W kg^–1^, respectively. The better electrochemical performance can be attributed not only to the highly conductive carbon materials, but also to the 3D hierarchical porous structure. The carbon materials offer the transport pathway bridges, leading to the rapid transfer of charges. Meanwhile, the 3D porous structure minimizes the diffusion distance and supplies a large surface area with abundant active sites.

In general, vanadium oxides have received massive interest as supercapacitor electrodes that exhibit high theoretical specific capacity than most of the other transition metal oxides due to their variable valence state from V^2+^ to V^5+^. In addition, the layered structure of vanadium oxides facilitates the intercalation/deintercalation of electrolyte ions during the charging/discharging process. However, vanadium oxides-based supercapacitor electrode materials still suffer from poor long-term cycling stability, which is usually caused by the collapse of a layered crystal structure, severe agglomeration of particles, and low electrical conductivity. The electrochemical stability of vanadium oxide-based supercapacitors can be improved by material modification, optimization of the structure, or combining with other materials with excellent electrical conductivity. Developing vanadium oxide nanomaterials with suitable micro-/nanostructures is an important factor to improve the cycling stability. 3D vanadium oxides with micro-/nanostructures are often employed, including microspheres and hollow spheres, which can inherit the superior high surface area characteristics of nanobuilding blocks, and simultaneously possess a decent structural stability.^127−130^ Furthermore, the 3D structure can effectively reduce the agglomeration of particles, which is beneficial for the cycling and rate performance.^131^ Integrating vanadium oxides with carbon materials has been demonstrated to be effective to suppress the structural degradation upon cycling. The carbon materials, such as porous carbons and graphene, can serve as elastic buffering layers to release the strain within metal oxides during cycling.^132^ To some extent, carbon materials can avoid loose attachment between the electrode material and current collector, which helps improve both the conductivity and the stability of the supercapacitor.

#### 5.2.4 Electrochromism

**Figure 16:**
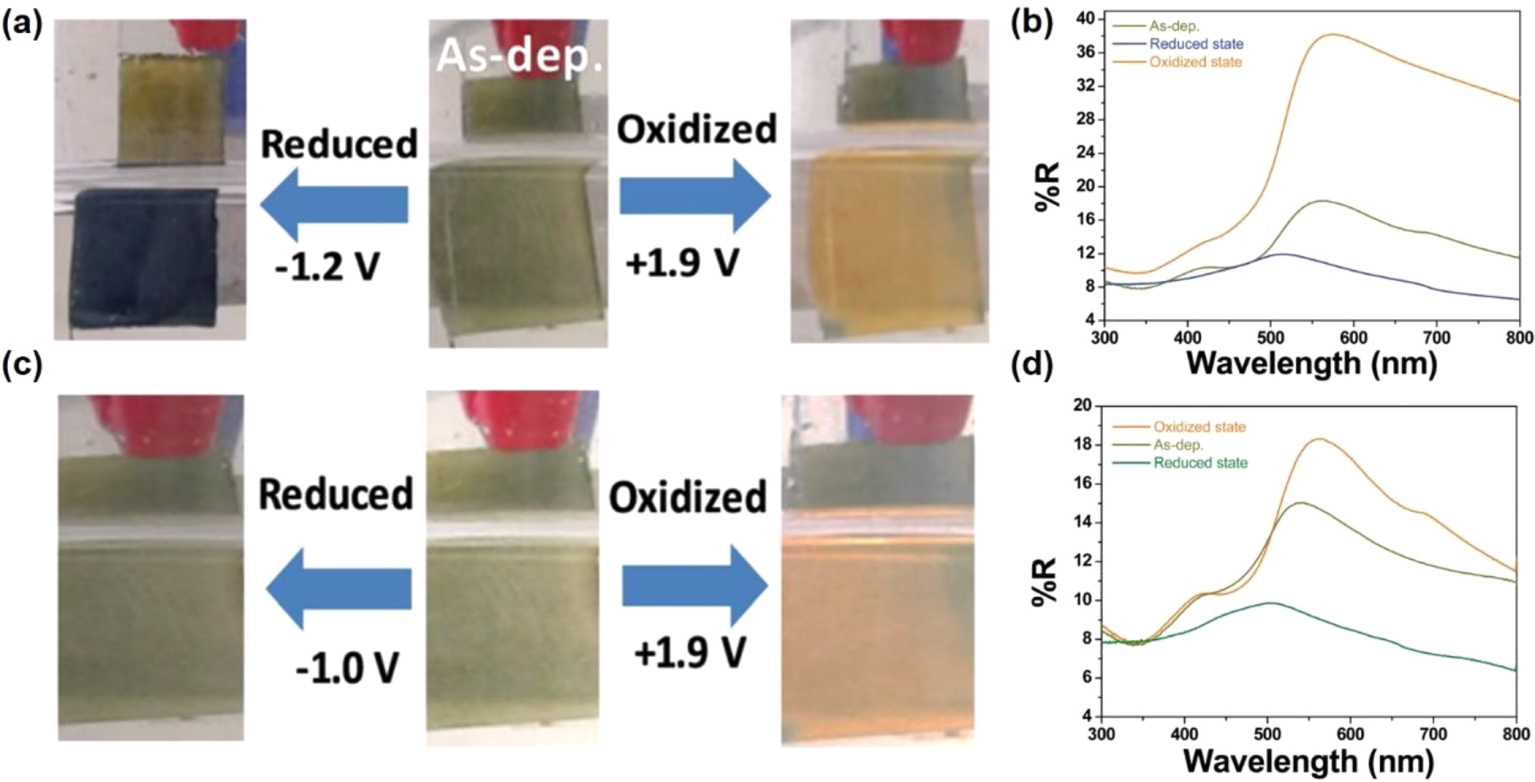
(a) The visual appearance of V_3_O_7_·H_2_O films in the reduced and oxidized states in LiTFSI, (b) diffuse reflectance spectra for the V_3_O_7_·H_2_O films cycled in Li-electrolyte/Pt vs SCE, in reduction at −1.2 V and reoxidized at +1.9 V, (c) the visual appearance of V_3_O_7_·H_2_O films in the reduced and oxidized states in NaTFSI, (d) diffuse reflectance spectra for the V_3_O_7_·H_2_O films cycled in Na-electrolyte/Pt vs SCE, in reduction at −1 V and reoxidized at +1.9 V. Reproduced with permission from ref (138). Copyright 2020 Royal Society of Chemistry.

Nanostructured V_3_O_7_ thin films showed electrochromic properties by using lithium perchlorate as the electrolyte, which was prepared by a nebulizer spray pyrolysis technique.^133^ The color of the films is changed from yellow to pale blue by applying an external potential of 1.5 V for intercalation of Li^+^ ions, while the color is reversed by applying an external potential of −1.5 V for deintercalation of Li^+^ ions. Such results indicate that the nanostructured V_3_O_7_ thin films could be effectively used for smart window applications. In general, several models are proposed to explain the electrochromic phenomenon, such as the color center model (Deb model), electrochemical redox model, and so on.^134^ The “Deb’s color center model” is in nature related to the defects (such as oxygen vacancies) induced change of the visible light absorption, which is generally independent of the external electric field,^135^ while in the “electrochemical redox model” it is believed that the injection and trapping of a large density of electron and hole lead to coloration.^136^ The mechanism of electrochromism for V_3_O_7_ thin films is the variation of the band structure caused by intercalation/deintercalation of Li^+^ ions, which belongs to the electrochemical redox model. During the application of negative bias, Li^+^ ions are absorbed onto the surface of V_3_O_7_ and diffuse into the lattice of V_3_O_7_. The intercalated Li^+^ ions react with O^2–^ ions to introduce oxygen vacancies in the lattice and reduce the V^5+^ in the mixed state V_3_O_7_ to V^4+^. As the result, the optical transparency of the film changes.^137^ Moreover, the intercalation of Li^+^ ions upshifts the Fermi level close to the conduction band, which leads to an increased transmittance of V_3_O_7_. The process can be reversed by deintercalating of Li^+^ ions through applying positive bias. Furthermore, the V_3_O_7_·H_2_O thick films also show good electrochromic properties (good reversibility and good color switching between reduced and oxidized states).^138^ By using lithium bis-trifluoromethanesulfonimide (LiTFSI) as the electrolyte, the color of the V_3_O_7_·H_2_O films changes from green to orange by applying a positive potential of 1.9 V, while it changes from green to blue by applying a negative potential of −1.2 V (Figure 16a). Such film presented a maximum optical reflectance modulation of 29% at 590 nm (Figure 16b). The V_3_O_7_·H_2_O films still exhibited color changes by using a Na-based electrolyte (Figure 16c) and a maximum optical reflectance modulation of 10% at 590 nm (Figure 16d). The maximum optical reflectance modulation of Na-based electrolyte is lower than that in the Li-based electrolytes, which is mainly due to a larger faradaic contribution resulting from the larger cation size of Na^+^ ions.

## 6 VO_2_

### 6.1 Structures and Synthesis

**Figure 17:**
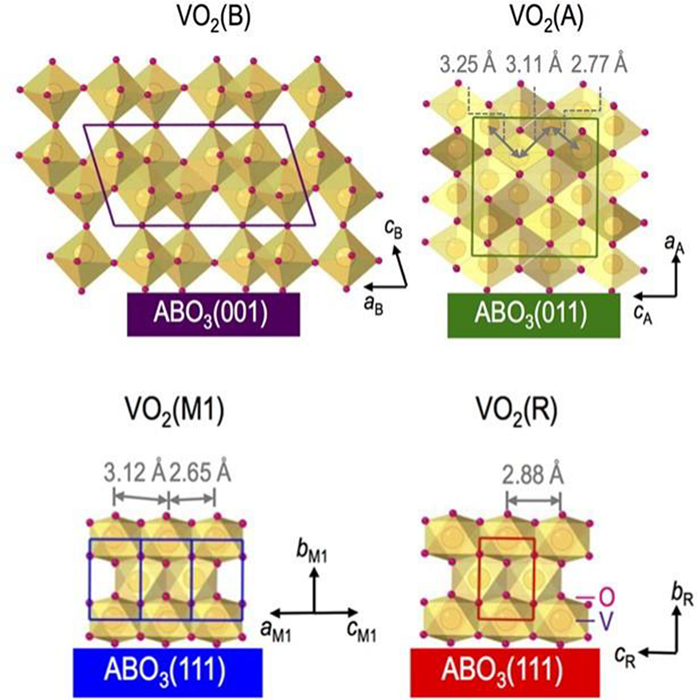
Different phases of VO_2_ with respective lattice parameters. Reproduced with permission from ref (147). Copyright 2016 under CC BY 4.0 license.

Vanadium dioxide (VO_2_) can exist in various polymorphic phases, including but not limited to VO_2_ (B), VO_2_ (A), VO_2_ (M), VO_2_ (R), VO_2_ (D), and VO_2_ (P).^139^ Some of these phases and their corresponding lattice parameters are shown in Figure 17. The discussion will focus more on VO_2_ (R) and its reversible MIT to VO_2_ (M) as well as on VO_2_ (B) due to its application as cathode materials in electrochemical devices.

VO_2_ was first demonstrated to undergo MIT in 1959 by Morin. At the critical temperature (*τ*_C_) of about 340 K, VO_2_ transforms from high-temperature, conducting rutile VO_2_ (R) to low-temperature, insulating monoclinic VO_2_ (M). There are two mechanisms that have been used to describe this ultrafast phase transition phenomenon in VO_2_, namely the Peierls model and Mott-Hubbard model. The Peierls model describes the nature of MIT as the change from a shared d-orbital between all vanadium atoms to localized d-orbital in the V–V dimer, which is a result of the change in the V–V distance from 2.88 Å in VO_2_ (R) to 2.65 and 3.12 Å in VO_2_ (M).^20^ Therefore, the Peierls model stated that the structural distortion is the cause of MIT. Wentzcovitch et al. applied the local-density approximation to study the electronic and structural change of VO_2_ during the MIT.^140^ From the view of band theory, a monoclinic distorted state is in good agreement with the experiment result. Meanwhile, the structural distortion enhances the bonding between neighboring V atoms, which is expected in the Peierls model. Moreover, Baum et al. utilized four-dimensional (4D) femtosecond electron diffraction to visualize the phase transformation process of VO_2_.^141^ They pointed out that during the MIT, the displacement of atoms happened within picoseconds, and followed by the sound wave shear motion of the crystal in the time scale of nanoseconds. The observation indicates the occurrence of fast structural distortion during the MIT.

On the other hand, the Mott-Hubbard model states that MIT would occur when the electron density (*n*_e_) and Bohr radius (*a*_H_) satisfy *n*_e_^1/3^*a*_H_ ≈ 0.2.^142^ Compared with the Peierls model, the Mott-Hubbard model has the advantage in explaining the phenomenon such as the anomalously low conductivity in the metallic phase.^143^ Whittaker et al. summarized multiple experiment cases and pointed out that the metallic phase of VO_2_ might be introduced without the structural phase transformation if the excitation of carriers reaches a threshold density.^144^ Their observation provides key evidence in revealing the nature of MIT. While there remains a debate on which mechanism best describes the MIT, the usage of both models is strongly encouraged due to the transition kinetics^145^ as well as the stimuli involved during the transition. Shao et al.^146^ reported recent progress in understanding the mechanism and kinetics of MIT, including the lattice distortion and electron correlations (Peierls phase transition, Mott phase transition) and modulation methods (elemental doping, external electric field, light irradiation, and strain engineering).

Upon application of suitable external stimuli (i.e., photons, heat, electric, magnetic, electrochemical, and stress) to initiate the MIT, physical properties of VO_2_, such as electrical resistance, optical transmittance, and thermal conductivity, are reversibly and drastically changed. Long et al.^27^ have summarized the connections between the stimuli and responses of VO_2_-based devices in detail. This flexibility in external stimuli and corresponding responses is the main reason why VO_2_ is a material of interest in multiple novel devices spanning from thermally,^139,148^ electrically,^149−151^ to optically^152,153^ activated devices. The functional performance of these devices is thus highly dependent on, but not limited to, the physical attributes, such as dimension, morphology, doping level, and crystallinity, of the fabricated VO_2_. Multiple techniques have been used to fabricate functional VO_2_ devices, each with their own strengths and weaknesses. Some of these fabrication processes are reviewed with polymer-assisted deposition methods,^154^ hydrothermal,^139^ sol–gel,^155^ chemical vapor deposition (CVD),^19^ and physical vapor deposition (PVD)^156^ methods discussed in detail regarding the controlled synthesis of VO_2_ for thermochromic application.

### 6.2 Applications

In recent years, multiple omnibus reviews^157−159^ have been done in attempts to give the most encompassed view of VO_2_ research progress, often including a combination of the following topics: MIT mechanism, kinetics, fabrication techniques, and applications. While these reviews could offer wide coverage of VO_2_ research progress in multiple topics, in-depth discussion of each topic, applications in this case, is in much needed demand. In subsequent sections, reported applications of VO_2_ in functional devices both based and not based on MIT in recent years, especially in the last three years, are classified and discussed in different categories: optical, electrical, and mechanical applications.

#### 6.2.1 Optical Applications

**Figure 18:**
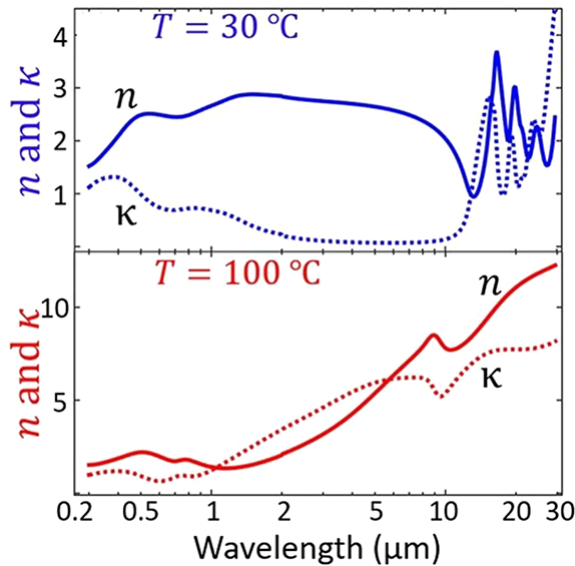
Optical constants *n* and *k* of VO_2_ film with respect to the wavelength and the temperature changes. Reproduced with permission from ref (160). Copyright 2019 John Wiley and Sons.

The operations of these VO_2_-based optical devices are often based on the changes in refractive index, *n*, and extinction coefficient, *k* of VO_2_ upon crossing the MIT. The changes to *n* and *k* at different temperatures are shown in Figure 18. These optical functional devices are further divided into two main groups: infrared regulators and optical switches.

##### 6.2.1.1 Infrared Regulators

**Figure 19:**
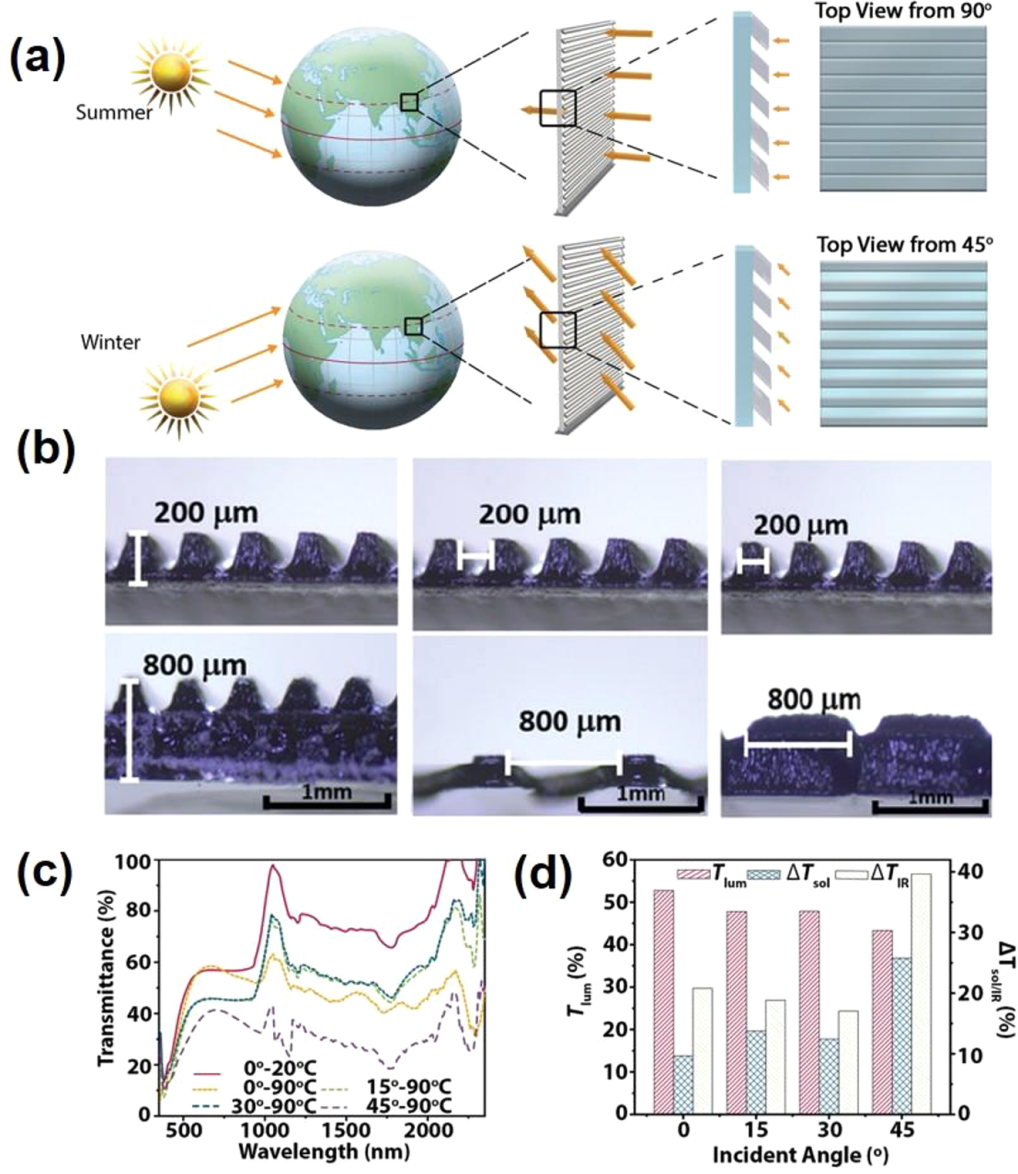
(a) Schematic design of the 3D printed smart windows design. (b) Optical microscopic pictures of the printed composite structures with identical 0° tilted angles with a range of thickness, spacing, and width. (c) UV–vis–NIR transmittance spectrum of the printed composite structure with respect to different incident angles and temperature. (d) *T*_lum_, Δ*T*_sol_, and Δ*T*_IR_ diagram of the printed composite structures with different incident angles. Reproduced with permission from ref (173). Copyright 2020 John Wiley and Sons.

Since its first introduction in 1985,^161^ the smart window has attracted much attention due to rising energy consumption in the commercial building sector, which contributes up to 40% of total consumption globally and leads to 30% of global greenhouse gas emissions today.^162^ VO_2_ is the prime candidate for smart window materials due to its ability to seamlessly and rapidly regulate the amount of infrared (IR) across MIT with miniscule side effects to the visible transmission. However, intrinsic limitations (high τ_C_ ≈ 68 °C, low luminous transmittance (*T*_lum_) < 40%, poor solar modulation (Δ*T*_sol_) < 10%, and poor durability) prevent pristine VO_2_ from meeting the requirements for commercial smart window applications. The most common way to reduce *τ*_C_ is by elemental (i.e., W, Mo, Ti, F, Mg, etc.) doping as summarized by Cui et al.^163^ Different approaches exist to enhance *T*_lum_ and Δ*T*_sol_ via advanced device morphological engineering such as multilayered VO_2_,^164^ biomimetic structure,^165−168^ nanothermochromism,^169^ porous,^170^ and gridded structure.^171−173^ Zhou et al. recently reported a new customized VO_2_ composite structure in which a new factor, the incident angle, was considered in the development of smart window devices. Figure 19a shows a schematic of how different incident angles in the summer and winter can be taken advantage of with the reported customizable composite structure. The VO_2_ composite structure was fabricated through a 3D printing procedure, enabling the flexibility of device dimensions. Several samples of different thicknesses, widths, and spacing are demonstrated in Figure 19b. The incident-angle dependency properties of this structure are shown in Figure 19c,d in which modulating performance of the 3D printed device improves significantly, and Δ*T*_sol_ improves from 9.7% to 25.8% with respect to an increase in the incident angle from 0° to 45°.^173^

**Figure 20:**
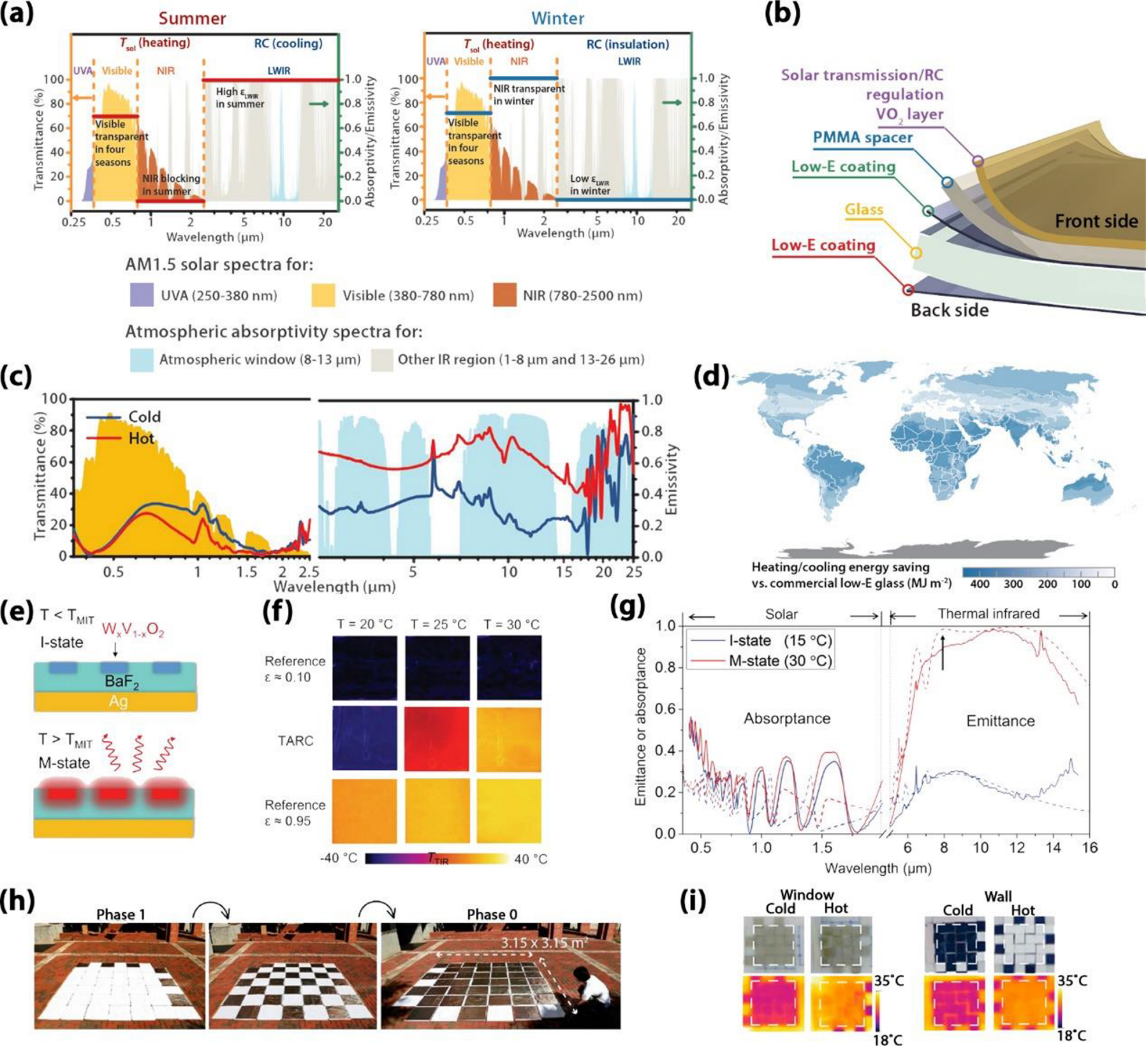
(a) Concept of the ideal energy-saving smart window. The red and blue lines represent the spectra for an ideal energy-saving smart window in the summer and winter. (b) Schematic structure of the RCRT window. (c) Spectra of the sample with RC regulation in the visible-NIR and LWIR range at 20 °C (blue line) and 90 °C (red line) against a normalized AM1.5 global solar spectrum (yellow shadow) and LWIR atmospheric transmittance window (blue shadow). (d) Estimated world heating and cooling energy-savings of a W-doped max Δε sample against a commercial low-E glass as the baseline. Reproduced with permission from ref (174). Copyright 2021 American Association for the Advancement of Science. (e) Schematics of materials composition and working mechanism of the TARC. (f) IR images of TARC compared with those of two conventional materials (references) with constantly low or high thermal emittance showing the temperature-adaptive switching in the thermal emittance of TARC. (g) Solar spectral absorptance and part of the thermal spectral emittance of TARC at a low temperature and a high temperature. Measurements (solid curves) show consistency with theoretical predictions (dashed curves). Reproduced with permission from ref (175). Copyright 2021 American Association for the Advancement of Science. (h) Demonstration of the surface transition in a meter-scale Al-paper sample. (i) Photographs and corresponding thermal images of the ITO/VO_2_/PVC sample (“window” in the figure) and the ITO/black paint/PVDF-HFP sample (“wall” in the figure) on the two phases. Effective areas are marked by the dashed lines. Reproduced with permission from ref (176). Copyright 2022 American Chemical Society.

Aside from NIR transmittance regulation in smart windows, VO_2_ also has an unusual ability to change its long-wave infrared emissivity (ε_LWIR_) upon crossing the MIT. An ideal smart window should have high transparency in the visible region (380–780 nm), while having a transparent state in the winter and an opaque state in the summer (Figure 20a). Moreover, the ideal smart window should have a high ε_LWIR_ at a high temperature to promote radiative cooling (RC) and a low ε_LWIR_ at a low temperature to suppress RC. Based on this concept, Long et al.^174^ fabricated a VO_2_-based multilayer structure which was able to regulate NIR transmittance and ε_LWIR_ spontaneously (Figure 20b). Through forming a Fabry–Perot resonator, the passive RC regulating thermochromic (RCRT) smart window possessed an ε_LWIR_ of 0.21 at 20 °C, while the ε_LWIR_ increased to 0.61 above *τ*_c_. In addition, the RCRT window kept a promising *T*_lum_ of 27.8% and a Δ*T*_sol_ of 9.3% (Figure 20c). With the actual building energy consumption simulation conducted with a 12-story building, the RCRT window yielded a higher energy savings compared with a commercial low-E window across different climate zones (Figure 20d). Meantime, Wu et al.^175^ designed a flexible temperature-adaptive radiative coating (TARC) through embedding lithographically patterned W-doped VO_2_ in a dielectric BaF_2_ layer on top of a reflective gold layer (Figure 20e). The TARC film had a low ε_LWIR_ (∼0.2) in the insulation state and a high ε_LWIR_ (∼0.9) at the metallic state (Figure 20f), and the observation agreed with the simulation (Figure 20g). Long et al.^176^ further expanded the concept of RC regulation from window to wall by preparing a switchable interwoven structure. As shown in Figure 20h, through pulling the block of interwoven structure, the original exposed block on the top side becomes concealed, and the underneath block becomes exposed. As a result, the structure switches its phase from phase 0 to phase 1. Taking into account different requirements of windows and walls, Long’s group designed on-demand interwoven structures. Figure 20i shows the interwoven structure for window and wall applications. As discussed in Figure 20a, a window requires high visible transmittance and dual-band regulation for NIR and LWIR ranges. An ITO/VO_2_/PVC combination was employed for windows. In this structure, VO_2_ was used to regulate NIR, while the ε_LWIR_ was regulated by alternatively exposing low-E ITO and high-E PVC. On the other hand, an ideal wall has high solar absorption and low ε_LWIR_ in the winter, and low solar absorption and high ε_LWIR_ in the summer. An ITO/black paint/PVDF-HFP combination was utilized to cater to this demand. On cold days, visible transparent ITO is exposed, and sunlight will be absorbed by black paint underneath, while on hot days, the highly solar reflective high-E PVDF-HFP is exposed to prompt RC. Hence, compared with a conventional performance index *T*_lum_ and Δ*T*_sol_, the newly proposed ε_LWIR_ needs to be included to gauge the real energy-saving performance.^177^ Moreover, VO_2_ is notoriously known for poor durability, which is the bottleneck for the applications in smart windows. There are two recent reports to embed VO_2_ in the V_2_O_5_ matrix, and such a strategy could increase the lifetime up to 33 years.^178,179^

**Figure 21:**
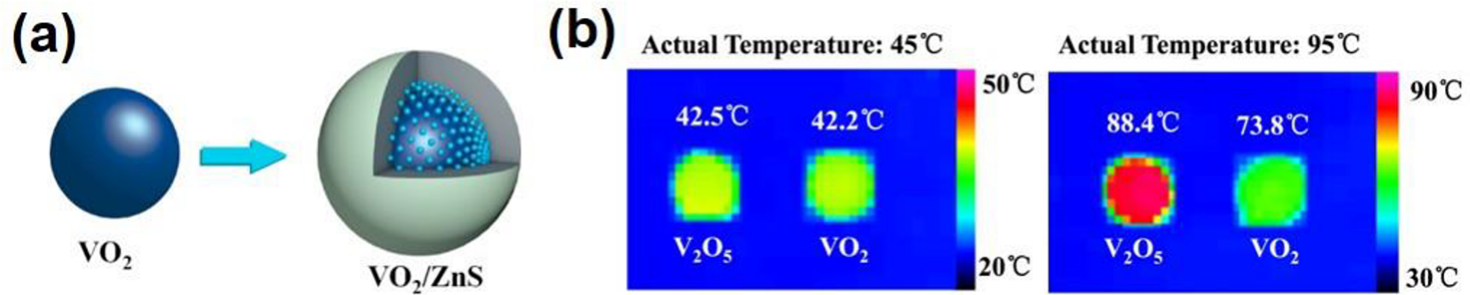
(a) Schematic of the VO_2_/ZnS core–shell nanopowder design. (b) The infrared thermal images of same actual temperature for VO_2_/ZnS core–shell nanopowder and reference V_2_O_5_ pellets at 45 °C (upper) and 90 °C (lower). Reproduced with permission from ref (182). Copyright 2018 Elsevier.

Besides the application in building, the unique property of emissivity switching makes VO_2_ the material of interest for IR camouflage and passive radiator for military and aerospace applications because VO_2_-based devices can function entirely on the thermal trigger with no additional sources, electrical or otherwise, required. VO_2_-based camouflage devices work by reducing the amount of IR emitted into the environment, shrouding the user from being detected with an IR detector such as most night-vision technologies. Examples include VO_2_/graphene/CNT heterostructure by Xiao et al.,^180^ VO_2_/carbon hybrid by Wang et al.,^181^ and VO_2_/ZnS core–shell structure (Figure 21a) by Ji et al.^182^ As seen in Figure 21b, under a similar IR detector and temperature, the VO_2_/ZnS core–shell pallets exhibit the ability to control their IR radiation intensity and lower their detected temperature as compared to V_2_O_5_ pallets with constant emissivity.

**Figure 22:**
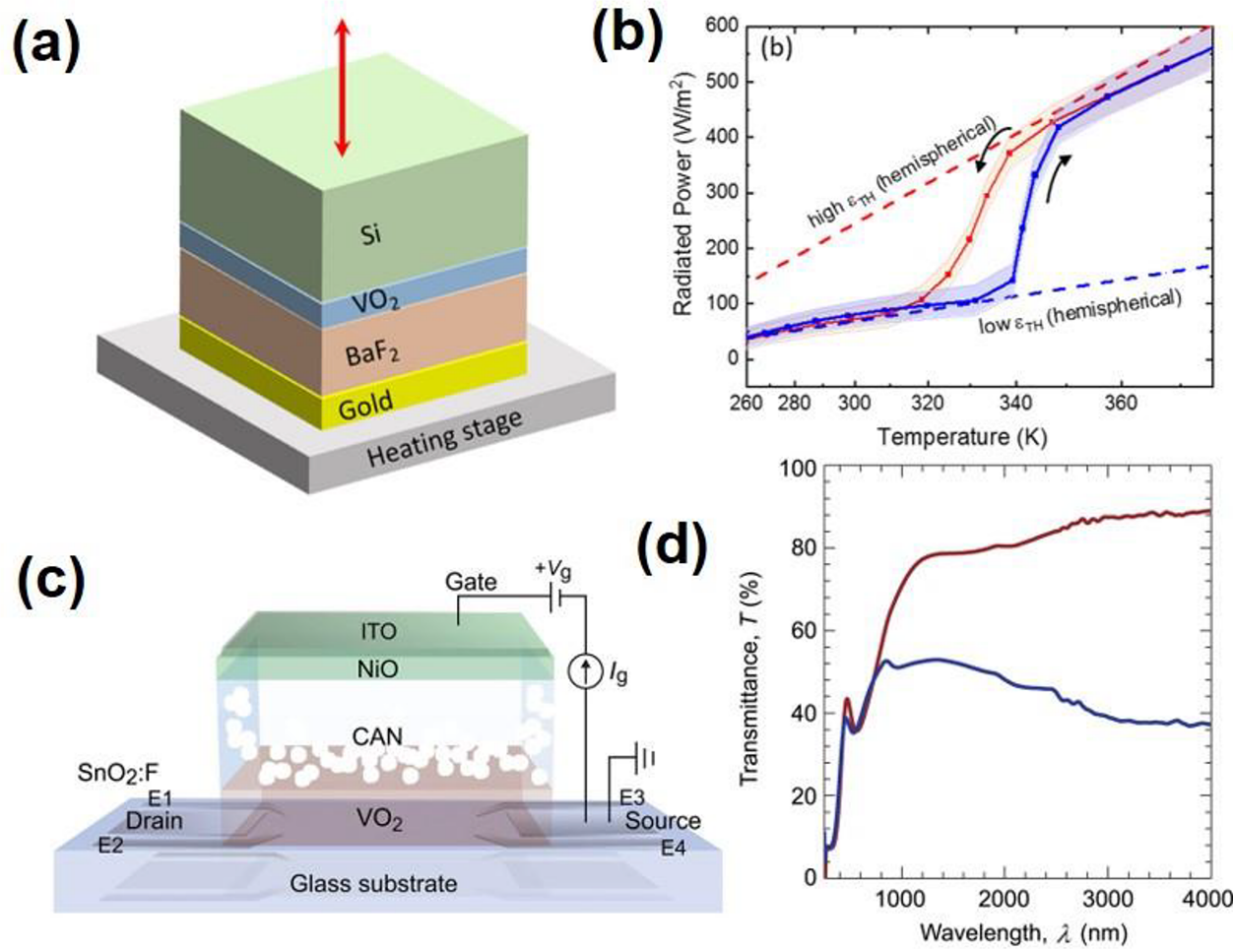
(a) Schematic of the Si/VO_2_/BaF_2_/Au passive radiator design. (b) Radiated thermal power of the Si/VO_2_/BaF_2_/Au device, with heating and cooling indicated with arrows; the dotted line is the simulation data for VO_2_ (M) and VO_2_ (R) respectively. Reproduced with permission from ref (184). Copyright 2019 under CC BY 4.0 license. (c) Schematic of the three-terminal transistor design. (d) Optical transmittance spectra measured before (red line) and after (blue line) applying *V* = +12 V. Reproduced with permission from ref (186). Copyright 2017 under CC BY license.

Different from a camouflage device, a VO_2_-based passive radiator requires modification to the device structure to counter the lower emissivity at the higher temperature. This intrinsic problem could be overcome by depositing VO_2_ on a highly reflective metal substrate with^183,184^ or without^185^ a spacer layer. Figure 22a is a multilayer Si/VO_2_/BaF_2_/Au structure reported by Kim et al.,^184^ which was designed and tested specifically for simulated space (vacuum) applications. Figure 22b demonstrates the radiated thermal power of the multilayer device. Experimental data were compared with simulated ones for both high and low-temperature operations. The measured radiated power at 300 and 373 K was 72 W/m^2^ and 552 W/m^2^, respectively, showing a massive jump in emitted radiation upon crossing the MIT threshold.^184^ Due to the typical multilayer design of a VO_2_-based passive radiator, factors such as functional emissivity difference between low and high temperature as well as the wavelength of emitted light can be further fine-tuned by adjusting the substrate/spacer/film combination. The electrochromic setup has also been shown to also result in IR regulating behavior in VO_2_-based devices.^186^Figure 22c is the schematic of a three-terminal thin-film-transistor-type electrochromic device by Katase et al.^186^ Upon application of external voltage (+12 V according to literature), the VO_2_ channel undergoes protonation, and the device becomes IR opaque, similar to smart window applications. When a reversed voltage is applied, deprotonation happens, and the device becomes IR transparent once again. Figure 22d shows optical transmittance spectra measured during this transition with +12 V stimulus. The optical transmittance modulation ratio at λ of 3000 nm was 49%.^186^ Electrical input into a VO_2_-based device can also be utilized as a Joule heating source for MIT. To realize this, VO_2_ can be combined with transparent conductive electrode materials such as ITO, Ag NWs, CNT, etc.^187,188^ to become an electro-optic modulator. An example of such a modulator is a VO_2_+Au/GaN/Al_2_O_3_ device by Fan et al.,^189^ which has the ability to change the transmission step-by-step according to the applied voltage.

##### 6.2.1.2 Optical Switches

**Figure 23:**
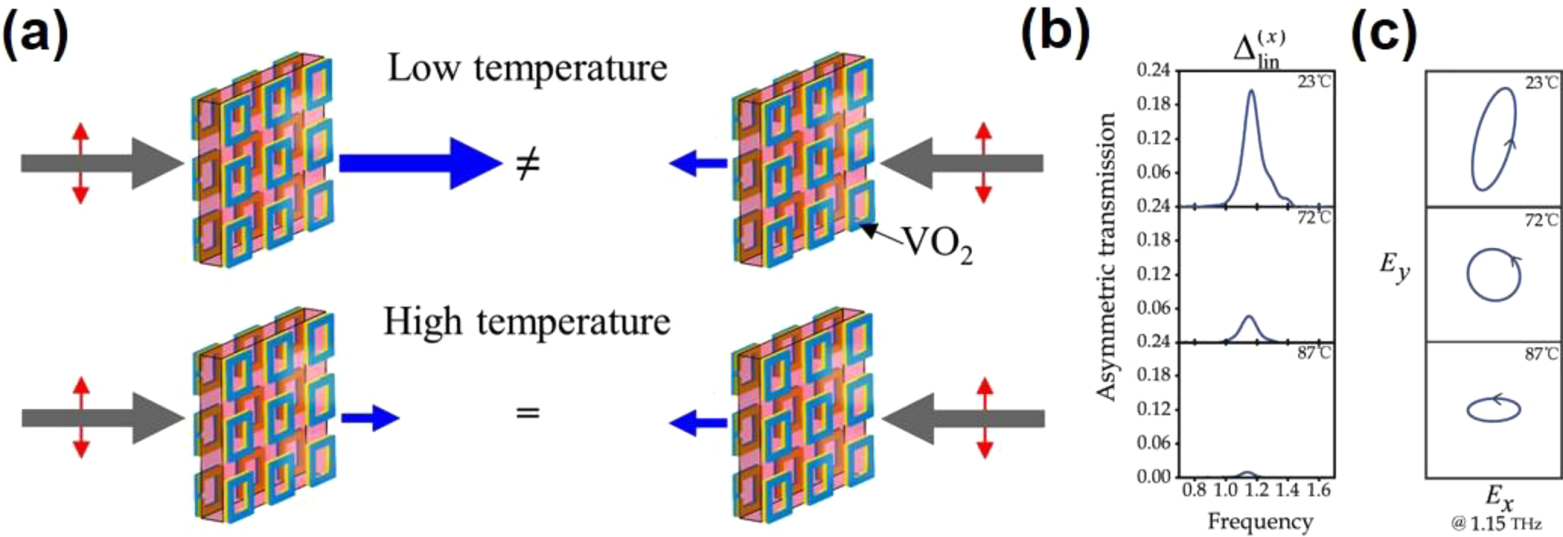
(a) Schematic of the temperature-controlled VO_2_ metamaterial asymmetric optical switch. (b) Frequency dependence of the asymmetric transmission parameter for linearly polarized waves, and (c) transmitted polarization state at 1.15 THz for illumination with x-polarized waves. Reproduced with permission from ref (194). Copyright 2019 under CC BY 4.0 license.

Based on the sudden change in the optical constants *n* and *k* of VO_2_ across MIT, radio frequency (RF) switches or waveguides can be fabricated to control the flow of electromagnetic waves (i.e., microwave and radiowave).^190^ Even though the design of each device is largely dependent on whether it is thermally^191^ or electrically activated,^120^ the mechanism for turning from the ON to OFF state is still entirely based on the transition from VO_2_(M) to VO_2_(R) respectively. Examples of RF-switches are the thermally configurable hybrid Al nanoholes/VO_2_ photonic switch by Sun et al.,^192^ metamaterial design by Ding et al. which can act as an absorber from 0.562 to 1.232 THz at room temperature and a high-efficiency halfwave plate at high temperature,^193^ and temperature controlled asymmetric optical switch by Liu et al.^194^Figure 23a shows the working principal of this design in which different output of the same polarized electromagnetic wave input can be achieved at low temperature by physically reverting the device by 180° while achieving similar output at high temperature. This asymmetrical mechanism is demonstrated in Figure 23b,c. A large asymmetry exceeding 20% was detected at 23 °C, while it disappeared almost entirely at 87 °C (Figure 23b). With incident x-polarized waves, the device gave y-polarized waves output at 23 °C, and this can be considered the ON state. Upon heating to 87 °C, output waves returned to approximately x-polarized, similar to input waves, turning the switch OFF (Figure 23c).^194^ While the thermal- and electrical-activated optical switches mainly depend on the change of optical constant upon MIT, the optical-activated switches of VO_2_ focus on the speed of the transition as the defining factor. However, the details of the ultrafast induced phase transition of VO_2_ are not the focus of this review; it can be found in a summary and discussion by Wegkamp et al.^195^ VO_2_ ability to switch between the insulator and metal state within picoseconds is promising for the field of nanophotonics as well as all-optical integrated circuits (i.e., switches, modulators, and data-storage devices). VO_2_ integrated metamaterials have been reported to exhibit nonlinear transmittance by Liu et al.^153^ in the THz range as well as broadband responses spanning from the visible to mid-infrared range by Guo et al.^196^ VO_2_/Au nanoplate memory device was also reported by Lei et al.,^197^ giving a stepwise tuning ability with the use of successive laser pulses.

##### 6.2.1.3 Plasmonic Applications

**Figure 24:**
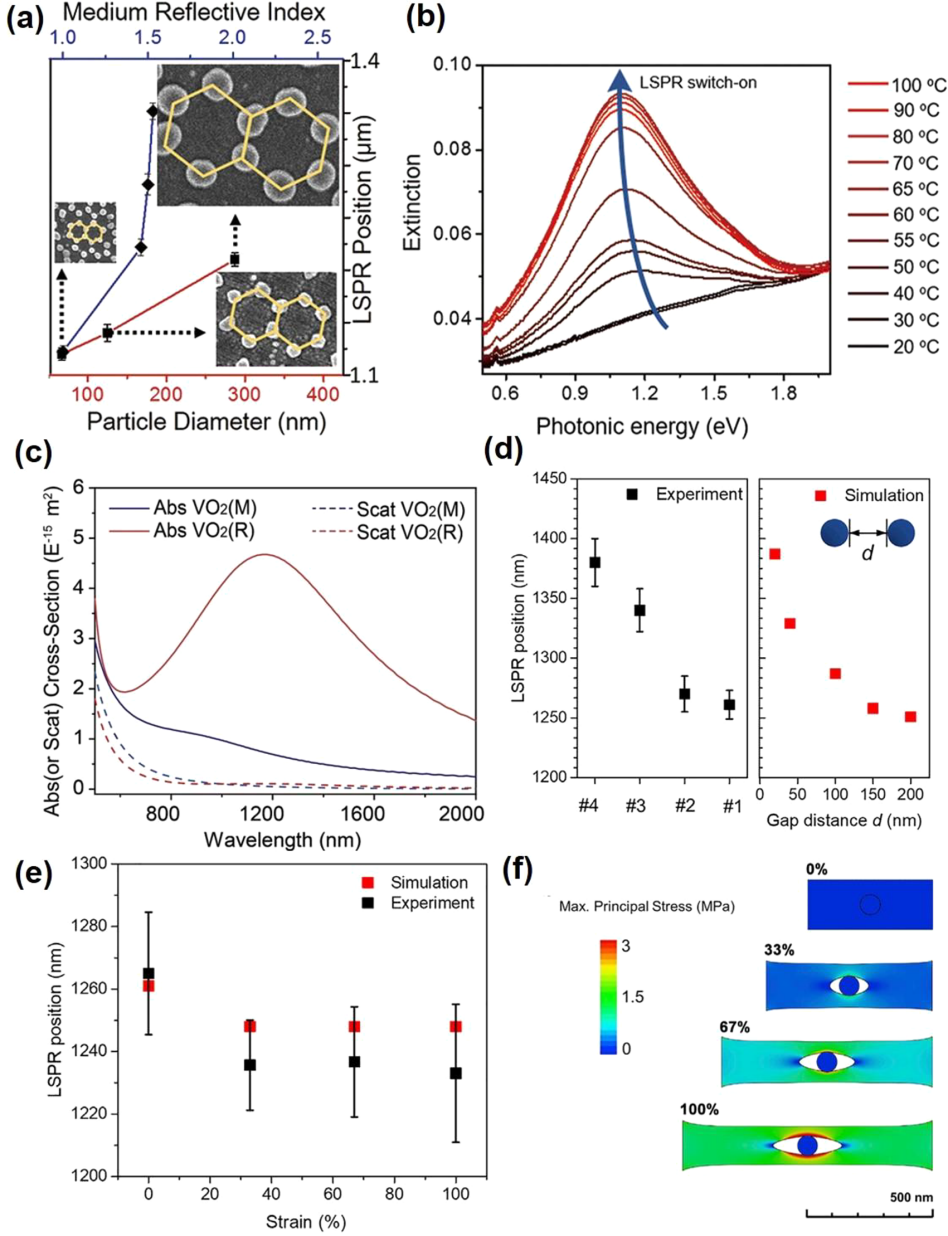
(a) Effects of the particle diameter and medium reflective index to the LSPR position. Insets are the SEM images of VO_2_ NPs with corresponding diameters, and the array structures are highlighted as yellow hexagons. (b) Extinction spectrum of VO_2_ NP under different temperatures from 20 to 100 °C. Reproduced with permission from ref (198). Copyright 2017 American Chemical Society. (c) Simulated absorbance and scattering intensity crossing a VO_2_ NP embedded in PDMS matrix. Reproduced with permission from ref (168). Copyright 2020 Elsevier. (d) Experimental LSPR position of VO_2_ NPs with different dispersion degrees in PDMS, and the simulation result of two adjacent VO_2_ NPs with a different gap. (e) Experimental and simulation results of the LSPR position of VO_2_–PDMS composites under applied strains from 0% to 100%. (f) Simulated stress contours of the representative VO_2_–PDMS composite under applied strain from 0% to 100%. Reproduced with permission from ref (28). Copyright 2019, Cell Press.

The reversible crystal phase transition makes VO_2_ very unique among plasmonic materials. It undergoes a crystal phase transition from the monoclinic semiconductor state to rutile metallic state with significantly promoted conductivity and free carrier density,^27^ leading to a significant difference in its plasmonic property. Recently, VO_2_ nanoparticles (NPs) have been reported with thermal-responsive localized surface plasmonic resonance (LSPR) in the NIR region.^168,198^ Based on the colloidal lithography method,^59^ Long’s group successfully produced the hexagonally patterned VO_2_ NPs on quartz with controllable average diameters from ∼70 to ∼280 nm.^198^ It was observed that the LSPR position of metallic VO_2_ shifts to the longer wavelength on the larger NPs (∼1120 to ∼1220 nm) or under the increasing reflective index of the surrounding medium (∼1120 to ∼1360 nm) (Figure 24a). Besides, the NIR LSPR is temperature-responsive that is quenched on a low-temperature semiconductor state and can be gradually switched on from 20 to 100 °C (Figure 24b). They further investigated the LSPR-induced absorbance and scattering effects of VO_2_ plasmonics through a finite-difference time-domain method.^168^ On a single VO_2_ NP, it is revealed that both the absorbance and scattering are low at the semiconductor state (monoclinic, M), while a strong absorbance emerges in the metallic state (rutile, R) (Figure 24c). This result suggests that the LSPR in metallic VO_2_ is characterized as a strong absorbance enhancement and a relatively weak scattering effect. Moreover, they reported the dispersity- and strain-induced LSPR response on VO_2_ NPs in the polydimethylsiloxane (PDMS) elastomer matrix (Figure 24d,e).^28^ The dispersity-induced LSPR position can be attributed to the changes in average gaps among VO_2_ NPs in the matrix, which is consistent with the simulation result (Figure 24d), while the strain-dependent LSPR position change can be explained by the local reflective index change induced by the delamination between the NP and matrix under applied strains as being demonstrated by the finite element method (Figure 24e,f). A more recent report used a similar approach to tailor the VO_2_ surface plasmon by manipulating its atomic defects and establishing a universal quantitative understanding.^199^ Record high tunability is achieved for LSPR energy from 0.66 to 1.16 eV and a transition temperature range from 40 to 100 °C. The Drude model and DFT calculation reveal that the charge of cations plays a dominant role in the numbers of valence electrons to determine the free electron concentration. It is believed the investigation of VO_2_ LSPR is still in its early stage. The reversible crystal transition makes VO_2_ an intrinsic active plasmonic material, which is unique among the plasmonic field. It is expected for researchers to further understand the VO_2_ plasmonic and to explore its potential applications.

#### 6.2.2 Electrical Applications

Not only optical constants, but the electrical conductivity of VO_2_ is also altered dramatically upon transitioning from insulating VO_2_ (M) to metallic VO_2_ (R). This measurable electrical response to various external stimuli makes VO_2_ the prime candidate for electrical applications such as sensors or transistors. The following section discusses the electrical applications of VO_2_ and their corresponding devices.

##### 6.2.2.1 Sensors

**Figure 25:**
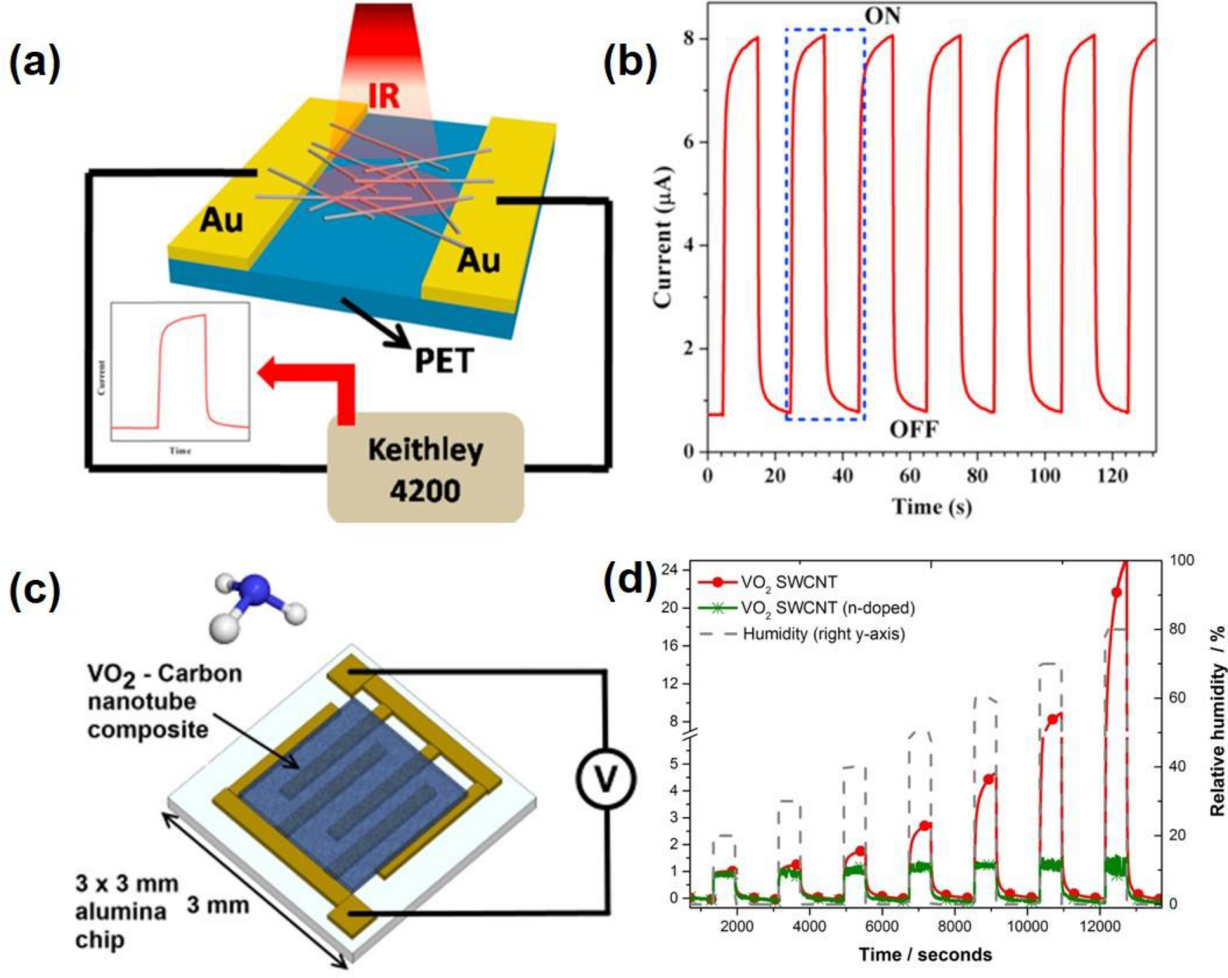
(a) Schematic of the VO_2_ nanowire photodetector. (b) Time-dependent photodetection capabilities of the VO_2_ nanowire device. Reproduced with permission from ref (202). Copyright 2018 Elsevier. (c) Schematic of the VO_2_/CNT gas sensor. (d) Time-dependent humidity sensing capabilities of the VO_2_/SWCNT and n-doped variant at different humidity levels. Reproduced with permission from ref (206). Copyright 2018 Elsevier.

A sensor is defined by its ability to measure physical input and translate these measurements into interpretable data. Based on the significant conductivity changing of VO_2_ across the MIT, it is possible to convert physical environmental input into a readable electrical signal. Some examples of VO_2_-based sensors include temperature sensors, photodetectors, flexible strain sensors, and gas sensors. Intrinsically, VO_2_ is not suitable for temperature sensing applications because the change in its electrical conductivity only happens at 68 °C, even though Kim et al.^200^ managed to fabricate a programmable VO_2_ critical temperature sensor. VO_2_ was deposited on an Al_2_O_3_ (1010) substrate and between two nickel (Ni) electrodes. A voltage can be applied across these electrodes to cause the τ_C_ of VO_2_ to decrease, causing the VO_2_ (M) film to go into an intermediate phase before fully transitioning into VO_2_ (R). During this intermediate phase, the measured current through the device was found to be linear with the change in temperature. At a voltage of 20 V, the τ_C_ is found to be ∼20 °C, enabling full range sensing capabilities from 20 to 68 °C. Another approach, which is based on the sensing ability resulting from an abrupt change of the dielectrically constant of VO_2_ during MIT instead of conductivity, was done by Yang et al.^201^ As mentioned in the previous section, optically stimulated applications of VO_2_ are promising due to the ultrafast transition mechanics, stability, as well as the broadband optical response of VO_2_-based devices. Hou et al.^202^ demonstrated the device stability and speed of response using a VO_2_ (M) nanowire on Au electrode setup (Figure 25a). It was reported that the device needs less than 1.6 s to detect IR (980 nm) and <1.0 s to recover (Figure 25b). The device was also reported to maintain responsivity for more than 500 cycles. Another design from Takeya et al.^203^ combined the photoresponsivity of VO_2_ film and the localized surface plasmon resonance of silver nanorods. The results indicated a correlation between the incident light transmission and resistivity within a wavelength of 400–900 nm. While the VO_2_ acted as a photosensitive component, the nanorod array introduced a wavelength and polarization sensitivity to the photodetector. Because the MIT of VO_2_ results in the change in the lattice structure and constant, it is also possible to induce MIT by causing changes to the lattice through mechanical force, which serves as the basis for VO_2_ flexible strain sensor application. Hu et al.^204^ showed that a VO_2_ strain sensor device could be fabricated by bonding one single VO_2_ nanobeam to a polystyrene (PS) substrate with silver paste and measuring the resistivity of the nanobeam as tensile and compressive stress is applied along the length of the nanobeam. In this study, VO_2_ (R) was not formed, and only the VO_2_ (M_2_) phase was formed due to the constraint of force applied (only 0.25% as compared to the required 2% at room temperature). However, the device showed remarkable potential when exhibiting a stepwise response to as small as 0.05% tensile or compressive strain.

A typical gas sensor design is demonstrated in Figure 25c. For gas sensor application, the semimetallic VO_2_ (B) phase is more commonly used than the insulating VO_2_ (M) phase to maintain sensing capability at room temperature. VO_2_, regardless of the phase, responds to humidity, ammonia (NH_3_), and nitrogen dioxide (NO_2_).^205,206^ Compositing VO_2_ with carbon species such as single- or multiwalled carbon nanotubes (SWCNTs or MWCNTs) was reported by Evans et al.^206^ The setup was effective in creating a stable, responsive VO_2_–CNT gas sensor. Figure 25d shows excellent response and good recovery of VO_2_–SWCNT to different humidity levels. The resistive response was increased dramatically from 0.5 for pure VO_2_ (B) to 2.7 for VO_2_–SWCNT and 7.1 for VO_2_-MWCNT at 50% humidity. This p-type gas sensing response was also reported for NH_3_ in the same study despite the longer and lower recovery level recorded. Depending on the applications, property change across the MIT is not the only viable option to use VO_2_ in a functional device.

##### 6.2.2.2 Electrical Switches, FETs, Oscillators, and Memristors

**Figure 26:**
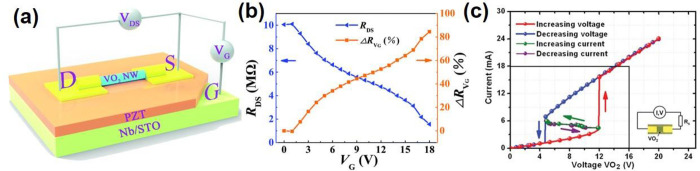
(a) Schematic of the VO_2_–NW-FeFET design. (b) Resistance change of the VO_2_–NW-FeFET ranging from 0 to 18 V. Reproduced with permission from ref (211). Copyright 2020 Royal Society of Chemistry. (c) *I*–*V* characteristic in voltage- and current-mode of a device incorporating a VO_2_ pattern; inset is the schematic test circuit. Reproduced with permission from ref (216). Copyright 2012 Cambridge University Press and the European Microwave Association.

Different from sensing applications, the MIT of VO_2_ can be deliberately triggered with programmable duration and patterns to great advantage in electrical switching, FET, oscillator, and memory devices. Similar to optical switches in the previous section, by toggling VO_2_ across the MIT, it is possible to create an ON/OFF switching mechanism based on the difference in electrical resistance of VO_2_ (M) and VO_2_ (R). It has been demonstrated by Zhou et al.^207^ that a two-terminal VO_2_-based switching device can have ultrafast, reliable 2 orders of magnitude ON/OFF toggling ability within 2 ns. While the MIT in this report was induced by an applied current, an electrical switch activated by Joule heating was also reported in a separate study by Li et al.^208^ Mott FET is a gated FET device in which the conventional semiconductor channel is swapped with a Mott insulator, a material with the ability to switch from insulator to metal through external voltage to the gate. VO_2_, as a Mott insulator, is the prime material for novel Mott FET studies. An example of a typical Mott FET setup was reported by Yajima et al.^209^ in which a large current modulation can be observed at 315 K, indicating a positive-bias gate-controlled MIT near τ_C_ of VO_2_. Another novel Mott FET design was also fabricated by Shukla et al.^210^ with a VO_2_ as a source terminal. This design functioned well at room temperature (300 K) with reversible MIT triggered by the critical applied current. Taking advantage of the VO_2_-based Mott FET designs and combining it with a ferroelectric material, Zhang et al.^211^ fabricated a nonvolatile ferroelectric FET (FeFET) device with VO_2_ nanowires as the channel and Pb(Zr_0.52_Ti_0.48_)O_3_ (PZT) thin film as the dielectric gate (Figure 26a). The novel FeFET device was reported to achieve up to 85% resistance change under a gate voltage of 18 V (Figure 26b). Interestingly, the presence of the ferroelectric materials created a polarization effect after the applied voltage was removed, in which the channel resistance could attain up to 50%. Through this mechanism, it is possible to achieve multiple resistive states by the sweeping suitable gate voltage. To overcome the disadvantage of solid-gate oxide dielectric FET, such as current leakage, which might interfere with the MIT of VO_2_, ionic liquid (IL) and solid-state electrolyte gating have been the research interest for VO_2_ FET devices in recent years.^212^ However, the mechanism in which IL drives the MIT of VO_2_ is still a debate between different studies. Nanako et al.^151^ suggested that the underlying mechanism is the bulk carrier delocalization caused by the electrostatic effect. On a different train of thought, Jeong et al.^213^ attributed the transition to the field-induced creation of oxygen vacancies, rather than the purely electrostatic effect. Ji et al.^214^ and Shibuya et al.,^215^ however, suggested that electrochemical protonation was the origin of the modulation of electrical property in VO_2_, similar to what was observed in the electrochromic setup in the previous section. An electronic oscillator is a common component in modern electronic circuitry which can produce periodic signals such as a square wave or a sine wave. Due to the periodicity of the output, it is often used to convert a direct current (DC) input into an alternate current output. The two main types of electronic oscillators are the linear (harmonic) and nonlinear (relaxation) oscillator. Because of the ability to undergo a nonlinear MIT, VO_2_ can be used as the basis for a nonlinear oscillator with a relaxation behavior stimulated by external electricity input. A VO_2_-based oscillator design by Leroy et al.^216^ and its *I*–*V* characteristic curve is shown in Figure 26c. The inset shows the schematic of the oscillator circuit in which a resistor (R_s_) is connected to the VO_2_ device to produce a current-controlled negative differential resistance (NDR). The NDR portion happens when VO_2_ enters the transitive state between VO_2_ (M) and VO_2_ (R). It was reported that by controlling R_s_, the VO_2_-based oscillator circuit can become self-sustaining, and the frequency can range from kHz to 1 MHz.^152^ Aside from the standard setup as shown in Figure 26c, studies have also been done in which two oscillators are coupled with a resistor, a capacitor, or FET in between.^217^ A memristor is a nonvolatile electronic memory device which has a programmable resistance. The resistance of a memristor is retained even after removal of the power and is dependent on the original applied voltage. It is crucial that the resistance can be reversed or reprogrammed. Thus, a two-terminal VO_2_ electrical device with the nonvolatile switching of resistance across the MIT can also be adapted into memristors.^149^ Bae et al.^150^ demonstrated a two-terminal memristor based on a single VO_2_ nanobeam. The nanobeam undergoes MIT when a bias of 3 and 5 V was applied for 0.25 s; the resistance of the device goes from an initial 10^11^ Ω to 10^9^ and 10^8^ Ω respectively. The resistance change can be reset with a zero-voltage bias. VO_2_ has also been utilized in other memory devices including a multistate free-standing VO_2_/TiO_2_ cantilever,^218^ resistive random-access memory (ReRAM) devices.^219^ and 3D memory array.^189^ Not included in the above discussion is the minor application of VO_2_ in field emitter and spintronic devices which are based on the abrupt drop in resistance across thermal- and magnetical-activated MIT, respectively. Studies on VO_2_/ZnO core–shell nanotetrapod thermal-activated field emitters were reported by Yin et al. in 2014.^220^ On the other hand, VO_2_-based spintronic devices and the behavior of the magnetoresistance of VO_2_ were reported in detail by Li et al.,^221^ Choi et al.,^222^ and Singh et al.^223^

#### 6.2.3 Mechanical Applications

**Figure 27:**
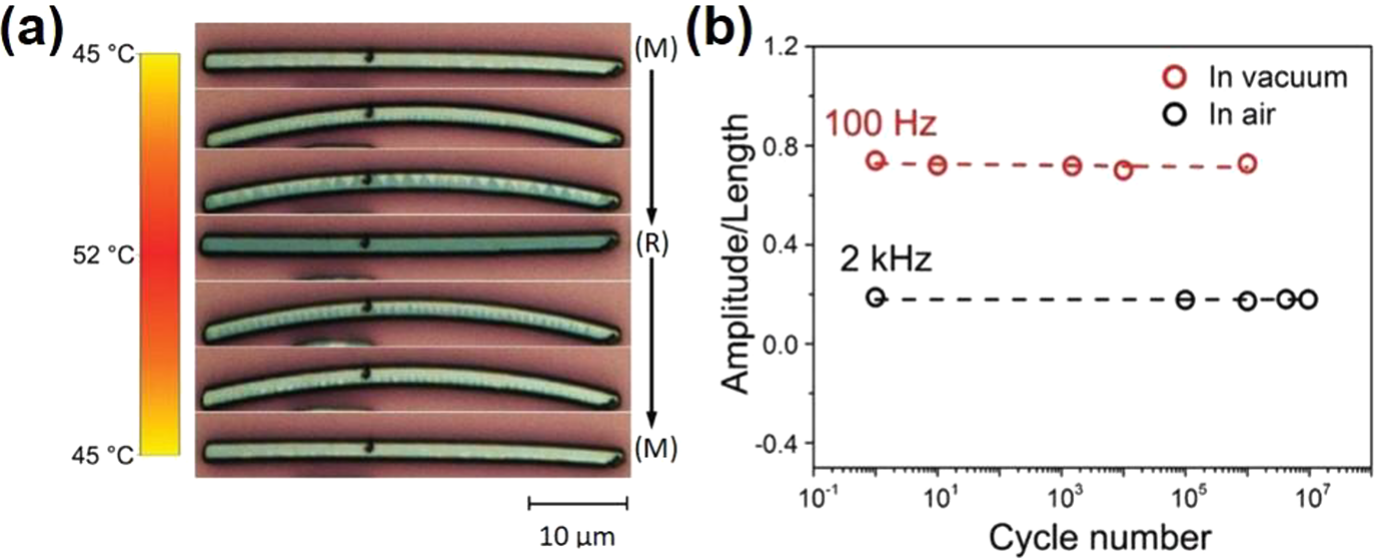
(a) Optical images of the VO_2_ nanobeam undergoing MIT. (b) Amplitude versus cycle number plots of the nanobeam subjected to a chopped laser (100 Hz in a vacuum and 2000 Hz in air). Reproduced with permission from ref (227). Copyright 2019 John Wiley and Sons.

The actuator is typically a component in a machine or a system which converts provided energy into mechanical motion. The concept of the actuator has been widely adapted into novel scientific research, especially in the field of microrobotics or micro-/nanoelectromechanics.^224^ VO_2_, which has a high theoretical work density (≈ 7 J cm^–3^) and fast response rate to external stimuli, is suitable for actuator applications. It offers the ability to offset disadvantageous low work density and the slow response rate of common actuator materials such as piezoelectric ceramics or polymers and CNT, respectively.^225^ In device fabrication, single crystal VO_2_ or composite bimorph of VO_2_ can be designed to respond to specific external stimuli such as light, heat, or electrical current. An example of a photodriven VO_2_ bimorph design was reported by Ma et al.^226^ in 2018. The VO_2_/CNC device was conceived by combining the carbon nanocoil (nanosprings twisted by hollow carbon nanofibers) core with a VO_2_ shell. When exposed to 980 nm radiation, the temperature of the spring increases unevenly, forming a temperature gradient from tip to end. This results in a transition gradient in which the tip becomes VO_2_(R) first and shrinks, creating the curvature. The VO_2_/CNC actuator delivers a large displacement-to-length ratio (∼0.4), fast response rate (9400 Hz), and long durability (>10^7^ cycles). More recently, Shi et al.^227^ fabricated thermal-activated single-crystalline VO_2_ actuators (SCVAs) which were designed so that the τ_C_ of a single VO_2_ nanobeam is a gradient along the radial direction. When exposed to heat, one side of the fabricated W-doped VO_2_ nanobeam with lower τ_C_ would undergo MIT first and shrink, creating a bending as seen in Figure 27a. It was reported that this SCVAs design performed competitively with other reported VO_2_ bimorph actuator designs with an extremely high displacement-to-length ratio (∼1), high energy efficiency (∼0.83%), fast response rate in the order of kHz, and long durability (>10^7^ cycles) (Figure 27b). VO_2_ electrothermal devices with joule heating activation for oscillator^228^ and microelectromechanical systems (MEMS)^229^ have also been fabricated with variable degrees of success. A resonator is a device that exhibits resonance at its eigenfrequency. With VO_2_, the eigenfrequency of a resonator can be dynamically controlled by thermally triggering the MIT. This specific frequency is positively related to Young’s modulus of VO_2_, which is widely different between the monoclinic phase (151 ± 2 GPa) and the rutile phase (218 ± 3 GPa).^230^ Studies have been made to determine the effects of elemental doping on the frequency modulating ability of VO_2_-based resonators. Rúa et al.^231^ compared the Cr-doped VO_2_ resonator with the undoped one and concluded that the doped sample had a higher frequency change due to a lower Young’s modulus. Other strategies to improve performance such as changing the shape from a simple cantilever have also been done by Manca et al.^232^

#### 6.2.4 Supercapacitors

**Figure 28:**
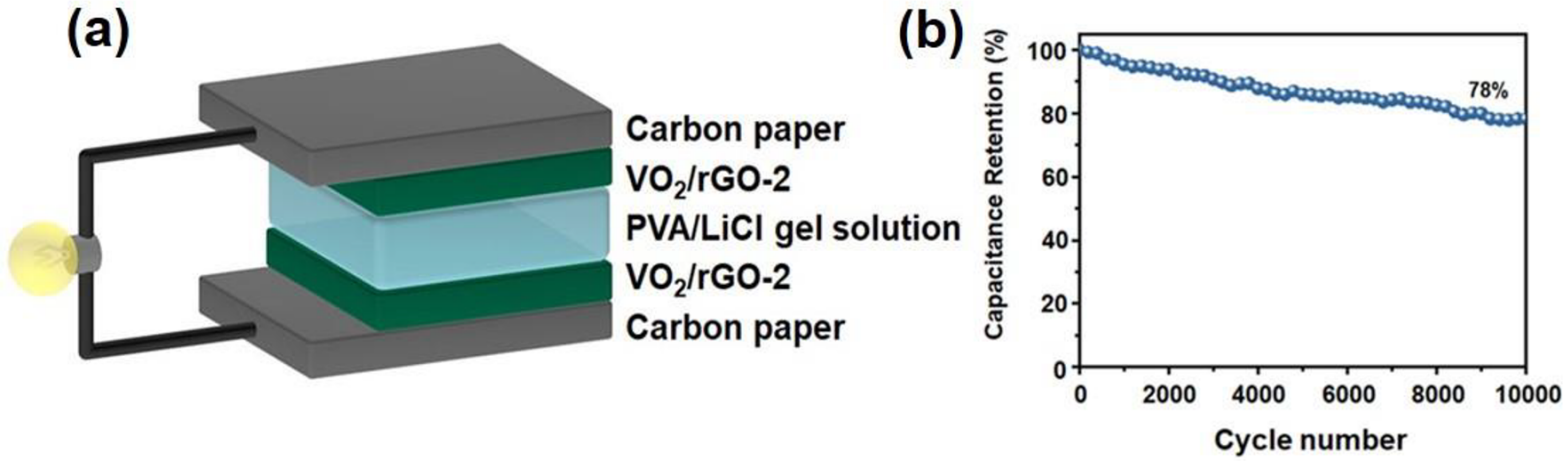
(a) Schematic illustration of the all-solid-state supercapacitor. (b) Cyclic stability test of the VO_2_/rGO supercapacitor device. Reproduced with permission from ref (235). Copyright 2019 under CC BY 4.0 license.

VO_2_ (B) with its layered structure and multioxidation states is ideal as an electrode material with charge storage through insertion and fast surface Faradaic reaction.^233^ However, being in common with all metastable VO_2_ phases, VO_2_ (B) structural instability is not suitable for cyclic stability of SC application. Multiple studies have been done to combine VO_2_ (B) with various carbon composites to create stable electrode materials.^234^ An example of a VO_2_ and reduced graphene oxide (VO_2_ (B)/rGO) device by Liu et al.^235^ is shown in Figure 28. The schematic diagram of the all-solid-state sandwich-structured supercapacitor design is shown in Figure 28a, where symmetrical VO_2_ (B)/rGO and PVA/LiCl gel are used as electrodes and electrolytes, respectively. The performance of this design was reported to have a superior specific capacitance of 353 F g^–1^ at 1 A g^–1^ and a maximum power density of 7152 W kg^–1^ at an energy density of 3.13 Wh kg^–1^. By compositing VO_2_ (B) with rGO, 78% capacitance was retained after 10000 cycles, improving the stability of the device immensely (Figure 28b).

#### 6.2.5 Magnetic Refrigeration

Magnetic refrigeration is a cooling technique that is based on the magnetocaloric effect (MCE). The MCE describes the phenomenon in which a suitable material can be heated up or cooled down when exposed to a changing magnetic field. Due to the changing magnetization when crossing the MIT, VO_2_ was first shown to be suitable for magnetic refrigeration application by Wu et al.^236^ in 2011 with a single crystalline nanorod fabrication technique. Although the potential was shown for VO_2_ in magnetic refrigeration applications, studies to further improve this are still limited.

#### 6.2.6 Batteries

**Figure 29:**
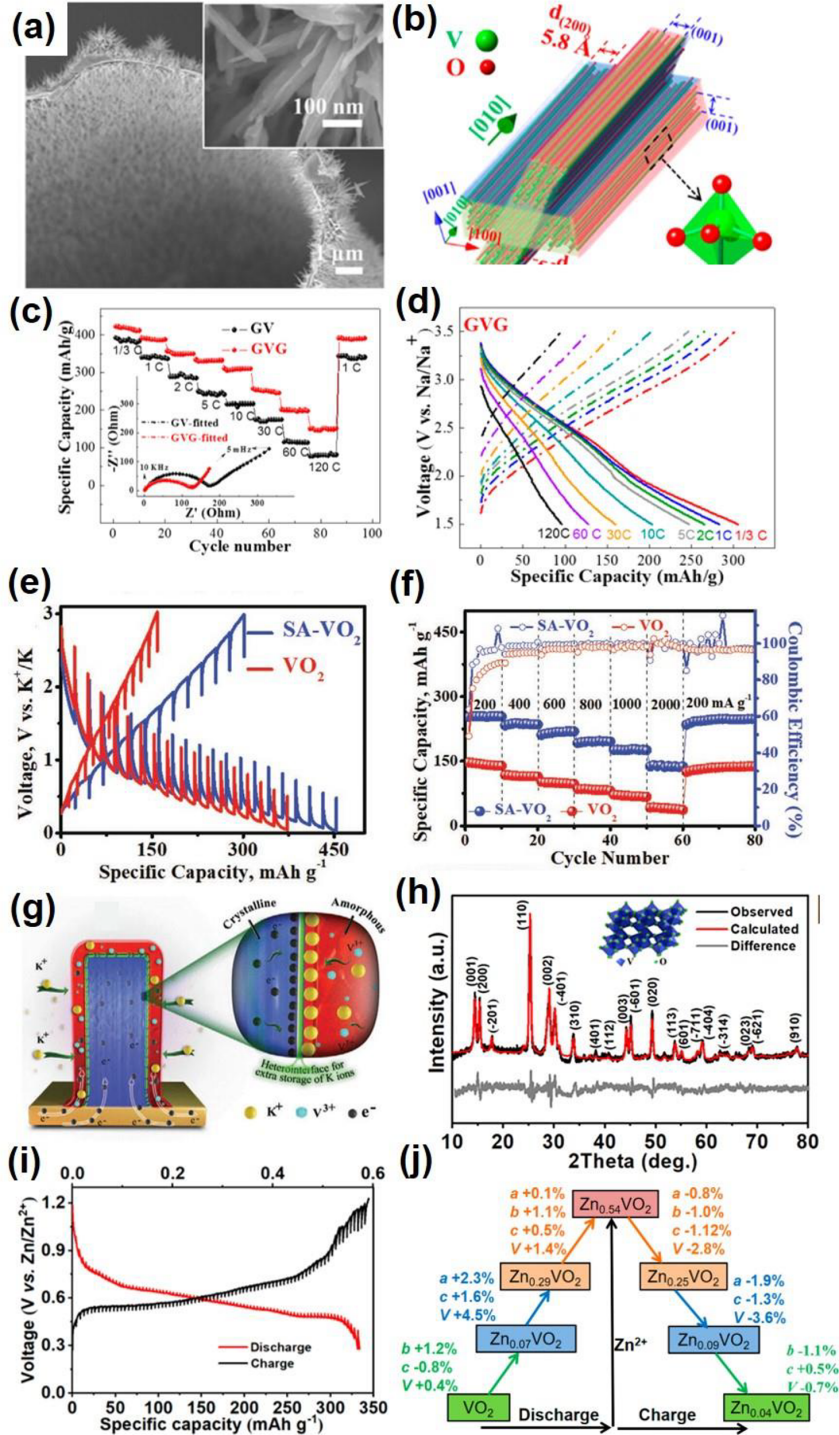
SEM image (a), geometrical model (b), LIB rate performance (c), and SIB charge/discharge profiles (d) of bilayered VO_2_ nanobelt. Reproduced with permission from ref (237). Copyright 2015 American Chemical Society. The GITT curves (e), rate performances (f) of SA-VO_2_ and VO_2_, and the schematic of the enhanced K ion storage ability (g) of SA-VO_2_. Reproduced with permission from ref (238). Copyright 2020 John Wiley & Sons. The Rietveld refinement result from the XRD data and crystallographic structure (h), GITT curve (i), and most significant changes of lattice parameter in each stage (j) of VO_2_. Reproduced with permission from ref (239). Copyright 2019 American Chemical Society.

VO_2_ formed by edge-sharing VO_6_ octahedra with a unique bilayer structure exhibits a large lattice spacing that can accommodate Li^+^ (0.76 Å), Na^+^ (1.02 Å), K^+^ (1.38 Å), and Zn^2+^ (0.74 Å) insertion/extraction. Fan et al.^237^ designed and synthesized a binder-free VO_2_ cathode via biface VO_2_ arrays directly growing on a graphene foam (GF) network (Figure 29a). They constructed a geometric model of bilayered VO_2_ nanobelts through the growth direction and lattice spacings (Figure 29b). The relatively high stacking rate of the “steplike” VO_6_ octahedra along the [010] direction determines the preferred growth direction. As a result, the (001) facet of the VO_2_ nanobelt is the thinnest, and the interlayers between the (200) crystal planes provide a facile channel for Li^+^ and Na^+^ diffusion. Meanwhile, the graphene quantum dots (GQDs) coating on the VO_2_ surfaces can act as highly efficient surface protection to further enhance the Li^+^/Na^+^ storage. When as-prepared GF-supported GQD-anchored VO_2_ arrays (GVGs) are directly used as a LIBs/NIBs cathode, it exhibits two advantages: high ion diffusion sensitization and charge transport kinetics are beneficial to obtain high-rate capacities, and the homogeneous GQDs suppress VO_2_ dissolution which is in favor of retaining long-term cycles. The GVG electrode delivers a high specific capacity of 421 mAh g^–1^ at 1/3 C for Li^+^, which is much higher than that of the uncoated GF@VO_2_ electrode (391 mAh g^–1^). It can maintain 151 mAh g^–1^ even at 120 C,and 94% of the initial capacity can be retained after 1500 cycles (Figure 29c). When it is used as an NIB cathode, it exhibits a specific capacity of 306 mAh g^–1^ at 1/3 C and good capacity retention (Figure 29d). These results demonstrate that the GVG is an excellent electrode material for Li^+^/Na^+^ storage.

Zhang et al.^238^ first designed and synthesized a surface amorphized VO_2_ (B) nanorod (SA-VO_2_) with a crystalline core and a surface-amorphized shell heterostructure by an interfacial engineering strategy. The crystalline/amorphous heterointerface in SA-VO_2_ substantially narrows the bandgap, lowers the surface energy, and reduces the K^+^ diffusion barrier of VO_2_ (B) via DFT calculations. Therefore, the as-obtained SA-VO_2_ electrode exhibits a higher reversible capacity of 288.3 mAh g^–1^ (at 50 mA g^–1^), superior rate capacity (141.4 mAh g^–1^), and long-term cyclability (86% after 500 cycles at 500 mA g^–1^) (Figure 29e), while the VO_2_ only delivers a specific capacity of 147.2 mAh g^–1^ at 50 mA g^–1^ and maintains 16.5% capacity after 200 cycles at 500 mA g^–1^ (Figure 29f). Compared with oxygen-rich defect amorphous shell VO_2_, the crystalline/amorphous heterointerface SA-VO_2_ enhances the K^+^ storage capacity and enables rapid K^+^/electron transfer, which results in large capacity and outstanding rate capability (Figure 29g).

Mai et al.^240^ reported VO_2_ hollow microspheres with a high surface area and excellent structural stability via a facile and controllable ion-modulating approach. VO_2_ hollow microspheres deliver the best Li^+^ storage performance compared to six-armed microspindles and random nanowires. The highest surface area of VO_2_ hollow microspheres can provide efficient self-expansion, self-shrinkage buffering, and self-aggregation during lithiation/delithiation, which delivers 3 times higher capacity than that of random nanowires. In addition, they also synthesized highly homogeneous VO_2_ nanorods by a rapid and simple hydrothermal method for aqueous ZIBs cathode material (Figure 29h).^239^ The *in situ* XRD and *ex-situ* XPS/TEM results demonstrate that the VO_2_ undergoes a single-phase reaction during the discharge process, accompanying a phase transition process of VO_2_–Zn_0.07_VO_2_–Zn_0.29_VO_2_–Zn_0.54_VO_2_ with a unit cell volume expansion of 6.69%. On the contrary, the evolution of Zn_0.54_VO_2_–Zn_0.25_VO_2_–Zn_0.09_VO_2_–Zn_0.04_VO_2_ occurs during the Zn^2+^ deintercalation from the Zn_0.54_VO_2_. Meanwhile, detailed qualitative analysis verified that the VO_2_ unit cell expands in the *a*, *b*, and *c* directions sequentially during the discharge/charge processes. Satisfactorily, the VO_2_ nanorods deliver a high specific capacity of 325.6 mAh g^–1^ and excellent long cycle performance (86% after 3000 cycles), which is outstanding performance among the reported cathode materials of the aqueous ZIBs (Figure 29i,j).

#### 6.2.7 HER, OER, and Water Splitting

VO_2_ is a well-known semiconductor material with a band gap of 0.7 eV, which is seldom considered as a candidate material as a catalyst or photocatalyst for the production of hydrogen.^241^ VO_2_ can be used as a photocatalyst for hydrogen evolution through phase engineering. Ajayan et al.^242^ synthesized the body-centered-cubic nanostructured VO_2_, which shows excellent photocatalytic activity with a hydrogen production rate up to 800 mmol m^–2^ h^–1^ from a mixture of water and ethanol under UV light at a power density of ∼27 mW cm^–2^. Furthermore, vanadium oxide composites have the great potential to accelerate water dissociation kinetics and reduce charge-transfer resistance.^243,244^ Tao et al.^245^ synthesized MoS_2_/VO_2_ hybrids by using a two-step hydrothermal method. The phase transition of VO_2_ exhibits a significant effect on hydrogen evolution properties of the heterostructures (an onset potential of 99 mV and a Tafel slope of 85 mV dec^–1^). The enhanced performance is mainly due to the faster electron transport as well as the strain effect on MoS_2_. Tu et al.^246^ fabricated Co_3_O_4_/VO_2_ heterogeneous nanosheet structures on carbon cloth (Co_3_O_4_/VO_2_/CC) by the combination of hydrothermal and electrodeposition methods. The Co_3_O_4_/VO_2_/CC composites gave good HER performance with a low overpotential of 108 mV at 10 mA cm^–2^ and a Tafel slope of 98 mV dec^–1^, which results from the abundant active sites, effective electron transport, and improved hydrogen binding energy. Najafi et al.^247^ prepared room temperature-stable metallic rutile VO_2_ nanosheets by the topochemical transformation of two-dimensional VSe_2_. By an O_2_ plasma pretreatment of the VSe_2_ nanosheets, the obtained VO_2_ nanosheets show a porous structure, which shows good HER and OER performances in either acidic or alkaline media. The symmetric two-electrode water splitting cell based on the porous VO_2_ nanosheets as both the anode and cathode delivers a current density of 10 mA cm^–2^ at cell voltages of 1.710 and 1.660 V in 0.5 M H_2_SO_4_ and 1 M KOH, respectively.

## 7 V_2_O_5_

### 7.1 Structures and Synthesis

**Figure 30:**
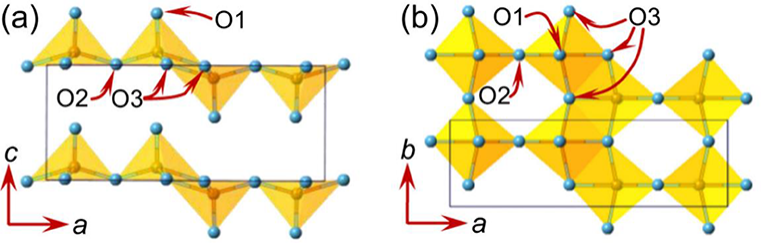
Perspective view (a) along the *b*-axis and (b) along the *c*-axis of two layers of V_2_O_5_, V atoms are gray balls, O atoms are red balls, and weak van der Waals bonds are omitted for clarity. Reproduced with permission from ref (249). Copyright 2018 Elsevier.

V_2_O_5_ has the highest oxygen state in vanadium–oxygen systems and is the most stable member of the series of vanadium oxides. V_2_O_5_ has multiple distinctive polymorphs, including α-V_2_O_5_ (orthorhombic), β-V_2_O_5_ (monoclinic or tetragonal), and γ-V_2_O_5_ (orthorhombic). Among them, the most common α-V_2_O_5_ is the thermodynamically stable phase (the unit cell structure belonging to the *Pmnm* space group with lattice parameters of *a* = 11.150 Å, *b* = 3.563 Å, and *c* = 4.370 Å), and the other two phases (β-V_2_O_5_ and γ-V_2_O_5_) can be transformed from the α-V_2_O_5_ phase under high pressure and high-temperature conditions.^248^ The orthorhombic structure of α-V_2_O_5_ is shown in Figure 30, in which each single layer of V_2_O_5_ consists of edge- and corner-sharing square pyramids, and the adjacent layers are bonded together along the *c*-axis by weak van der Waals bonds between the vanadium and oxygen of neighboring pyramids. Additionally, three different oxygens atoms, O_1_, O_2_, O_3_, have different coordinations depending on the position in each layer. The terminal/apical and bridging (corner-sharing) coordinated vanadyl oxygen atoms O_1_ and O_2_ have V–O bond lengths about of 1.54 and 1.77 Å, respectively; the triply coordinated O_3_ links three vanadium atoms via edge-sharing VO_5_ square pyramids, and the three corresponding V–O bond lengths are 1.88, 1.88, and 2.02 Å.^249^

The outstanding characteristics of V_2_O_5,_ such as a layered structure, a direct band gap in the visible-light region, high chemical and thermal stability, electrochemical safety, low cost, and easy preparation, make V_2_O_5_ a suitable material for electrochemical energy conversion and storage,^250^ catalysis,^251−253^ solar cells,^254^ gas sensors,^31^ electrochromic devices,^255^ and optoelectronic devices.^256^ Compared with bulk V_2_O_5_, the nanostructured ones have higher surface to volume ratios, which is beneficial to improve various performances. Over the past few years, a variety of methods, such as sol–gel, hydrothermal, chemical vapor deposition, magnetron sputtering, and atomic layer deposition, have been developed to prepare V_2_O_5_ nanostructures.

The sol–gel method has been used to fabricate V_2_O_5_ thin films and nanopowders through V_2_O_5_ sols, which were prepared by ion exchange, alkoxide hydrolysis, peroxide-assisted hydrolysis, and melt-quenching. The disadvantage of using an ion exchange is the difficulty to control the vanadium concentration as it varies throughout the whole process. In addition, some foreign ions such as Na^+^ may remain in the gel after ion exchange.^257^ The alkoxide hydrolysis always involves some expensive raw materials, and the molten V_2_O_5_ quenching process will produce toxic gas.

**Figure 31:**
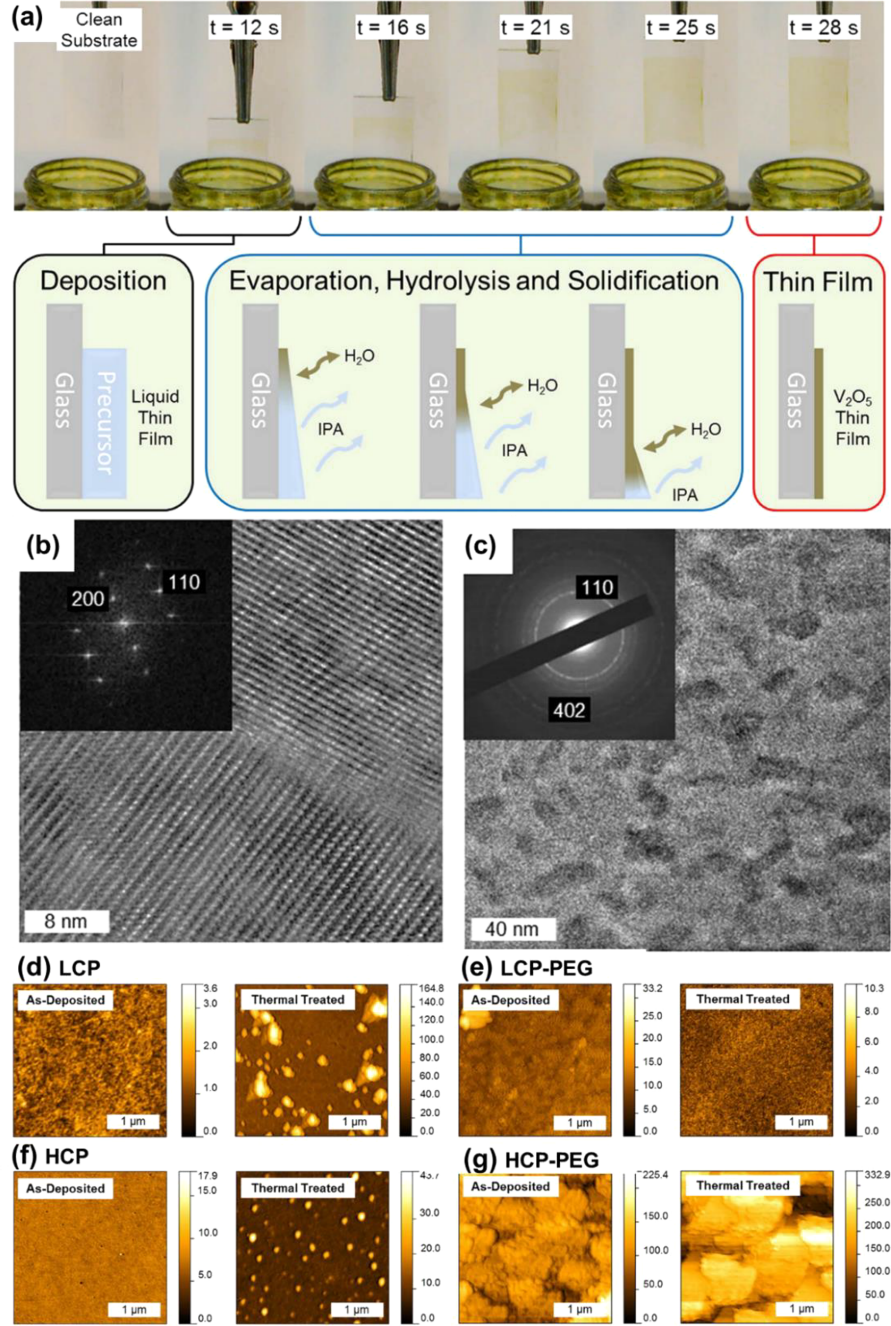
(a) The formation process of V_2_O_5_ thin film through a combination of evaporation, hydrolysis, and solidification is shown optically and schematically; TEM diffraction patterns and corresponding FFT of V_2_O_5_ thin films formed from (b) low concentration dilution (LCP) and (c) high concentration dilution using PEG-400 as an additive (HCP-PEG); AFM surface images of as-deposited and post-thermally treated orthorhombic V_2_O_5_ films that were dip-coated from (d) LCP, (e) LCP-PEG, (f) HCP, and (g) HCP-PEG solutions. Reproduced with permission from ref (265). Copyright 2015 under CC BY 4.0 license.

Hence the synthesis of V_2_O_5_ sol by peroxide-assisted hydrolysis stands out due to its advantages of being environmental friendly, inexpensive, and requiring simple fabrication. Vanadium metal or commercial V_2_O_5_ powders are commonly used as a vanadium source that can be dissolved vigorously in a solution of hydrogen peroxide. According to Alonso et al. the dissolution of V_2_O_5_ into H_2_O_2_ produces unstable diperoxo [VO(O_2_)_2_]^−^ and then is dissociated to the aqueous solution of [VO_2_]^+^ and [H_2_V_10_O_28_]^4–^.^258^ Etman et al. also found [H_2_V_10_O_28_]^4–^ is the main species via real-time nuclear magnetic resonance.^259^ It is noted that, not related to the preparation method, the V_2_O_5_ sols are comprised of a fibrous structure, dissimilar from many inorganic sols that are typified by a random aggregate of particle structure.^260^ These fibrous structures can self-assemble into V_2_O_5_ nanofibers upon long-term aging through a coagulation mechanism.^261^ The obtained V_2_O_5_ sol was applied onto substrates via coating techniques, including spin coating, dip-coating, and spray process, and the subsequent drying and heat treatment are necessary to obtain V_2_O_5_ films.^262−264^Figure 31a highlight the evaporation, hydrolysis, and subsequent solidification procedure during the formation of V_2_O_5_ thin films by dip-coating.^265^Figure 31b,c shows TEM diffraction patterns and corresponding FFT of V_2_O_5_ thin films formed from low concentration dilution (LCP) and high concentration dilution (HCP) using PEG-400 as an additive (HCP-PEG), respectively. V_2_O_5_ thin films formed from an LCP precursor show the formation of grains of orthorhombic V_2_O_5_ with defined grain boundaries (Figure 31b), while the HCP-PEG samples have a polycrystalline structure without uniformed grain (Figure 31c). Meanwhile, as shown in AFM images in Figure 31d–g, thin films formed from higher concentration precursors have a larger surface roughness (*R*_S_). Thermal treatment can further increase the *R*_S_ due to the formation of crystallites on the thin film surface. The thin film formed from LCP-PEG precursor has the lowest *R*_S_ of 0.2 nm. Liu et al. studied the substrate effect on the structure and electrical properties of nanocrystalline V_2_O_5_ thin films prepared by the sol–gel method.^266^ They found the annealed V_2_O_5_ film on the Si substrate exhibited more uniform rod-like morphology, and electrical measurements indicated the typical n-type semiconducting behavior. Senapati et al. prepared nanoscale V_2_O_5_ films having thicknesses ranging from 92 to 137 nm by spin coating V_2_O_5_ sol at different stages of aging.^267^ They reported the decrease of strain in the films with aging, and the electrical conductivity increased with aging due to the improved crystallinity of the films.

**Figure 32:**
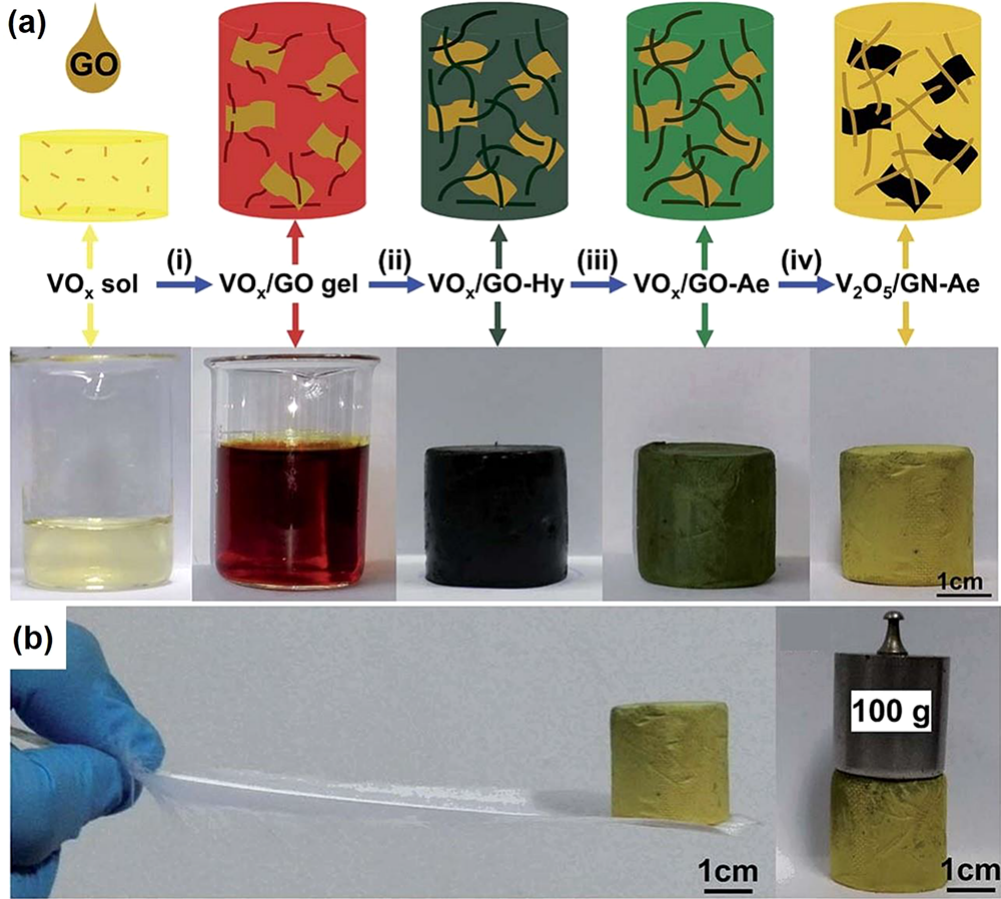
(a) The fabrication process and structure of the V_2_O_5_/graphene hybrid aerogel and the corresponding digital images of different formation stages: (i) hydrolysis of VO_*x*_ oligomers and self-assembled coordination of VO_*x*_ nanofibers and graphene oxide sheets; (ii) aging of VO_*x*_/graphene oxide gel and growth of VO_*x*_ nanofibers along graphene oxide sheets; (iii) solvent replacement and drying, and (iv) thermal reduction of graphene oxide, oxidation, and partly crystallization of V_2_O_5_. (b) Lightweight V_2_O_5_/graphene hybrid aerogel standing on a feather; it can support the weight of 100 g. Reproduced with permission from ref (273). Copyright 2015 Royal Society of Chemistry.

Besides thin films, the sol–gel method also provides good control over the size, morphology, doping, and chemical composition of V_2_O_5_ powders.^268−271^ Li et al. reported the flower-like V_2_O_5_ powders prepared by coagulating V_2_O_5_ sol and subsequent annealing crystallization.^272^ V_2_O_5_/graphene hybrid aerogel was prepared by Wu et al. at ambient pressure through a simple one-pot sol–gel method from commercial V_2_O_5_ powder.^273^Figure 32a illustrates the fabrication process and images of VO_*x*_ nanofibers and graphene oxide sheets. First, graphene oxide (GO) aqueous solution was added to the V_2_O_5_ sol under vigorous stirring to induce hydrolysis and in situ recombination of the GO sheets and VO_*x*_ oligomers. Then, a dark red VO_*x*_/GO hybrid gel was obtained after about 5 min because of the rapid formation of intermediate vanadium phases and the growth of nanofibers. After aging for 2 days, the gel gradually changed to deep green, and the V_2_O_5_ nanofibers are anchored and in situ grown on the graphene surfaces. The V_2_O_5_/graphene hybrid aerogel is so light that it can be lifted by a feather, but it is strong enough to support the weight of 100 g (Figure 32b).

**Figure 33:**
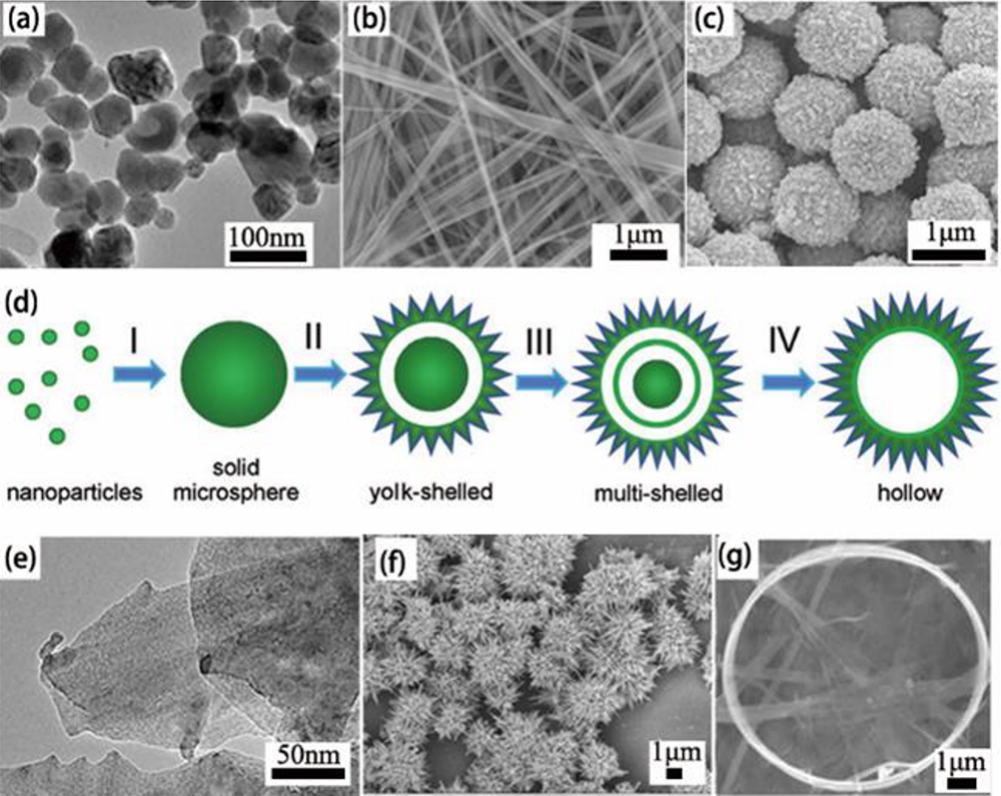
V_2_O_5_ with different morphologies: (a) Nanoparticles. Reproduced with permission from ref (274). Copyright 2015 Elsevier. (b) Ultralong nanobelts. Reproduced with permission from ref (261). Copyright 2011 Royal Society of Chemistry. (c) Hollows spheres and (d) growth mechanism. Reproduced with permission from ref (281). Copyright 2013 Wiley. (e) 2D nanosheets. Reproduced with permission from ref (283). Copyright 2015 Elsevier. (f) Urchin-like microflowers. Reproduced with permission from ref (284). Copyright 2012 American Chemical Society. (g) Nanoring. Reproduced with permission from ref (285). Copyright 2010 under CC BY 2.0 license.

The hydrothermal method has been widely used for the synthesis of a vast range of V_2_O_5_ nanostructures with a desired size and morphology, such as nanoparticles,^274^ nanowires,^275^ nanotube,^276^ nanosheets,^277^ and micro-/nanostructures.^278,279^ The importance of solubility of precursors, the pH value, the surfactant, as well as the hydrothermal temperature, reaction time, and solution filling factor are highlighted in many references. Li’s research group conducted extensive research on the hydrothermal treatment of V_2_O_5_ sol.^280^ They prepared V_2_O_5_ nanoparticles and ultralong nanobelts with the usage of an inorganic V_2_O_5_ sol precursor (Figure 33a,b).^261,274^ The obtained single-crystalline V_2_O_5_ nanobelts have a large specific surface area, with width and thickness of 30–200 nm and length in millimeters or even longer (Figure 33b). Strong evidence suggested that the oriented attachment growth mechanism was responsible for the formation of V_2_O_5_ nanobelts. Pan et al. reported a one-step solvothermal method to form V_2_O_5_ hollow spheres without adding surfactants.^281,282^ As shown in Figure 33c, the SEM image of the V_2_O_5_ hollow spheres has a uniform size of around 1 μm in diameter. They investigated the time-dependent interior structural evolution by TEM and gave the possible growth mechanism of the V_2_O_5_ microspheres (Figure 33d): vanadium oxide nanoparticles are first generated by the hydrolysis of VOC_2_O_4_ and then aggregation to form solid microspheres in stage I. The solid spheres undergo the initial inside-out Ostwald ripening process and transform to the yolk–shelled structure (stage II). With extended solvothermal reaction, secondary Ostwald-ripening takes place on the preformed solid cores, resulting in the formation of a multishelled structure (stage III). Finally, completely hollow microspheres are obtained as a result of the thorough dissolution and recrystallization of the less stable interior architectures (stage IV). Hence, the interior structure of the VO_2_ microspheres could be effectively tailored by simply controlling the reaction duration and concentration of the precursor.

The fabrication of two-dimensional (2D) V_2_O_5_ nanosheets has been studied by Cao et al.^283^ As cathode materials for lithium-ion batteries, the resulting 2D V_2_O_5_ nanosheets (Figure 33e) exhibit remarkable electrochemical performances, including high reversible capacity, good cyclic stability, and great rate capability. 3D hierarchical vanadium oxide microstructures, including urchin-like microflowers (Figure 33f), have been successfully synthesized by Lou et al. through a solvothermal method.^284^ The morphologies of the microstructures can be easily tailored by varying the concentration of the vanadium oxalate solution, and the obtained V_2_O_5_ microflowers are highly porous with a surface area of 33.64 m^2^ g^–1^ giving high lithium storage capacity, and enhanced cycling stability and rate capability. V_2_O_5_ nanorings and microloops are rarely reported, but they are very interesting morphologies (Figure 33g). The cation-induced asymmetric strain is the main driving force in making a layered V_2_O_5_ coil into a ring structure.^285^

**Figure 34:**
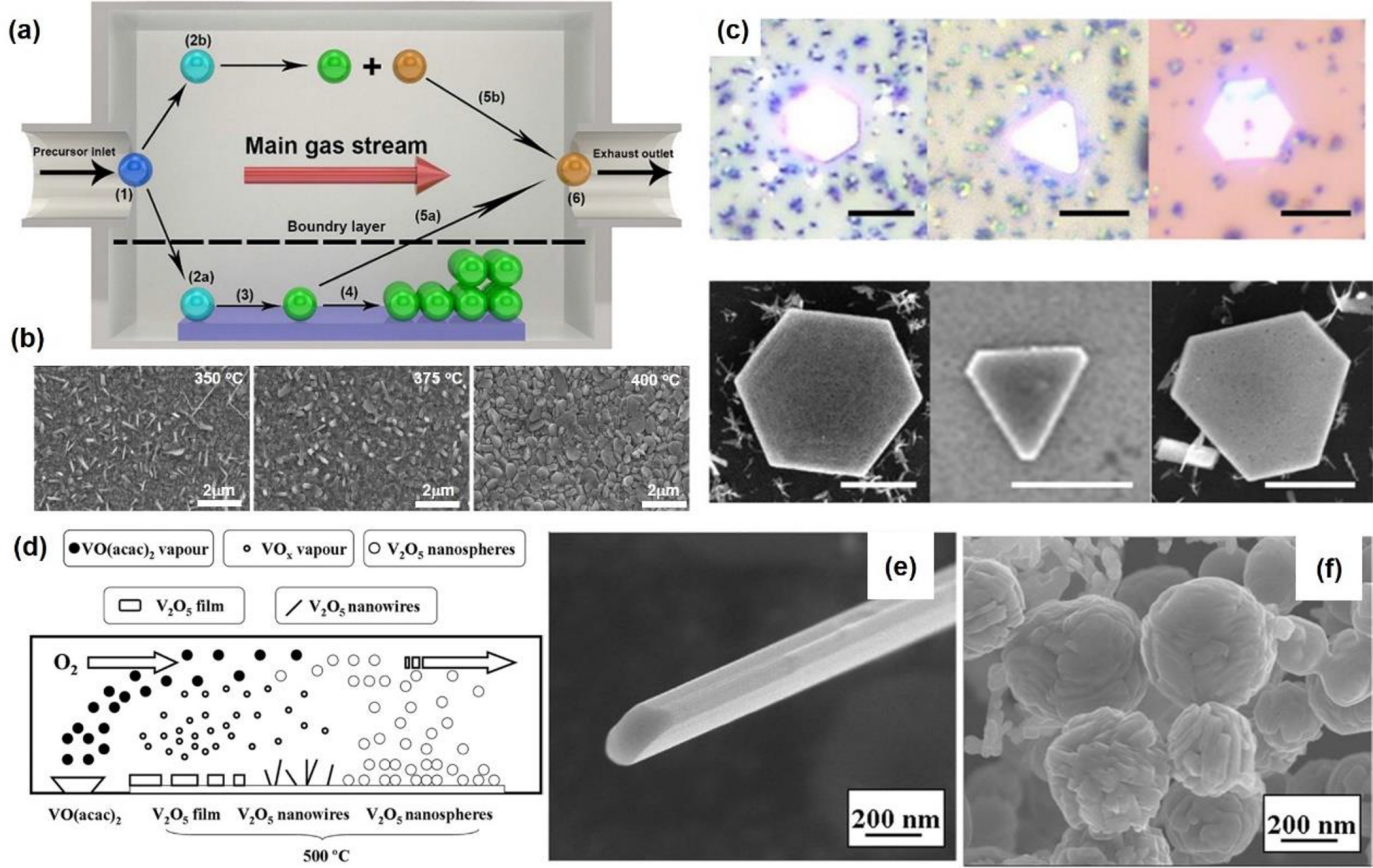
(a) Schematic diagram of the CVD process. Reproduced with permission from ref (19). Copyright 2018 Elsevier. (b) SEM images of AACVD grown V_2_O_5_ films at 350 °C, 375 °C, 400 °C. Reproduced with permission from ref (287). Copyright 2016 Elsevier. (c) Representative optical and SEM images of the as-synthesized V_2_O_5_ nanosheets with well-defined shapes, such as hexagon, triangle, and truncated triangle. All scale bars are 3 μm. Reproduced with permission from ref (288). Copyright 2018 American Chemical Society. (d) The growth schematic diagram of the V_2_O_5_ nanomaterials prepared by chemical vapor deposition using VO(acac)_2_ powder as the precursor, and the formed (e) nanowire and (f) nanospheres at a distance of 18 and 30 cm away from the source, respectively. Reproduced with permission from ref (289). Copyright 2010 Institute of Physics.

CVD is widely used for depositing high-quality and high-performance solid materials. As shown in Figure 34a, CVD involves the transfer of precursor molecules, which are either liquid or gaseous to a reaction chamber by a carrier gas (step 1), and then it is followed by the reaction and/or decomposing on the surface of the substrate to produce the desired films (steps 2a, 3, and 4) or powders (step 2b), and the byproducts and unreacted precursor are transported out from the chamber at the end of the process (step 5a, 5b).^19^ There are several types of CVD systems such as atmospheric pressure CVD, aerosol-assisted chemical vapor deposition (AACVD), atomic layer CVD, plasma enhanced CVD, metal–organic CVD, and so forth.^286^ The morphology, size, crystal phase, and specific surface area of V_2_O_5_ can be affected by various parameters, namely the reaction time, substrate temperature, pressure, precursor properties, and reaction position during the CVD method. SEM images in Figure 34b display the effect of growth temperature on the morphological characteristics of V_2_O_5_ coatings. V_2_O_5_ grown at 350 and 375 °C has rod-like structures of nonuniform thickness and width, while at 400 °C pellet-like features of V_2_O_5_ are observed, and the morphology evolution could be due to the coexistence of both α-V_2_O_5_ and β-V_2_O_5_.^287^ Chun et al. prepared V_2_O_5_ nanosheets via the reaction of VCl_3_ vapor with oxygen in the CVD system without a vacuum system.^288^Figure 34c shows representative optical images and their corresponding SEM images of the obtained V_2_O_5_ nanosheets and three distinguished shapes: hexagons, triangles, and truncated triangles. Wang et al. used the CVD method to control the morphologies of V_2_O_5_ by changing the reaction distance from the source position using vanadyl acetylacetonate (VO(acac)_2_) as the vanadium precursor.^289^ They found that VO_*x*_ vapor and VO(acac)_2_ vapor existed simultaneously during the growth process, and the different supersaturation distributions of these two vapors led to three main growth areas. 1. V_2_O_5_ thin-films were formed at a high concentration and supersaturation of VO_*x*_ in the region near the source material (Figure 34d); 2. nanowires with a length of about 10 μm and width about 200 nm were formed at a distance of 18 cm from the source due to the low vapor concentration of VO_*x*_ (Figure 34e); 3. nanospheres with a diameter of about 200–500 nm were obtained when the source material was far away (about 30 cm) due to the high concentration and supersaturation of VO(acac)_2_ that was oxidized to V_2_O_5_ nanospheres (Figure 34f).^289^

**Figure 35:**
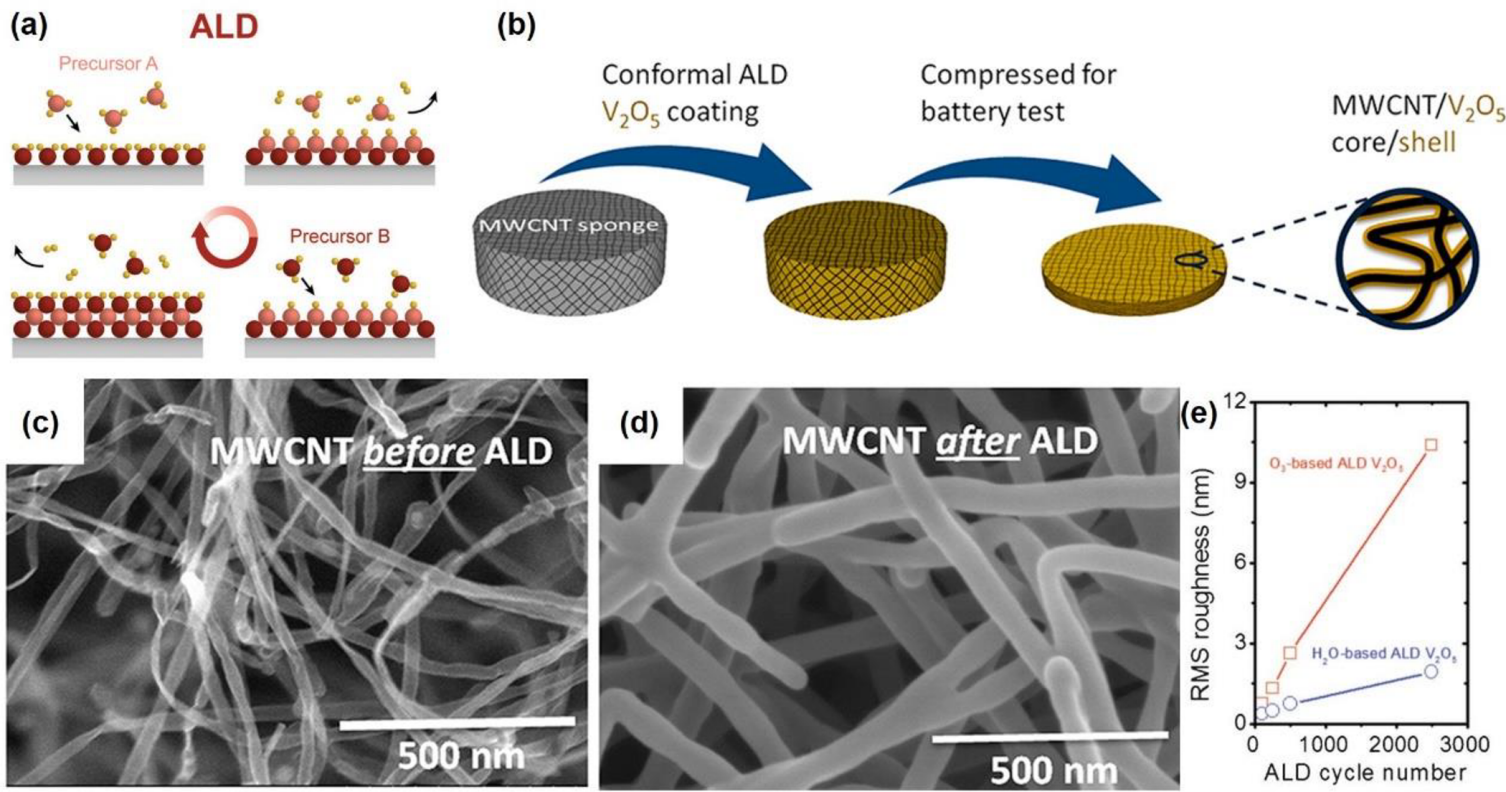
(a) Schematic of a single ALD cycle consisting of half-cycles of Precursor A and Precursor B separated by purge steps to remove excess precursor and byproducts. Reproduced with permission from ref (290). Copyright 2020 Royal Society of Chemistry. (b) Schematic of experimental flow to fabricate MWCNT/V_2_O_5_ sponge, and SEM images of MWCNT sponge (c) before and (d) after ALD V_2_O_5_ coating. Reproduced with permission from ref (291). Copyright 2012 American Chemical Society; (e) Compares the RMS roughness of the O_3_-based and the H_2_O-based films prepared by ALD as a function of cycle number. Reproduced with permission from ref (293). Copyright 2013 Royal Society of Chemistry.

ALD is considered a specific type of CVD, which was first introduced in the 1960s and is currently receiving ever-growing attention as a method of choice for the growth of conformal coatings on nanostructures with high aspect ratios.^12^Figure 35a depicts the typical ALD process, in which the precursor gases sequentially react with a surface to form an ultrathin film through a self-limiting process, and all byproducts and unreacted precursor molecules are purged out of the reactor.^290^ The primary advantages of ALD lie in subnanometer film thickness and conformality control that profit from the cyclic, self-saturating nature of ALD processes. Moreover, the ALD is unique as it is able to coat complex 3D structures with a high degree of uniformity and smoothness.^291,292^ Chen et al. successfully fabricated a multiwall carbon nanotube (MWCNT)/V_2_O_5_ core/shell sponge by ALD.^291^Figure 35b shows the experimental flow schematically: the MWCNT sponge structure exhibits a very low density (∼7 mg/cm^3^) and high porosity (>99%), allowing for a high amount of active material loading; the V_2_O_5_ layer of about 16 nm is subsequently deposited on the MWCNT sponge by 1000 cycles of H_2_O-based ALD; finally, the MWCNT/V_2_O_5_ sponge is compressed and assembled in a coin cell battery, which enables de/lithiation in active material within a very short time. Figure 35c,d shows SEM images of the MWCNT sponge before and after ALD V_2_O_5_ coating, giving uniform and smooth V_2_O_5_ coating. Two different oxidants, O_3_ and H_2_O, have been studied during the ALD process, and it was found that as the ALD cycle numbers increased from 100 to 2500, the roughness for the O_3_-based films kept increasing from 0.7 to 10.4 nm while that for H_2_O-based films only increased from 0.4 to 1.9 nm (Figure 35e).^293^ Østreng et al. prepared V_2_O_5_ films by ALD using the β-diketonate VO(thd)_2_ and ozone as precursors.^294^ They found that the crystallographic orientation, optical properties, band gap, and surface roughness of the V_2_O_5_ films were correlated and could be varied by controlling the deposition temperature and film thickness.

**Figure 36:**
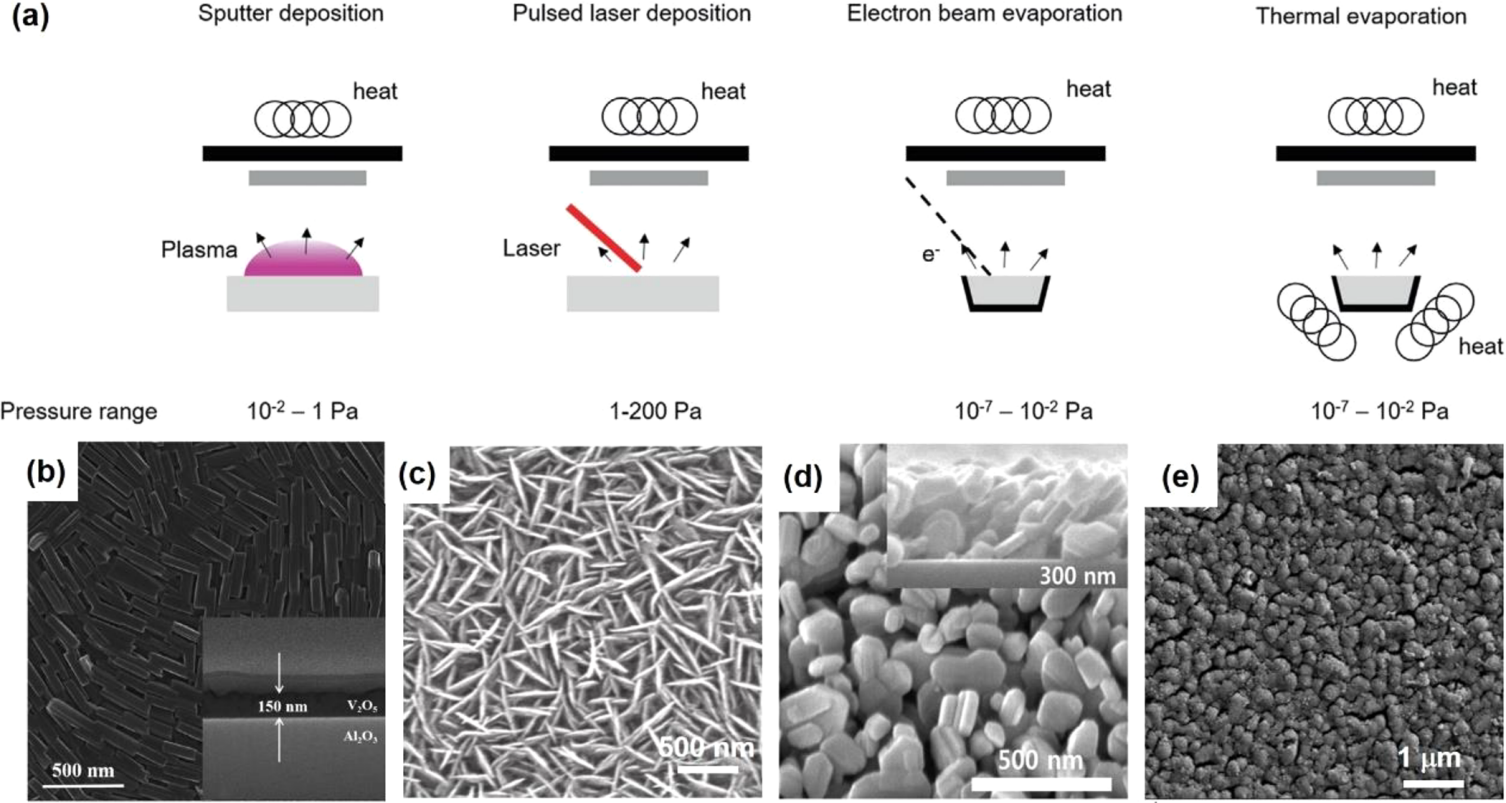
(a) Schematic illustration of different physical vapor deposition techniques: sputter deposition, pulsed laser deposition, electron beam evaporation, and thermal evaporation. In evaporation, atoms are removed from the source by thermal means, while in sputtering they are dislodged from a solid target via bombardment by gaseous ions. Reproduced with permission from ref (296). Copyright 2021 John Wiley and Sons. SEM images of V_2_O_5_ prepared by (b) megnetron sputtering. Reproduced with permission from ref (297). Copyright 2021 Elsevier. (c) PLD. Reproduced with permission from ref (299). Copyright 2021 Elsevier. (d) Electron beam evaporation. Reproduced with permission from ref (301). Copyright 2017 Elsevier. (e) Thermal evaporation. Reproduced with permission from ref (306). Copyright 2019 American Chemical Society.

PVD techniques (Figure 36a) involve evaporation and many different modes of physical sputter deposition, in which the primary source of the depositing species is a solid or liquid, as opposed to generally gaseous precursors in CVD. However, chemical reactions can and do occur in PVD systems, such as in the reactive sputtering deposition. The presence of the reactive gas (oxygen or nitrogen) in the chamber can significantly alter the PVD source.^295^ PVD possesses some unique advantages for the creation of uniform and dense solid thin films that strongly adhere to the substrates. Meanwhile, the thickness, composition, crystallinity, and crystal orientation of the thin film can be well controlled by changing the growth conditions with minimal risk of contamination due to the absence of organic reactants. Another advantage is to sequentially deposit several materials to form well-defined multilayer systems as well as special alloy compositions and structures.^296^ Magnetron sputtering is one of the most used PVD methods to fabricate a large range of materials, including metal oxides. The application of a negative voltage to the cathode will generate positively charged argon ions that can bombard the target ions to be ejected toward the substrate to form a film. Magnets are used in order to increase ion bombardment. This technique has been developed on an industrial scale to make large surface deposits with a wide variety of materials. V_2_O_5_ film consisted of fine long strip particles deposited by radio frequency magnetron sputtering, and the thickness of the V_2_O_5_ film was determined to be approximately 150 nm according to the cross-sectional SEM image (Figure 36b). V_2_O_5_ films underwent four different thermal transition behaviors to other vanadium oxides that were closely related to the oxygen proportion of the annealing ambient.^297^ Amorphous V_2_O_5_ film can be used as a hole injection layer in quantum dot light-emitting diodes, which exhibited a maximum luminance of 198.5 cd/m^2^, a turn-on voltage of 1.7 V, and a max external quantum efficiency of about 8.3%.^298^

Pulsed laser deposition (PLD) is another PVD method, which has been preferred to grow different structures ranging from high-quality epitaxial thin films to various nanostructured layers. During PLD, a high-power pulsed laser beam is focused on a target of the desired composition. Material vaporized from the target is deposited as a thin film on a substrate that faces the target in an ultrahigh vacuum (UHV) environment. Polycrystalline V_2_O_5_ thin film in the desired orientation can be prepared by PLD, which has aligned nanorod morphology on a flexible stainless steel substrate (Figure 36c).^299^ Huotari et al. found the film surface morphology varied largely according to oxygen partial pressure: lower O_2_ partial pressures resulted in a denser and thinner film, while higher O_2_ partial pressures gave a film surfaces formed with randomly agglomerated nanoparticles or agglomerates with pillar-like morphology.^300^

Electron-beam deposition (EBD) is another form of PVD where a target anode is bombarded with a high-energy electron beam that is given off from a charged tungsten filament under a high vacuum. The electron beam leads to joule heating and converts the target into the gaseous phase, which subsequently precipitates into the solid form on the desired substrate. Han et al.^301^ reported the growth of nanocolumnar V_2_O_5_ molecules that were aggregated with each other and collapsed after annealing treatment (Figure 36d). Most of the nanosized V_2_O_5_ columns’ structure could retain its original shape during the annealing process by changing the source from V_2_O_5_ to VO_2_. Highly oriented V_2_O_5_ thin films with nanosized grains were grown by EBD, and the film thickness was found to be in the range of 800–1200 nm that varied by adjusting the substrate position.^302^ Meanwhile, the mobility and carrier concentration of the oriented V_2_O_5_ thin films increased with the increase of V_2_O_5_ film thickness. Thermal evaporation is the vaporization of a material by heating to a temperature that the vapor pressure becomes appreciable, and the materials are sublimated from the target surface in a vacuum. By this method, heterojunctions,^303^ nanorods,^304^ nanoparticles,^305^ or highly crystalline V_2_O_5_ films^306^ have been studied in several reports. Wang et al. synthesized Ga-doped V_2_O_5_ nanorods by thermal evaporation at 850 °C and found interstitial Ga and Ga–O phases influence the photoluminescence properties of V_2_O_5_ nanorods.^304^ Berouaken et al. used thermal evaporation to prepare V_2_O_5_ nanoplatelets on the quartz crystal microbalance, followed by rapid thermal annealing.^307^ The obtained V_2_O_5_ nanoplatelets exhibited good sensing performance toward NH_3_ vapor at room temperature: a fast response time, a short recovery time, good stability, reproducibility, reversibility, and linearity. Velmurugan et al. prepared highly crystalline V_2_O_5_ films with a controlled thickness of about 530 nm and an average particle size of around 560 nm using a thermal evaporation process (Figure 36e).^306^ The films were fabricated in electrochemical microcapacitors and subjected to various electrochemical characterizations, which display improved reliability and excellent capacitance retention.

### 7.2 Applications

#### 7.2.1 Batteries

**Figure 37:**
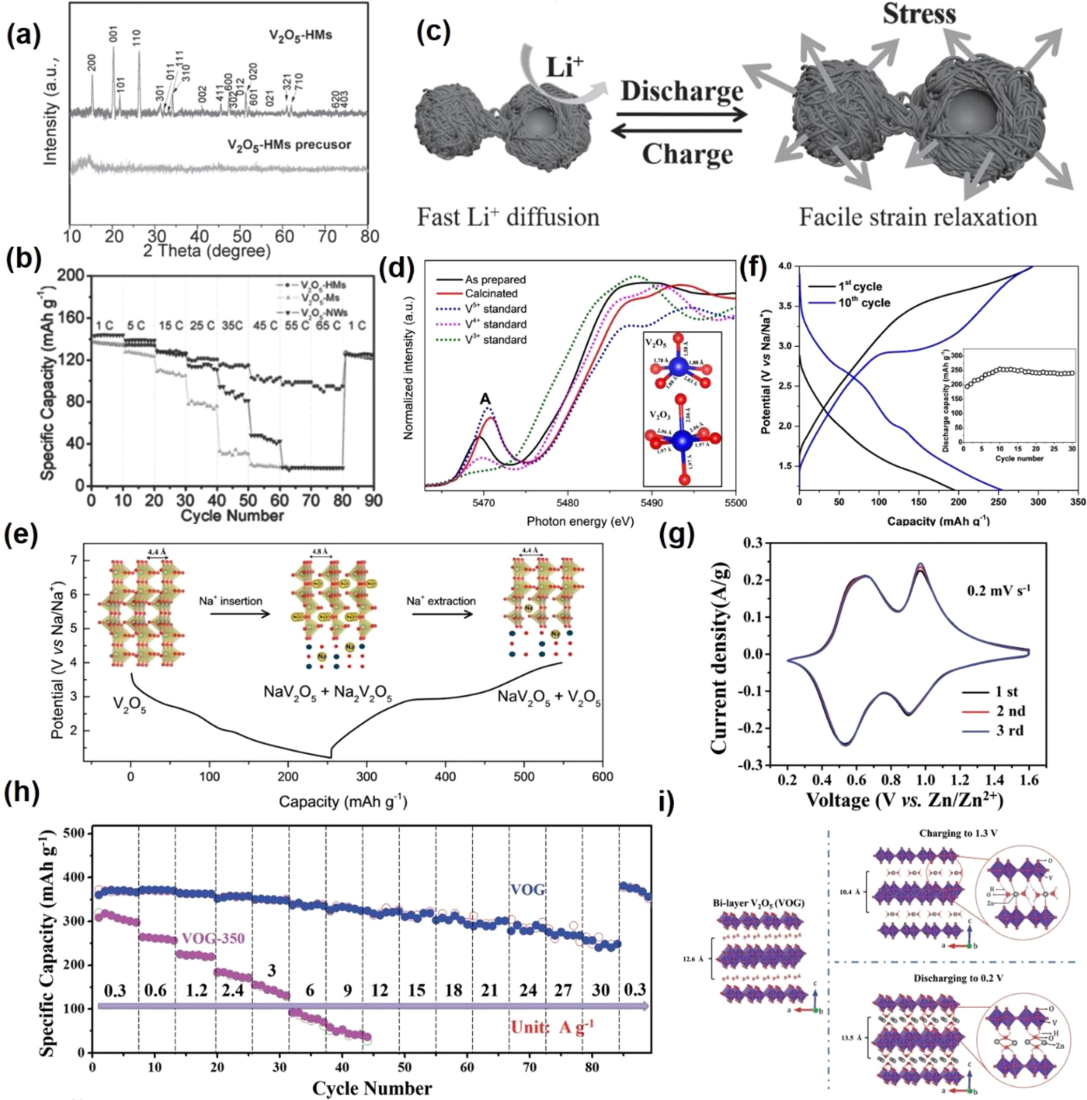
XRD patterns (a), rate performances (b) of V_2_O_5_–HMs, V_2_O_5_-Ms, and V_2_O_5_–NWs. Schematic illustration of the V_2_O_5_–HMs during charge/discharge process (c). Reproduced with permission from ref (308). Copyright 2016 John Wiley & Sons. The XANES spectra of as prepared and after calcination at 400 °C are plotted with solid lines (d), the Na^+^ de/intercalation channels crystal structure (e), charge/discharge profiles and cycling performance (f) of orthorhombic V_2_O_5_/C. Reproduced with permission from ref (309). Copyright 2016 American Chemical Society. The CV curves (g), rate performances (h), and the proposed crystal structures at different states (i) of V_2_O_5_·H_2_O/graphene. Reproduced with permission from ref (310). Copyright 2018 John Wiley & Sons.

V_2_O_5_ is a feasible cathode material for metal-ion storage due to its high output voltage and unique crystal structure with large interlayer spacing (4.4 Å). Mai et al.^308^ designed and synthesized a V_2_O_5_ hollow microclew (V_2_O_5_–HM) through a facile solvothermal assisted calcination method. The amorphous V_2_O_5_–HM precursor can convert into crystalline V_2_O_5_ through calcination (Figure 37a). Compared with crystalline V_2_O_5_ (V_2_O_5_-Ms) and V_2_O_5_ nanowires (V_2_O_5_–NWs), the V_2_O_5_–HMs exhibit the best Li^+^ storage performance (145.3 and 94.8 mAh g^–1^ at 0.67 and 65 C, respectively), which is due to the 3D hierarchical microstructure with intertangled nanowires (Figure 37b). This 3D hierarchical microstructure not only inherits fast electrolyte penetration, and short ionic and electronic transport pathways, but also significantly alleviates the strain during the Li^+^ intercalation/deintercalation. Meanwhile, compared to disordered V_2_O_5_ nanowires, a unique V_2_O_5_–HM structure effectively helps improve the tap density, which is more suitable for commercial applications (Figure 37c). V_2_O_5_ can be used as other metal ions (Na^+^/K^+^/Zn^2+^) electrode material except for LIB storage material. Chung group synthesized a nanosized V_2_O_5_/C composite cathode by ball milling the nanosized V_2_O_5_ with acetylene black and investigating the reaction mechanism in the NIB system.^309^ Generally, compared with other vanadium oxides (VO_2_, V_2_O_3_, et al.), V_2_O_5_ consists of the square-based pyramid with the highly distorted environment and exhibits the highest pre-edge intensity from the X-ray absorption near edge structure (XANES) result. Thus, the average vanadium oxidation state of the calcinated V_2_O_5_/C sample is +4.83 (Figure 37d), which may be due to the calcined carbon reduction (a slight difference with standard V_2_O_5_ in the pre-edge peak position). They used *ex-situ* XRD to demonstrate the major NaV_2_O_5_ with a minor Na_2_V_2_O_5_ phase formed at the first discharge state, accompanying a *c* lattice parameter increase by 9.09% and unit cell volume increase by 9.2%. At the subsequent charge, the NaV_2_O_5_ + Na_2_V_2_O_5_ will transform into NaV_2_O_5_ + V_2_O_5_ along with a V^4+^ → V^5+^ change (Figure 37e). The V_2_O_5_/C delivers an initial discharge capacity of 195 mAh g^–1^, and increases to 255 mAh g^–1^ at the 10th cycle corresponding to 1.7 Na^+^ inserts into per unit formula (Figure 37f). Besides LIB/NIB cathodes, V_2_O_5_ can be investigated as an aqueous zinc-ion battery (ZIB) cathode material, and the intercalation of water into the vanadium oxide can increase the interlayer distance, which is in favor of expanding the gallery for Zn^2+^ intercalation. Yang et al.^310^ reported V_2_O_5_·H_2_O/graphene (VOG) synthesized via a freeze-drying method. They investigated the critical role (“lubricating” effect) of structural H_2_O on the Zn^2+^ intercalation into bilayer V_2_O_5_·*n*H_2_O, and H_2_O-solvated Zn^2+^ possesses a largely reduced effective charge and improves electrochemical performance. VOG delivers a high capacity of 381 mAh g^–1^ at 60 mA g^–1^ and maintains 248 mAh g^–1^ at a high current density of 30 A g^–1^, which are much higher than those of most aqueous ZIB cathode materials (Figure 37g,h). The interlayer distances of VOG are 12.6, 13.5, and 10.4 Å at initial, discharge, and charge states by ex-situ XRD and MAS NMR (Figure 37i). These results demonstrate that water in vanadium oxide layers plays an important role in the performance of an aqueous ion battery.

**Figure 38:**
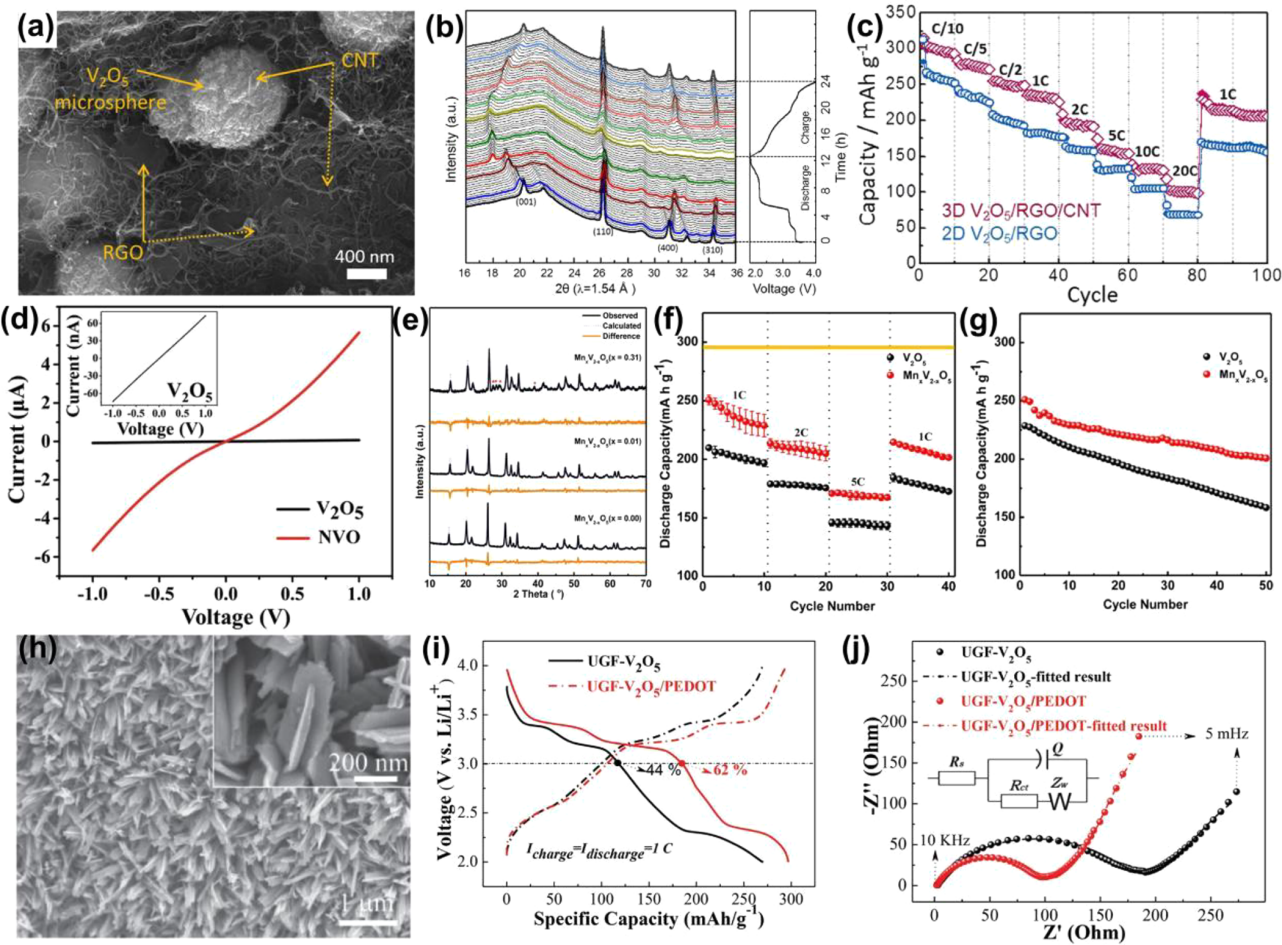
FESEM image (a), in situ XRD (b), and rate performance (c) of 3D V_2_O_5_/rGO/CNT composite. Reproduced with permission from ref (311). Copyright 2016 under CC BY 4.0 license. The *I*–*V* curves of Na_0.33_V_2_O_5_ (NVO) and V_2_O_5_ (d). Reproduced with permission from ref (312). Copyright 2018 John Wiley and Sons. Rietveld refined XRD patterns of the Mn_*x*_V_2–*x*_O_5_ compounds (e), rate performance (f), and cycle performance (g) at 300 mA g^–1^ of Mn_0.01_V_1.99_O_5_ and V_2_O_5_. Reproduced with permission from ref (313). Copyright 2015 American Chemical Society. The SEM image of UGF-V_2_O_5_/PEDOT (h), the charge/discharge profiles (i), Nyquist plots at fully charged stage (j) of UGF-V_2_O_5_/PEDOT and UGF-V_2_O_5_. Reproduced with permission from ref (314). Copyright 2014 John Wiley and Sons.

To investigate the electrical conductivity and structural mechanism during lithium insertion/deinsertion of V_2_O_5_, Yoon et al.^311^ developed a 3D V_2_O_5_/rGO/CNT with short Li^+^ diffusion, and high continuous 3D conductive network, and investigated its structural mechanism during Li^+^ intercalation/deintercalation by in situ XRD/XANES analysis (Figure 38a). The 3D V_2_O_5_/rGO/CNT delivers a high discharge capacity of 100 mAh g^–1^ at 20 C, which is much higher than 2D V_2_O_5_/rGO (68 mAh g^–1^). There are numerous metastable phases of Li_*x*_V_2_O_5_ during Li^+^ intercalation into V_2_O_5_. The α-Li_0.26_V_2_O_5_, ε-Li_0.93_V_2_O_5_, δ-Li_1.27_V_2_O_5_, γ-Li_1.93_V_2_O_5_, and ω-Li_2.65_V_2_O_5_ phase form in turn during the first discharge to 3.4, 3.3, 3.19, 2.28, and 2.01 V, respectively. In addition, the most reflection will return to the same position at a pristine state during the subsequent charge process, which can confirm the high structural reversibility of V_2_O_5_ in the ternary composite upon Li^+^ intercalation/deintercalation (Figure 38b). The 3D V_2_O_5_/rGO/CNT delivers a high discharge capacity of 100 mAh g^–1^ at 20 C, which is higher than 2D V_2_O_5_/rGO (68 mAh g^–1^) (Figure 38c). The preintercalation interlayer metal ions can act as pillars to increase the electronic conductivity, ion diffusion rate, and stability of layered vanadium oxides. Mai et al.^312^ designed and assembled Na_0.33_V_2_O_5_ (NVO) and V_2_O_5_ single nanowire devices to investigate the effect on the intrinsic electrical conductivity of Na^+^ intercalation. The conductivity of NVO is 5.9 × 10^4^ S m^–1^, while the conductivity of V_2_O_5_ is 7.3 S m^–1^, which indicates that the electronic conductivity of V_2_O_5_ is greatly improved by the Na^+^ intercalation (Figure 38d). Stucky et al.^313^ fabricated a series of nanostructured Mn-doped V_2_O_5_ cathode materials and found that the larger Mn doping in the modified V_2_O_5_ structure can increase the cell volume, which facilitates high Li^+^ diffusion and improves the electronic conductivity (Figure 38e). The Mn_0.01_V_1.99_O_5_ delivers a high discharge capacity (251 mAh g^–1^ at 1 C) and excellent cycling stability (80% after 50 cycles), which is much higher than V_2_O_5_ (215 mAh g^–1^ vs. 70%) (Figure 38f,g). Fan et al.^314^ reported lightweight, freestanding V_2_O_5_ nanoarray-based positive electrodes (UGF-V_2_O_5_/PEDOT), which were prepared by growing a V_2_O_5_ nanobelt array directly on 3D ultrathin graphite foam (UGF), followed by coating the V_2_O_5_ with a mesoporous thin layer of the conducting polymer poly(3,4-ethylenedioxythiophene) (PEDOT) (Figure 38h). In addition, the PEDOT coating constructs an integrated conductive network for the V_2_O_5_, providing decreased electrode polarization, improved charge transfer kinetics, and a prolonged discharge plateau of V_2_O_5_ (Figure 38i,j), and therefore it can lead to an increased proportion of high-voltage capacity and energy density than that without PEDOT.

#### 7.2.2 Supercapacitors

**Figure 39:**
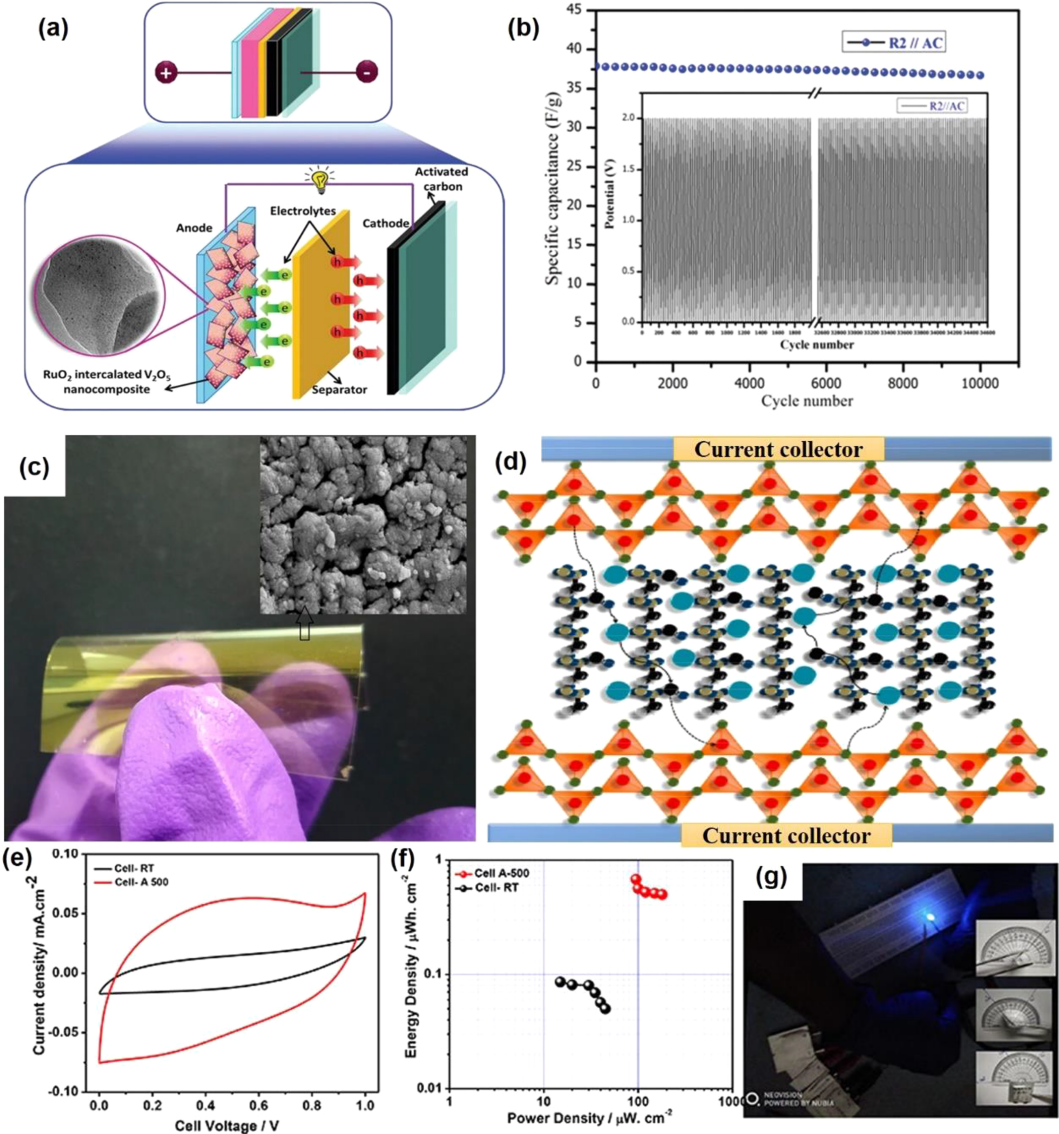
(a) Schematic illustration of the 3 wt % RuO_2_ in V_2_O_5_ asymmetric supercapacitor device and (b) cyclic stability 3 A g^–1^. Reproduced with permission from ref (315). Copyright 2021 Royal Society of Chemistry. (c) As-fabricated V_2_O_5_ flexible thin film, and the inset view is the FESEM image of the film. (d) Schematic representation of the symmetric V_2_O_5_ capacitor. (e) Comparison of CV curves of the symmetric SCs with V_2_O_5_ electrode prepared at room temperature (RT) and 50 °C (A-500) at a scan rate of 50 mV s^–1^. (f) Ragone plot comparison of both symmetric-RT and symmetric A-500 capacitors. (g) Lighting of LED using symmetric A-500 connected in series (inset: various bent position of the symmetric A-500). Reproduced with permission from ref (306). Copyright 2019 American Chemical Society.

Among all types of vanadium oxides, V_2_O_5_ has attracted attention for the application of SCs due to its broad oxidation states, high specific capacitance, and low acquisition cost. Palani et al. fabricated RuO_2_ nanoparticle-decorated V_2_O_5_ nanoflakes by a solvothermal method.^315^Figure 39a illustrates the corresponding schematic of the fabricated asymmetric cell that exhibited a high specific capacitance of 421 F g^–1^ at a current density of 1 A g^–1^ with excellent cyclic retention of 94.6% over 10000 cycles (Figure 39b). The symmetric device of V_2_O_5_||PVA-KOH||V_2_O_5_ was fabricated using thin flexible substrate by Velmurugan et al. (Figure 39c), where both annealed (A-500) and as-prepared (RT) V_2_O_5_ films were used as electrode material separately.^306^ A schematic representation of the structural image of the V_2_O_5_ is provided in Figure 39d. The CV curves in Figure 39e denote that the symmetric A-500 gives a larger area under the curve than the symmetric-RT, suggesting the improved performance of the annealed sample. The A-500 devices display a maximum energy density of 0.68 μWh cm^–2^, which is obviously much higher than that of the RT electrodes (0.05 μWh cm^–2^, Figure 39f). Moreover, the symmetric A-500 shows excellent cycle life up to 30000 cycles with a Coulombic efficiency of 99%. As shown in Figure 39g, the practical feasibility of the as-fabricated devices was demonstrated by lighting blue light-emitting diodes. Several groups reported the hybrid structure of V_2_O_5_ with carbon materials fabricated by different strategies for enhancing the SC property. For example, Sahu et al.^316^ synthesized graphene nanoribbon @V_2_O_5_ nanostrip composites to improve the conductive property of V_2_O_5_, which displays a high energy density of 42.09 Wh kg^–1^ and power density of 475 W kg^–1^. W. Sun et al.^317^ prepared a 3D monolithic aerogel composed of uniform carbon nanofibers/V_2_O_5_ core/shell nanostructures. The composite aerogel exhibits high specific capacitance (595.1 F g^–1^), excellent energy density (82.65 Wh kg^–1^), and good cycling behavior (>12000). Zhu et al. proposed a simple “liquid phase impregnation template” strategy to successfully synthesize hierarchically porous V_2_O_5_/C nanocomposites that exhibits a specific capacitance of 492.1 F g^–1^, as well as an energy density of 87.6 Wh kg^–1^.^318^ Yao et al. successfully synthesized SCs based on 3D networks hybrids of reduced graphene oxide and V_2_O_5_ nanobelts through a simple hydrothermal method.^319^ The rGO/V_2_O_5_ hybrid aerogel electrodes showed a high energy density of 249.7 W kg^–1^ and excellent long-term cycle stability (remaining 90.2% after 5000 cycles).

#### 7.2.3 Catalysts

##### 7.2.3.1 OER

**Figure 40:**
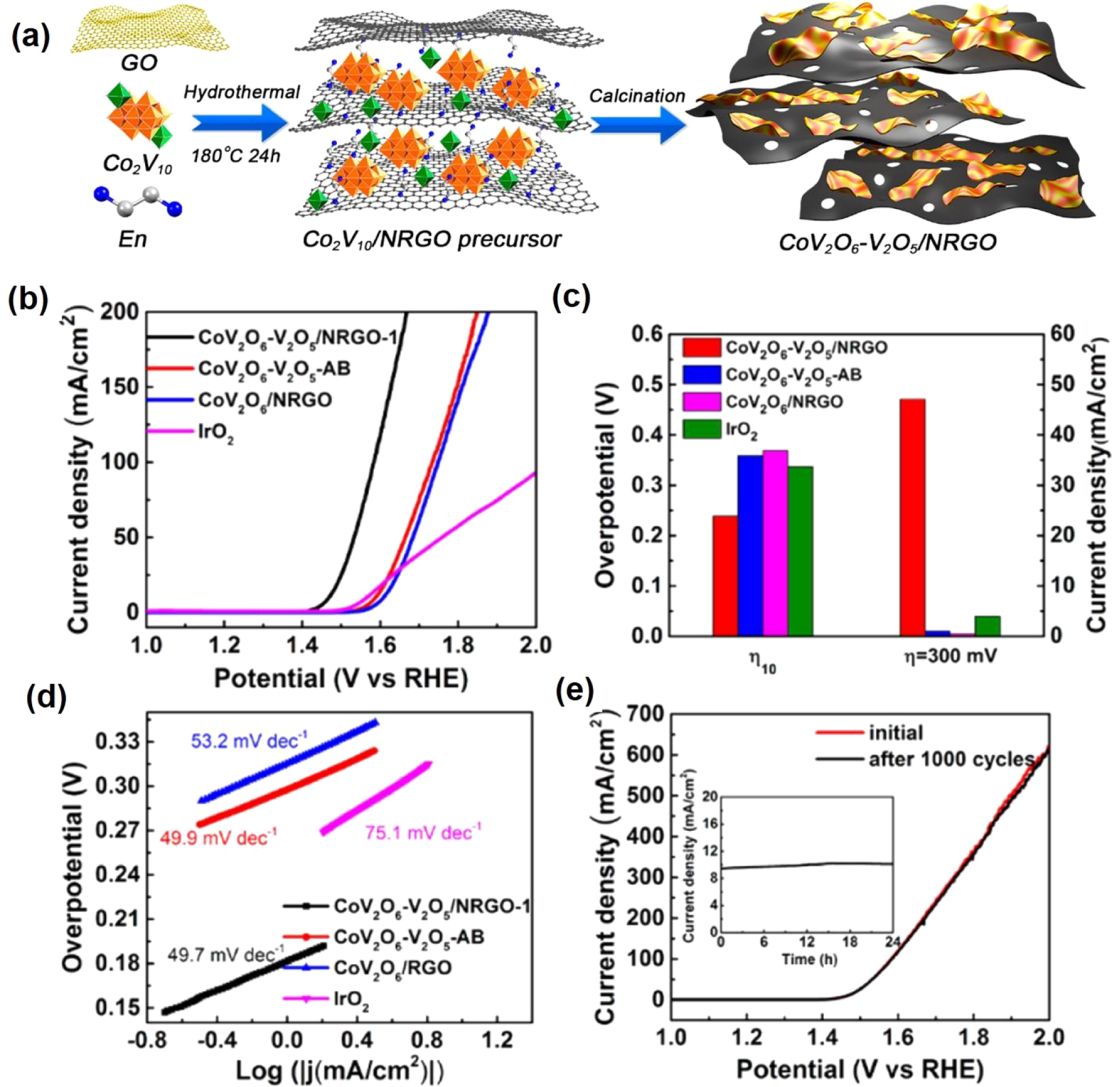
(a) Schematic illustration of the synthetic process of CoV_2_O_6_–V_2_O_5_/NRGO composite, (b) polarization curves, (c) comparison of catalysts’ overpotential at a current density of 10 mA cm^–2^, corresponding current density at an overpotential of 300 mV, and (d) corresponding Tafel plots. (e) Initial polarization curves of CoV_2_O_6_–V_2_O_5_/NRGO and after 1000 CV cycles. Inset: time-dependent current density curve of CoV_2_O_6_–V_2_O_5_/NRGO under a potential of 239 mV for 24 h. Reproduced with permission from ref (320). Copyright 2017 American Chemical Society.

V_2_O_5_ exhibited good OER performance due to the multivalent states of the V element, which can enrich active intermediates (*OH, *O, and *OOH) by regulating the valence electron structure of the V element.^275,320^ The OER performance could be enhanced by fabricating the composites with other materials. Lan et al.^320^ synthesized CoV_2_O_6_-V_2_O_5_/nitrogen-doped reduced graphene oxide composites (CoV_2_O_6_-V_2_O_5_/NRGO) by a one-pot hydrothermal method integrating polyoxovanadate, ethylenediamine (EN), and graphene oxide (GO) for the precursor and postcalcined process (Figure 40a). Without V_2_O_5_, the CoV_2_O_6_/NRGO delivered a relatively acceptable OER performance with an overpotential of 379 mV at a current density of 10 mA cm^–2^, which is comparable to that of IrO_2_ (337 mV). By adding V_2_O_5_, the OER performance could be enhanced with an overpotential of 239 mV at a current density of 10 mA cm^–2^ (Figure 40b). Furthermore, the CoV_2_O_6_–V_2_O_5_/NRGO exhibits a higher current density (47.08 mA cm^–2^) at an overpotential of 300 mV compared with CoV_2_O_6_/NRGO (0.45 mA cm^–2^) and IrO_2_ (3.95 mA cm^–2^) (Figure 40c). Meanwhile, the CoV_2_O_6_–V_2_O_5_/NRGO shows the fastest reaction kinetics with a Tafel slope of 49.7 mV dec^–1^, which could be due to the enhanced charge transport (Figure 40d). The CoV_2_O_6_–V_2_O_5_/NRGO gives good stability from the polarization curves, which are almost overlapping before and after 1000 cycles (Figure 40e). The theoretical calculation found that the existence of the hydrogen bond between V_2_O_5_ and intermediate HOO* of OER decreases the adsorption energy, which may be responsible for the low overpotential.

##### 7.2.3.2 HER

**Figure 41:**
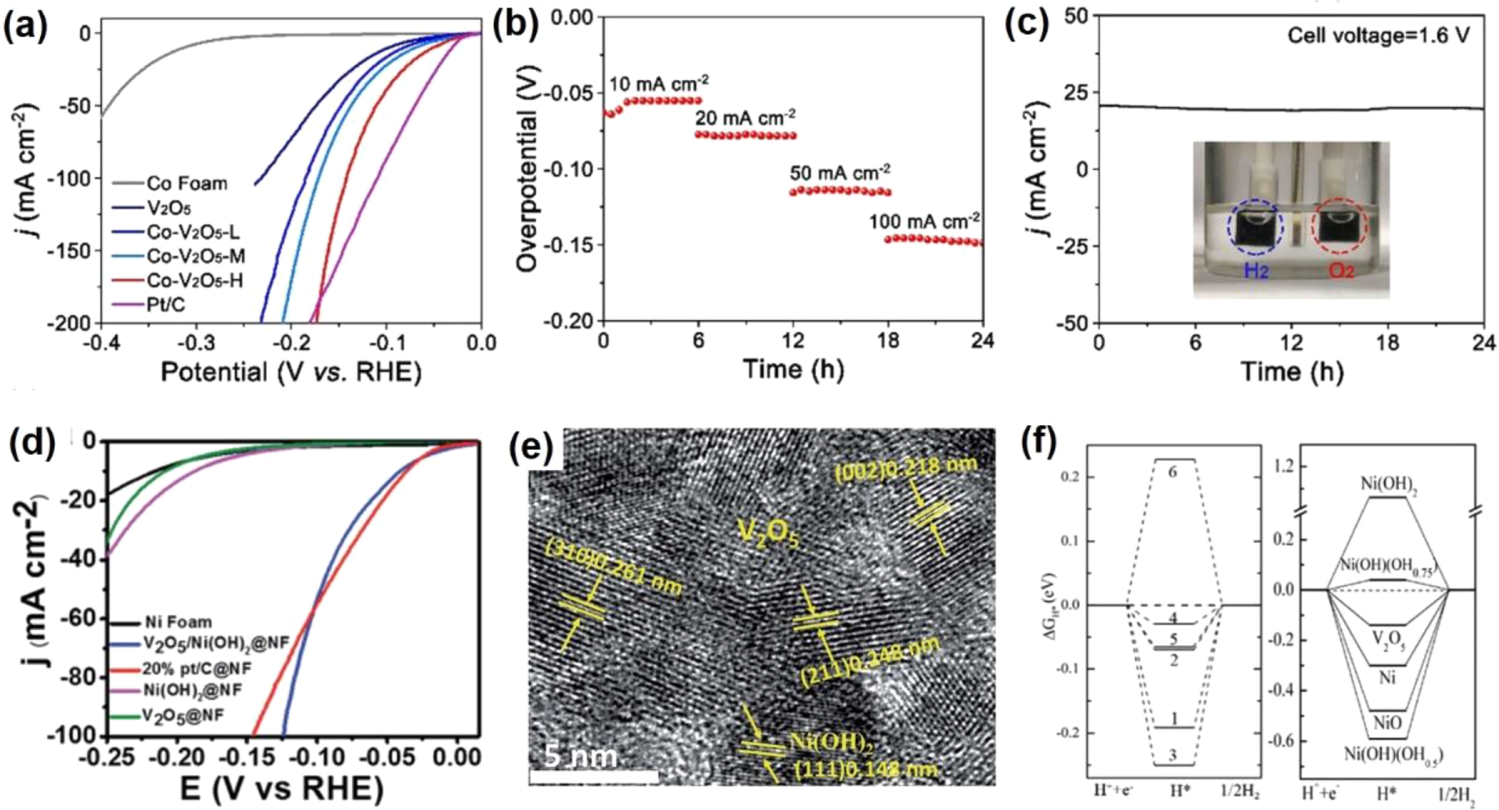
(a) HER polarization curves of the Co–V_2_O_5_–H sample, (b) stability of the Co–V_2_O_5_–H at 10–100 mA cm^–2^, (c) stability of the Co–V_2_O_5_–H // Co–V_2_O_5_–H couple at 1.6 V for overall water splitting. Reproduced with permission from ref (328). Copyright 2020 Elsevier. (d) Polarization curves of NF, 20% Pt/C@NF, V_2_O_5_@NF, Ni(OH)_2_@NF, and V_2_O_5_/Ni(OH)_2_@NF at a scan rate of 2 mV s^–1^, (e) HRTEM image of V_2_O_5_/Ni(OH)_2_@NF, (f) calculated free energies at sites 1 to 6 (1: the reconstructed Ni-surface, 2: the Ni-site of Ni(OH)_2_@Ni, 3: the Ni-site between V_2_O_5_@Ni and Ni(OH)_2_@Ni, 4: the Ni-site of V_2_O_5_@Ni, 5: the O-site on the V_2_O_5_ surface, 6: the O-site inside the V_2_O_5_ channel) and various individual pristine materials. Reproduced with permission from ref (329). Copyright 2019 Royal Society of Chemistry.

In general, V_2_O_5_ is generally considered as an HER-inactive material due to the weak H* adsorption on V sites, which limits the formation of H* at the active site.^321,322^ Three main methods can be adopted to promote the HER performance of V_2_O_5_: defects creation, interfacial engineering, and forming composites. First of all, creating defects, especially vacancies, is regarded as an efficient way to enhance the HER performance.^323−325^ Oxygen vacancy (V_O_) in transition metal oxides can accelerate the adsorption of the H* intermediates by activating the delocalized electrons of the metal center, which could lead to a better HER performance.^326,327^ Li et al.^328^ synthesized the V_2_O_5_ nanosheet arrays with V_O_ on Co foam through a hydrothermal reaction. The highest V_O_ concentration of 34.2% of the V_2_O_5_ nanosheet arrays on the Co foam (Co–V_2_O_5_–H) is easily obtained by controlling the pH = 1 of the NH_4_VO_3_ precursor solution. The Co–V_2_O_5_–H exhibits a low overpotential of 51 mV at a current density of 10 mA cm^–2^, which shows better performance compared to the V_2_O_5_ powder and low V_O_ concentration samples (Figure 41a). Meanwhile, the catalyst shows a negligible potential drop at different current densities, indicating long-time stability (Figure 41b). The Co–V_2_O_5_–H is employed as both a cathode and anode to establish a two-electrode alkaline electrolyzer for overall water splitting, which can maintain a steady output current at a cell voltage of 1.6 V for 24 h (Figure 41c). Second, the interfacial engineering between V_2_O_5_ and transition metal also provides a route to improve the HER performance. Kim et al.^329^ directly grew the V_2_O_5_ particles on Ni foam via a one-step hydrothermal method. The as-prepared V_2_O_5_/Ni(OH)_2_@NF catalyst shows a low overpotential of 39 mV at a current density of 10 mA cm^–2^, which is comparable to Pt (35 mV @ 10 mA cm^–2^). The Ni(OH)_2_@NF samples show an overpotential of 188 mV at 10 mA cm^–2^, which indicates that the V_2_O_5_ plays an important role in the HER performance (Figure 41d). DFT calculation was performed to investigate the active sites of the V_2_O_5_/Ni(OH)_2_@NF catalyst. The exposed facet of V_2_O_5_ is the (010), (001), and (310) planes, which is confirmed by TEM (Figure 41e). The H adsorption energy of the V_2_O_5_ (001) and (310) surfaces is larger than that of V_2_O_5_ (010), which indicates that V_2_O_5_ (010) is the active surface. Furthermore, the Δ*G*_H*_ values of the Ni-sites at the edges of interfaces in Ni(OH)_2_@Ni and V_2_O_5_@Ni and the O-site of V_2_O_5_ (corresponding to 2, 4, and 5, respectively, as shown in Figure 41f) are close to zero, which is nearly equal to that of the Pt(111)-surface. The calculation indicated the edges of the interfaces in V_2_O_5_/Ni(OH)_2_@NF play a significant role in the HER process. Moreover, the hierarchical V_2_O_5_@Ni_3_S_2_ hybrid nanoarray also exhibited a good overpotential of 95 mV at 10 mA cm^–2^,^330^ which is also due to the interfaces between V_2_O_5_ and Ni_3_S_2_. Third, V_2_O_5_ is also used as an additive with other materials to form a composite and further enhance the HER performance. By doping phosphorus and adding V_2_O_5_ into Pt/graphene, the prepared catalysts exhibit a good HER performance of the initial potential of 32 mV and a Tafel slope of 23 mV dec^–1^.^331^

##### 7.2.3.3 Photocatalysis

**Figure 42:**
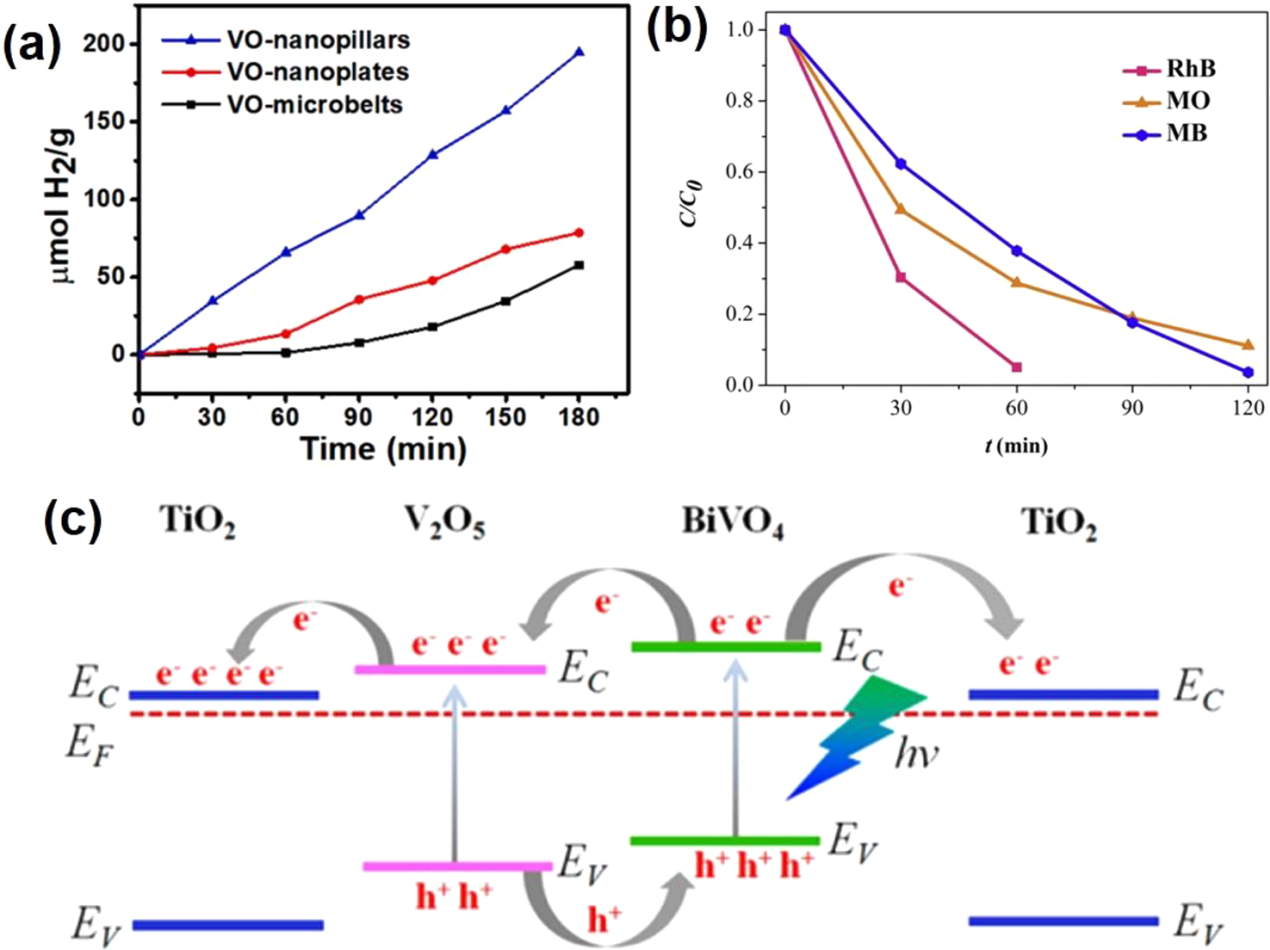
(a) The hydrogen generation curves for VO-microbelts, VO-nanoplates, and VO-nanopillars with a sacrificial agent. Reproduced with permission from ref (335). Copyright 2021 Elsevier. (b) The photodegradation performance of g-C_3_N_4_/V_2_O_5_ photocatalyst for RhB, MO, and MB degradation under visible light irradiation. Reproduced with permission from ref (344). Copyright 2016 Elsevier. (c) Schematic diagram of lectron–hole pairs separation of the V_2_O_5_/BiVO_4_/TiO_2_ nanocomposites under visible-light irradiation. Reproduced with permission from ref (349). Copyright 2014 American Chemical Society.

It is well documented that V_2_O_5_ has a typical narrow band gap (∼2.3 eV) and wide optical absorption range and is a high electron mobility semiconductor, which exhibits good photoresponsive properties by capturing visible light and is widely used in the electro-photocatalytic field, such as for hydrogen production, environmental pollutant degradation, etc.^332−334^ Garcia et al.^335^ found that the morphology of V_2_O_5_ nano-/microparticles dramatically affected the hydrogen production by photocatalysis. The V_2_O_5_ with microbelts, nanoplates, and nanopillars morphology can be obtained by using sunflowers’ petals and the center of the sunflower as biodegradable templates during the synthesis.^335^ The nanopillar V_2_O_5_ delivers a maximum hydrogen generation rate in the presence of Na_2_SO_3_ as a sacrificial agent with 65.5 μmol g^–1^ h^–1^, which is higher than microbelts (26.3 μmol g^–1^ h^–1^) and nanoplates V_2_O_5_ (19.4 μmol g^–1^ h^–1^) (Figure 42a). The high hydrogen generation rate is attributed to the large surface area, high absorbance in the UV–vis range, high photocurrent, and high content of defects of the V_2_O_5_ nanopillars. By fabricating the nanocomposite with V_2_O_5_ containing 1 or 2 heterojunctions, the hydrogen generation rate will be further improved, which is due to the delayed electron–hole recombination.^335^ For example, graphitic carbon nitride nanosheets/V_2_O_5_ composites (578 μmol g^–1^ h^–1^),^336^ Na_2_Ti_3_O_7_/V_2_O_5_/g-C_3_N_4_ composites (11000 μmol g^–1^ h^–1^),^337^ Na_2_TiO_3_/V_2_O_5_/g-C_3_N_4_ composites (567 μmol g^–1^ h^–1^),^337^ and Nb-doped SnO_2_/V_2_O_5_ (1346 μmol g^–1^ h^–1^).^338^ Transition metal oxides (TiO_2_, ZnO, etc.) have been applied in the removal of environmental pollutants in water, which could completely decompose organic pollutants into CO_2_ and H_2_O by photocatalytic oxidation.^339,340^ Hollow V_2_O_5_ microspheres consisting of randomly packed platelets showed an enhanced UV light absorption compared to the commercial V_2_O_5_ powder, which led to the highest activity for degrading rhodamine B under UV light.^341^ Composites, consisting of 1D V_2_O_5_ nanorod and 2D carbon-based materials, are promising photocatalysts for environmental pollutant degradation. The V_2_O_5_ nanorods/graphene oxide and V_2_O_5_ nanorods/graphene nanocomposites showed good degradation performance of Victoria blue dye and methylene blue dye (>95% degradation within 90 min), respectively.^342,343^ Especially, the nanocomposites exhibited the best degradation performance under direct sunlight irradiation compared to UV and visible light. The g-C_3_N_4_ is also used to construct the heterojunctions with V_2_O_5_ for a high-performance degradation catalyst.^344^ The photoexcited electron in the conduction band of g-C_3_N_4_ shows a strong reducing ability, while the photoexcited hole on the valence band of V_2_O_5_ exhibits a strong oxidizing ability. Thus, the g-C_3_N_4_ /V_2_O_5_ heterojunctions exhibited efficient degradation performance of rhodamine B, methyl orange (MO), and methylene blue (MB) dyes under visible light (Figure 42b). Besides the graphene or g-C_3_N_4_, the V_2_O_5_ composites with other inorganic photocatalytic oxides also showed enhanced photocatalytic properties for degradation of organic pollutants, such as V_2_O_5_/BiVO_4_,^332,345^ V_2_O_5_/CeO_2_,^346^ V_2_O_5_/TiO_2_,^347,348^ and so on. The V_2_O_5_-based composite can degrade not only the dye molecules in solution, but also some small solvent molecules in the gas phase. The ternary V_2_O_5_/BiVO_4_/TiO_2_ nanocomposites exhibited a well-aligned band structure and increasing photoinduced charge carriers through the charges separation across their multiple interfaces, which resulted in good light absorption from the UV to the visible region and better photocatalytic activity for the decomposition of gaseous toluene compared to pure TiO_2_ and V_2_O_5_/BiVO_4_ under visible light irradiation (Figure 42c).^349^

#### 7.2.4 Electrochromism

Since Colton et al. did the pioneering work on the electrochromism of V_2_O_5_ in 1976–1977,^350,351^ V_2_O_5_ has attracted increasing attention among transition metal oxides because it can exhibit both anodic and cathodic coloration.^352^ From 1975 to 1999, studies focused on the absorption-transmission spectra modulation in visible and infrared light, while, after 2000, V_2_O_5_ with various micro- and nanostructures were emerging, and researchers paid attention to improve some important key figures of merit to evaluate the electrochromic performance, such as the coloration efficiency, cycling life, switching time, and so on.^353−358^ Recently, several reviews were published on the electrochromic application of V_2_O_5_ film, which provide more specific and detailed information on this topic.^256,352,359,360^ For V_2_O_5_, the discoloration mechanism is explained as the result of injection/extraction of electrons and electrolyte cations and variation of valence change of vanadium ions, which is widely accepted.^361,362^

**Figure 43:**
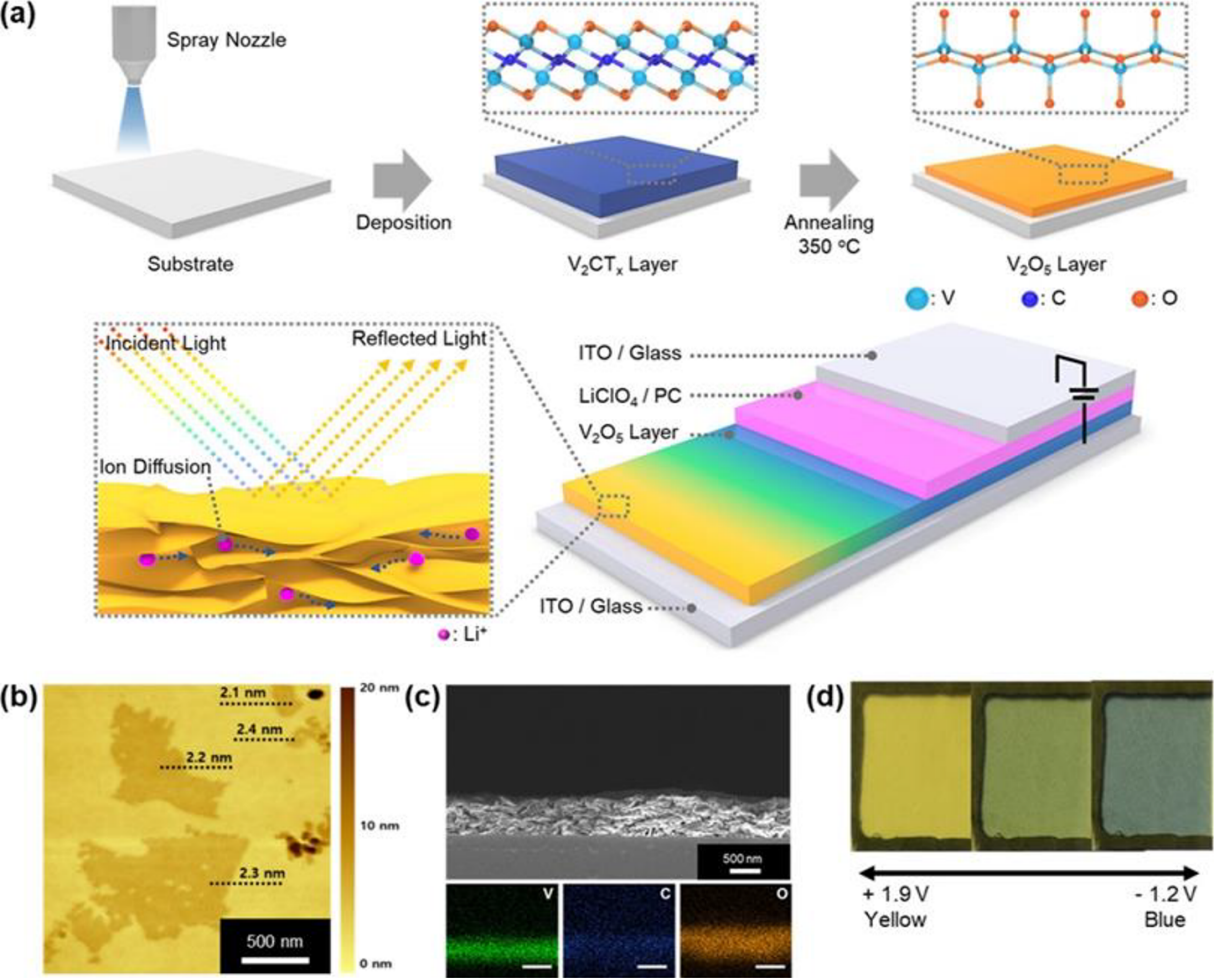
Fabrication of a 2D V_2_O_5_ nanosheet based electrochromic device. (a) Schematic for fabrication of an electrochromic device based on a V_2_CT_*x*_-derived 2D V_2_O_5_ nanosheet, resulting in minimized optical scattering and enhanced ion diffusion. (b) Representative AFM image of single layer V_2_CT_*x*_ with a submicron lateral size. (c) SEM image of cross-section of 2D V_2_O_5_ nanosheet based electrochromic layer and corresponding EDS analysis in terms of vanadium, carbon, and oxygen. (d) Digital image of electrochromic device operating from +1.9 V to −1.2 V. Reproduced with permission from ref (366). Copyright 2023 Elsevier.

In order to improve the performance of V_2_O_5_ as an electrochromic device, many strategies have been designed. Especially, V_2_O_5_ nanostructures with small sizes and large specific surface areas are expected to facilitate the ion intercalation/deintercalation process, thereby enhancing the electrochromic properties. Panagopoulou et al.^363^ successfully prepared Mg-doped V_2_O_5_ thin films using RF sputtering, and found the 15 atom % Mg-doped films displayed optimal electrochromic properties with the fastest switching time of *t*_c_ = 10/4 s (intercalation/deintercalation), the best coloration efficiency of 71.3 cm^2^ C^1–^ at 560 nm, higher visible transmittance of 85%, and the highest contrast value between the coloration states of Δ*T* (34.4% @ 560 nm). Qi et al.^364^ fabricated flexible V_2_O_5_ nanosheets/graphene oxide films, which exhibit ultrafast coloring response time (1.6 s) and bleaching time (2 s) attributed to the reduced charge transport distances of the ultrathin nanosheet structure (4–40 nm). They also display an excellent transmittance contrast of 57.5% at 425 nm and reversible yellow/green/blue-gray multicolor changes. Tong et al.^365^ fabricated a 3D crystalline V_2_O_5_ nanorod architecture on ITO substrates by a colloidal crystal-assisted electrodeposition method. Such architecture exhibits a highly reversible Li-ion insertion/extraction process (Columbic efficiency up to 96.9%), five distinct color change, good transmittance modulation Δ*T* (38.48% @ 460 nm), and acceptable response times (8.8 s for coloration and 9.3 s for bleaching), making it a promising film electrode for electrochromic devices. Kim et al.^366^ prepared highly crystalline 2D V_2_O_5_ nanosheets by using single-layer V_2_CT_*x*_ film as a sacrifice template (Figure 43a). The mean thickness and lateral size of V_2_CT_*x*_ are 2.38 nm and 0.78 μm, respectively (Figure 43b). Figure 43c shows the SEM image of V_2_O_5_ nanosheets film after annealing of V_2_CT_*x*_ at 350 °C. The 2D V_2_O_5_ nanosheets based electrochromic device has sharp multicolor transformations with a robust optical contrast from yellow to green to blue (Figure 43d). The corresponding optimal electrochromic performance shows a high optical contrast (53.98% @ 700 nm) and a fast response time (6.5 s for coloration and 5.0 s for bleaching).

## 8 Other Vanadium Oxides

### 8.1 V_2_O_2_

**Figure 44:**
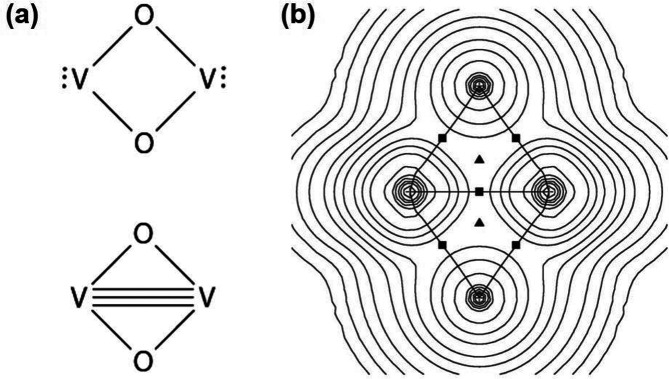
(a) Two possible Lewis representations highlighting the unclear bonding situation in V_2_O_2_. (b) Plot of the electron density within the molecular plane. Rectangles: bond critical points; triangles: ring critical points. Reproduced with permission from ref (368). Copyright 2017 John Wiley and Sons.

V_2_O_2_ is generally used as a tool to investigate the nature of metal–oxygen bonds, which is crucial to provide a proper rationalization of the relationship between structure and properties at an atomic scale.^367^ Stable V_2_O_2_ could not be synthesized by a traditional solid-state reaction or solution methods. However, it can be observed by IR spectroscopy on V, Ne, and O_2_ codeposited matrices.^368^ The calculation results show two possible vanadium bonding situations: 1) no bonds between the two vanadium atoms; 2) a short distance between the two vanadium atoms, which indicated multiple bonding (Figure 44a).^368−370^ However, Himmel et al.^368^ found that there is a multiple vanadium–vanadium bond in V_2_O_2_ molecules. There are three V–V bonding and antibonding orbitals, which are occupied by 1.70, 1.58, 1.50 electrons and 0.42, 0.40, 0.49 electrons, respectively. Thus, 4.78 electrons are in V–V bonding orbitals, and 1.31 electrons are in V–V antibonding orbitals. Furthermore, five bond critical points (four points between the oxygen and the vanadium atoms and one point in the center between the vanadium atoms) and two ring critical points (between the center of the cluster and the oxygen atoms) can be identified in V_2_O_2_ molecules (Figure 44b), which confirms the presence of a V–V bond. The presence of the strong V–V bond may lead to unique optical and magnetic properties.

### 8.2 V_4_O_9_

**Figure 45:**
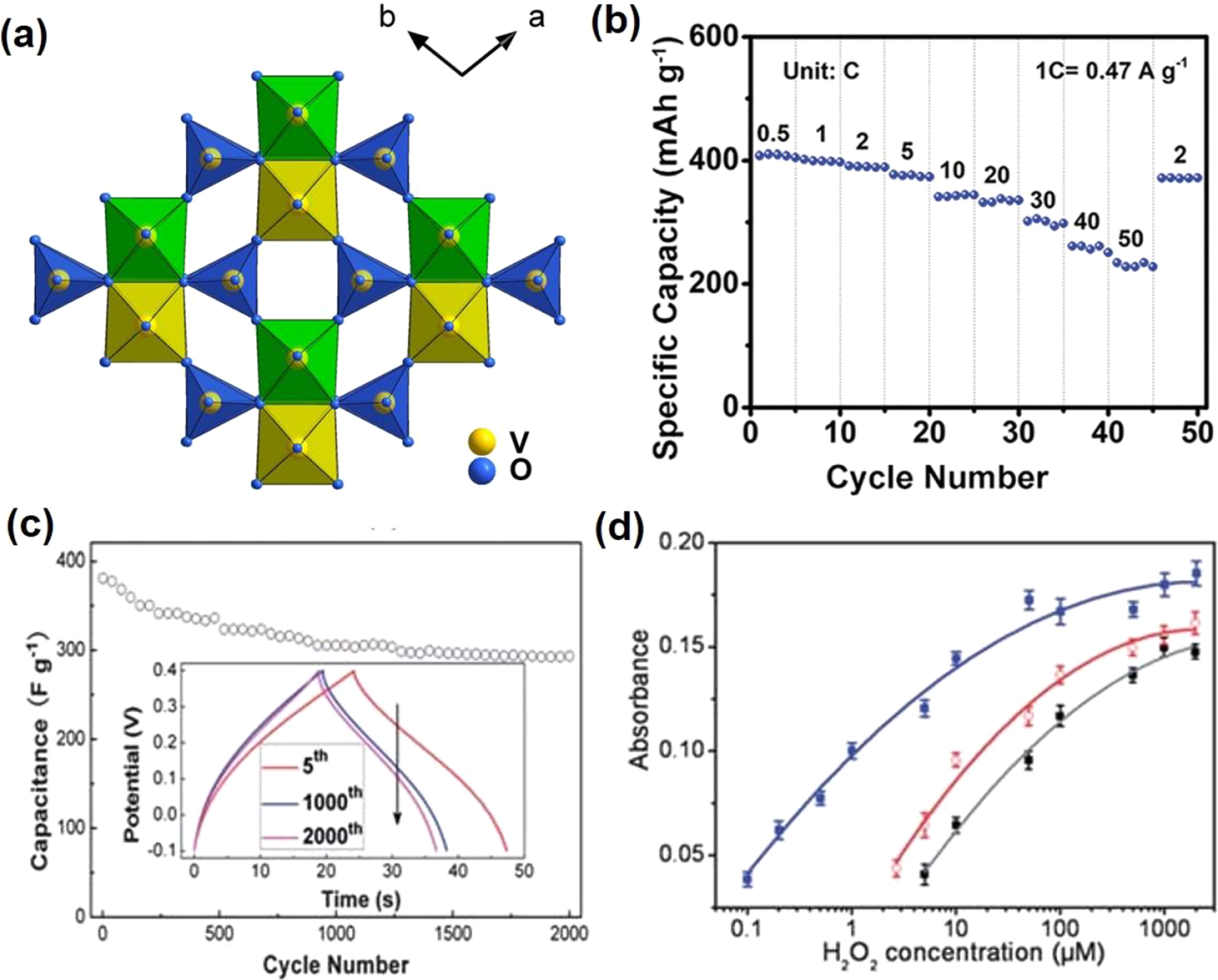
(a) Crystal structure of V_4_O_9_ with VO_6_ octahedron (yellow), VO_5_ pyramid (green), and VO_4_ tetrahedron (blue). (b) Rate performance at various currents ranging from 0.5 to 50 C of the aqueous Zn//V_4_O_9_ second battery. Reproduced with permission from ref (375). Copyright 2021 Royal Society of Chemistry. (c) The cycling stability of the specific capacitance of flower V_4_O_9_ at 2 A g^–1^. The inset shows the charge–discharge curves at different cycle numbers. (d) UV–vis absorbance curve (with polynomial fitting) of 75 mg flower V_4_O_9_ (blue) at varying H_2_O_2_ concentrations. Reproduced with permission from ref (373). Copyright 2013 Royal Society of Chemistry.

V_4_O_9_ has an orthorhombic structure with a space group of *Cmcm*, which processes three types of VO polyhedra (Figure 45a). The VO_5_ pyramids and VO_6_ octahedra make pairs, which are connected by corner oxygen atoms from the VO_4_ tetrahedra.^371^ The valence of the vanadium ion in the octahedron and pyramid is +4, while that in the tetrahedron is +5. V_4_O_9_ is difficult to synthesize by a solid-state reaction from the mixture of binary V_2_O_5_ and V_2_O_3_ (or VO_2_), but it can be synthesized by the reduction of V_2_O_5_ using reducing agents of carbon, SO_2_, and sulfur.^371,372^ The amount of reducing agents dramatically affects the final products, which may consist of other vanadium oxides, such as V_6_O_13_ and VO_2_. Consequently, reducing V_2_O_5_ by the solvothermal method is a facile way to obtain V_4_O_9_. Different solvents (tetraethylene glycol, 2-propanol, and tetrahydrofuran) are used to synthesize V_4_O_9_ with different morphologies, such as nanoflakes, nanosheets, and so on.^373−375^ Liang et al.^375^ investigated the aqueous zinc ion batteries of V_4_O_9_. It is found that the V_4_O_9_ exhibits fast zinc ion and electron diffusion, which is due to the unique tunnel structure and the V^5+^/V^4+^ mixed-valences induced metallic behavior. The V_4_O_9_ cathode shows a high reversible discharge capacity (420 mA h g^–1^ at 0.5 C). Even at a high current density of 50 C, it also exhibits an impressive discharge capacity of 234.4 mA h g^–1^, suggesting a fast Zn^2+^ storage ability of V_4_O_9_ (Figure 45b). Furthermore, V_4_O_9_ delivers an energy density of 175.8 W h kg^–1^ at a high power of 17625 W kg^–1^, which gives a high power density compared with other vanadium-based cathode materials in aqueous zinc ion batteries, such as V_2_O_5_,^376^ V_6_O_13_,^377^ LiV_3_O_8_,^378^ Na_3_V_2_(PO_4_)_3_,^379^ VO_2_,^380^ and VS_2_.^381^

The 2D single layered V_4_O_9_ nanosheet assembled 3D microflowers exhibit good supercapacitor performance with a specific capacitance of 392 F g^–1^ at a current density of 0.5 A g^–1^ and 75% retained capacitance after 2000 cycles (Figure 45c).^373^ The flower-like structure assembled from ultrathin and well-separated nanosheets was unchanged during the charge–discharge cycles, which is responsible for the high capacitance and stability. Meanwhile, the V_4_O_9_ flower demonstrates a good ability to sense H_2_O_2_ and methanol with a detection limit of ∼0.1 μM and ∼60 μM, respectively (Figure 45d).

### 8.3 V_6_O_13_

**Figure 46:**
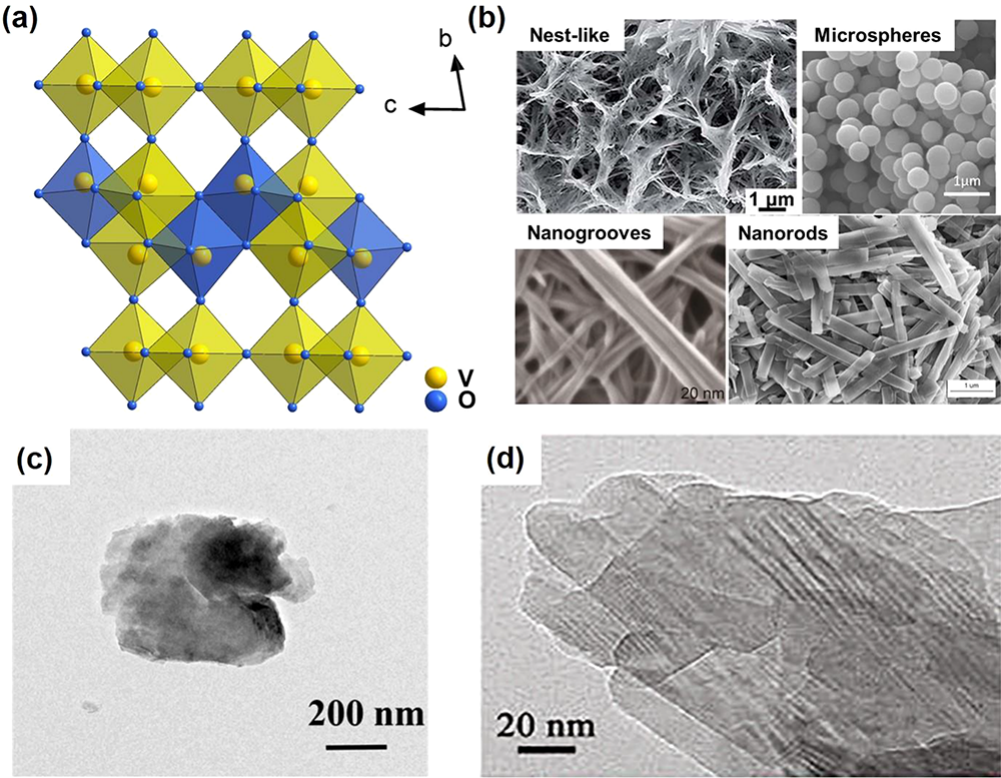
(a) Crystal structure of V_6_O_13_ with V two types of VO_6_ octahedra. (b) Different morphologies of V_6_O_13_ synthesized by a solvothermal method. Reproduced with permission from ref (385) (Copyright 2020 Royal Society of Chemistry), ref (384) (Copyright 2021 Elsevier), ref (383) (Copyright 2015 American Chemical Society), and ref (390) (Copyright 2019 Elsevier). (c, d) V_6_O_13_ nanosheet obtained by hydrothermal and microwave-assisted synthesis. Reproduced with permission from ref (386) (Copyright 2021 Elsevier) and ref (389) (Copyright 2022 Elsevier).

The mixed-valence V_6_O_13_ attracts extensive attention because it can be used in a variety of ion batteries, such as Li^+^, Na^+^, Mg^2+^, Zn^2+^, and so on. As shown in Figure 46a, V_6_O_13_ is composed of alternating single and double vanadium oxide layers. There are two types of VO_6_ octahedra: V^4+^ occupied the yellow octahedra, and V^5+^ occupied the blue octahedra. All the octahedra are connected with corner O atoms, which form a tunnel-like structure.^49,382^ The solvothermal reaction is widely used to obtain V_6_O_13_ nanostructures, particles, and related composites, such as V_6_O_13_ nanogrooves,^383^ V_6_O_13_@hollow carbon microspheres,^384^ nest-like V_6_O_13_,^385^ V_6_O_13_ nanosheets,^386^ V_6_O_13_ nanowires,^387^ V_6_O_13_ nanorods,^388^ and so on (Figure 46b). Cao et al.^389^ recently developed a new strategy to synthesize V_6_O_13_ nanosheets by microwaves, which could reduce the thickness of V_6_O_13_ nanosheets greatly compared to that prepared by a hydrothermal method (Figure 46c,d).

**Figure 47:**
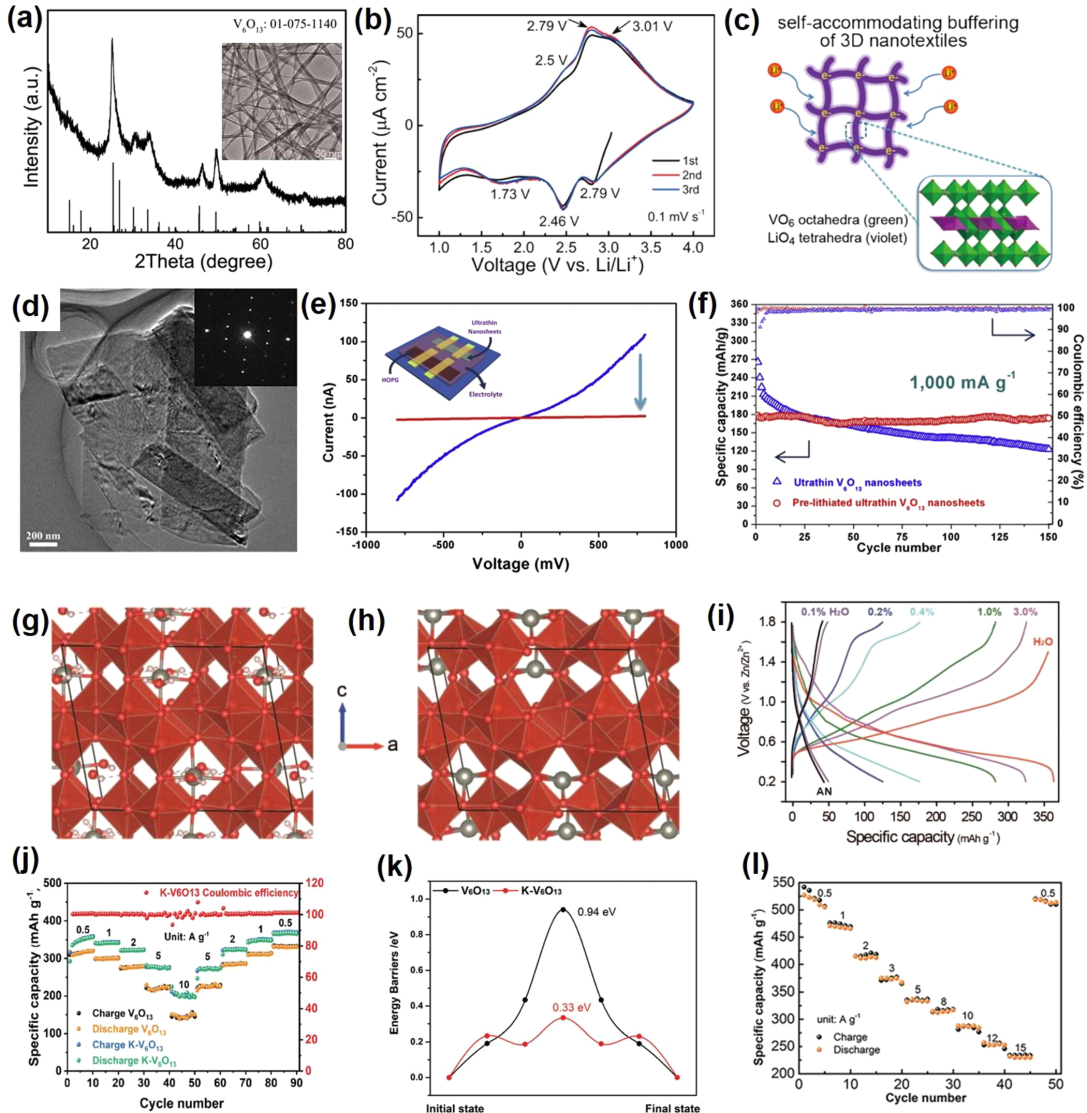
XRD pattern and SEM image (a), different CV curves (b), and the schematic diagram of the Li^+^ intercalation process (c) of 3D V_6_O_13_ nanotextile electrodes in LIB. Reproduced with permission from ref (383). Copyright 2015 American Chemical Society. The TEM image of ultrathin lithiated V_6_O_13_ nanosheets (d), the transport properties (e), and cycling performance at 1000 mA g^–1^ (f) of nonlithiated ultrathin and ultrathin lithiated V_6_O_13_ nanosheets. Reproduced with permission from ref (391). Copyright 2014 Elsevier. Optimized geometry of Zn intercalated V_6_O_13_ with water (g) and without water (h), the galvanostatic voltage-capacity profiles for V_6_O_13_ cycled in electrolytes with different water contents in 1 M Zn(CF_3_SO_3_)_2_ acetonitrile (i). Reproduced with permission from ref (392). Copyright 2019 John Wiley & Sons. (j) Charge/discharge curves of K–V_6_O_13_ at different rates. (k) The migration energy barriers of K–V_6_O_13_ and V_6_O_13_. Reproduced with permission from ref (394). Copyright 2022 Royal Society of Chemistry. (l) Rate capability under various currents of V_6_O_13_ on carbon cloth. Reproduced with permission from ref (385). Copyright 2020 Royal Society of Chemistry.

Due to the tunnel-like structure and mixed-valence, V_6_O_13_ exhibits a metallic character at room temperature, which is beneficial for high-rate charge and discharge. When it is used as an LIB electrode material, 8 mol Li^+^ intercalated per formula unit endowing a high theoretical specific capacity of 417 mAh g^–1^ and energy density of 900 Wh kg^–1^. The Yu group^383^ synthesized a 3D V_6_O_13_ nanotextile with interconnected 1D nanogrooves via a facile solution-redox-based self-assembly route at room temperature (Figure 47a). They confirmed that the precursor concentration affected the mesh size in the textile structure. The 3D V_6_O_13_ delivers a high capacity of 326 mAh g^–1^ at 20 mA g^–1^ and maintains 80% capacity after 100 cycles at 500 mA g^–1^. The energy density can reach 780 Wh kg^–1^, which is much higher than those of commercialization cathodes (LiFePO_4_ and LiCoO_2_) (Figure 47b). The excellent electrochemical performance is due to the unique structure of 3D textiles, which can be maintained upon cycling and are beneficial for ion transport and cycle stability (Figure 47c). In addition, Mai et al.^391^ synthesized a novel ultrathin prelithiated V_6_O_13_ nanosheet by a secondary hydrothermal prelithiation process (Figure 47d). A single-nanosheet device was employed to *in situ* probe the intrinsic advantages of prelithiated nanosheets. Compared with nonlithiated V_6_O_13_ nanosheets, the ultrathin prelithiated V_6_O_13_ nanosheets exhibit a higher electrical conductivity and maintain the same conductance level after the Li^+^ intercalation (Figure 47e). Meanwhile, the specific capacity of the ultrathin prelithiated V_6_O_13_ nanosheets can be maintained at 98% after 150 cycles at a current density of 1000 mA g^–1^, which is much higher than 46% capacity of nonlithiated V_6_O_13_ nanosheets (Figure 47f). These results demonstrate that prelithiation is a strategy to obtain high-energy and long-cycling energy storage cathode materials.

Doping of various metal ions can improve the electrochemical performance and achieve a good capacity of the battery. The V_6_O_13_ with Al/Ga, Al/Fe, and Al/Na doping delivered an initial discharge specific capacity of 411.5 mA h g^–1^, 426.9 mA h g^–1^, and 514 mA h g^–1^ at 0.1 C, respectively.^388^ However, the electrochemical performances were poor at a high current density. V_6_O_13_ was also employed in multivalent ion (Zn^2+^, Mg^2+^) batteries.^384,385,389,392,393^ Choi et al.^392^ applied V_6_O_13_ as the ZIB cathode material and investigated its electrochemical behavior for Zn^2+^. In particular, they analyzed the effect of water in the electrolyte on the Zn^2+^ storage to investigate the physicochemical characteristics of V_6_O_13_. DFT calculation results demonstrate that the coordination environments of Zn show a big difference with/without water (Figure 47g,h). It will form octahedral coordination with water, but undercoordination without water. The Zn^2+^ storage of V_6_O_13_ increases with increasing water content in the electrolyte, and it can deliver a high capacity of 360 mAh g^–1^ and be maintained at 92% after 2000 cycles in an aqueous Zn(CF_3_SO_3_)_2_ (∼1 M) electrolyte (Figure 47i). Even at a high current density of 24 A g^–1^, it maintains a relatively high capacity of 145 mAh g^–1^.^16^ This work highlights that cointercalating water molecules play a vital role in enhancing the electrochemical performance of the aqueous ion storage system. Zhao et al.^394^ found that the K^+^ intercalated V_6_O_13_ exhibited a specific capacity of 367 mAh g^–1^ at 0.5 A g^–1^ and 198.8 mAh g^–1^ at 10 A g^–1^ (Figure 47j). Meanwhile, the capacity could be maintained at 90% after 2000 cycles at 10 A g^–1^. The DFT calculation suggested that K^+^ intercalation could significantly contribute to the reduction of the Zn^2+^ diffusion energy barrier (from 0.94 to 0.33 eV), which enables Zn ions to migrate away from the intercalation sites more easily (Figure 47k). Thus, K^+^ intercalated V_6_O_13_ showed good battery performance. Furthermore, by growing the V_6_O_13_ on carbon cloth and using the ZnSO_4_ as the electrolyte, the V_6_O_13_ cathode showed a capacity of 520 mAh g^–1^ (at a current density of 0.5 A g^–1^) and good cycle life (a stable capacity of 335 mAh g^–1^ over 1000 cycles) (Figure 47l).^385^

### 8.4 Other Vanadium Oxides for Energy-Related Application

**Figure 48:**
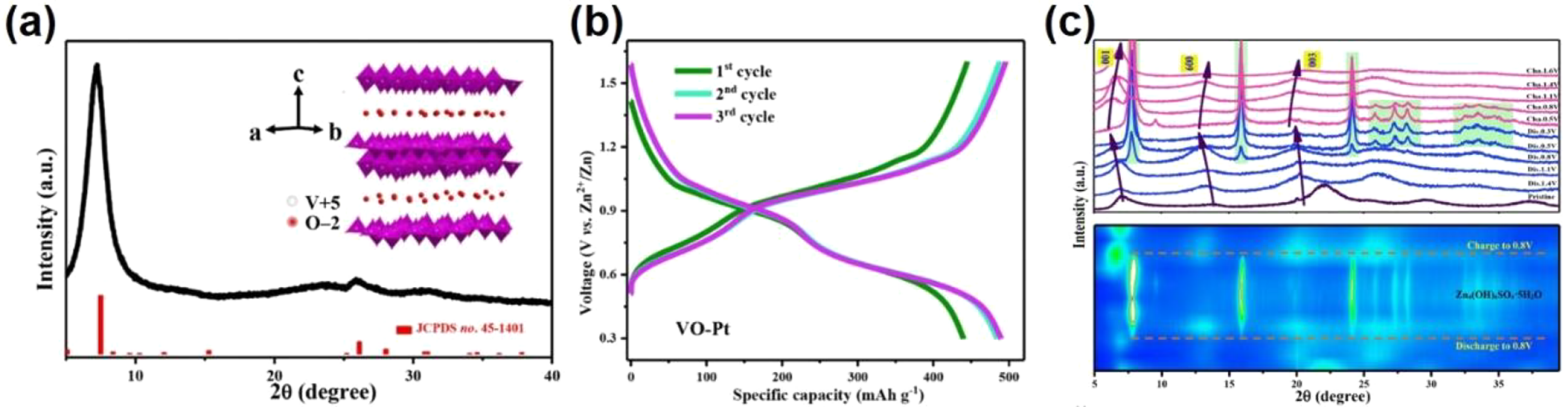
XRD pattern (a), charge/discharge profiles (b), and *in situ* XRD patterns and 2D contour map of peak intensities of V_5_O_12_·6H_2_O–Pt (c). Reproduced with permission from ref (395). Copyright 2021 John Wiley & Sons.

V_5_O_12_·6H_2_O is another layered monoclinic vanadium oxide separated by water pillars with a large interlayer spacing of 1.179 nm. Wang et al.^395^ added a small amount of platinum (Pt, 1.5 wt %) into the interlayer of V_5_O_12_·6H_2_O. The obtained V_5_O_12_·6H_2_O–Pt electrode delivers a specific capacity of 440 mAh g^–1^ at 500 mA g^–1^, and increases to 489 mAh g^–1^ for the third cycle, much higher than V_5_O_12_·6H_2_O electrodes (270 mAh g^–1^ at 500 mA g^–1^) (Figure 48a,b). They also demonstrated that the Pt additive makes no contribution, and is even counterproductive to conductivity, but facilitates a significant enhancement of pseudocapacitance. Therefore, it is clear that there is a strong relationship between Pt and the new phase Zn_4_SO_4_(OH)_6_·5H_2_O. The *in situ* XRD patterns show that obvious characteristic peaks shifted to lower angles during the discharge process and returned to the pristine state during charging, indicating the interlayer spacing gradual enlargement and recovery upon Zn^2+^ intercalation/deintercalation. Meanwhile, the formation/disappearance of the zinc hydroxyl complex is accompanied by Zn^2+^ insertion/extraction (Figure 48c).

**Figure 49:**
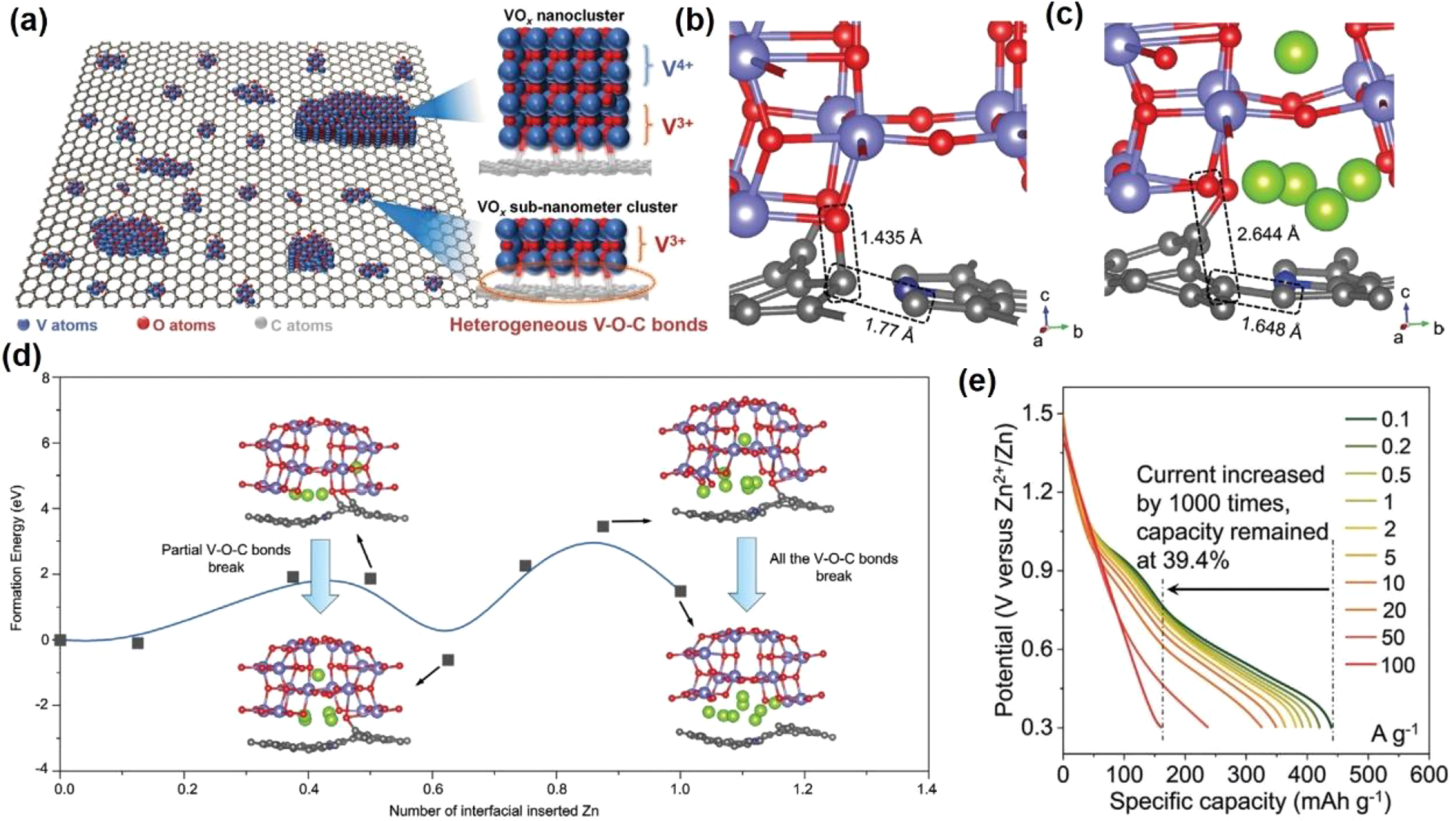
Characterization of the interfacial configuration in the VO_*x*_-G heterostructure and its anomalous Zn^2+^ storage mechanism (a), DFT simulations in pristine (b) and fully discharged (c) states, formation energy evolution during Zn^2+^ ion insertion into the interface (d), galvanostatic discharge curves varying from 0.1 to 100 A g^–1^ (e) of VO_*x*_-G heterostructure. Reproduced with permission from ref (396). Copyright 2021 John Wiley & Sons.

Mai et al.^396^ artificially constructed a VO_*x*_ cluster/reduced graphene oxide (rGO) cathode material with interfacially inserted Zn^2+^ repelling the pristine-bonded C atom into the plane of the rGO and constructing interfacial V–O–C bonds (Figure 49a). Meanwhile, the VO_*x*_ consists of subnanoclusters (less than 1 nm per dimension) and partial nanoclusters (slightly larger than 1 nm). In combination with the electrons transferred to the rGO during the discharge process (Figure 49b–d), the reduced degree of defect is additional proof of the interfacial Zn^2+^ storage. As a result, they have discovered a new mechanism in which Zn^2+^ ions are stored mainly at the interface between VO_*x*_ and rGO, which leads to anomalous valence changes compared to conventional mechanisms and exploits the storage capacity of the nonenergy storing active but highly conductive rGO. The obtained VO_*x*_-G heterostructure delivers a superior rate performance with a capacity of 174.4 mAh g^–1^ at an ultrahigh current density of 100 A g^–1^, with capacity retention of 39.4% for a 1000-fold increase in current density (Figure 49e). To the best of our knowledge, the rate performance is one of the best among ZIBs.

**Figure 50:**
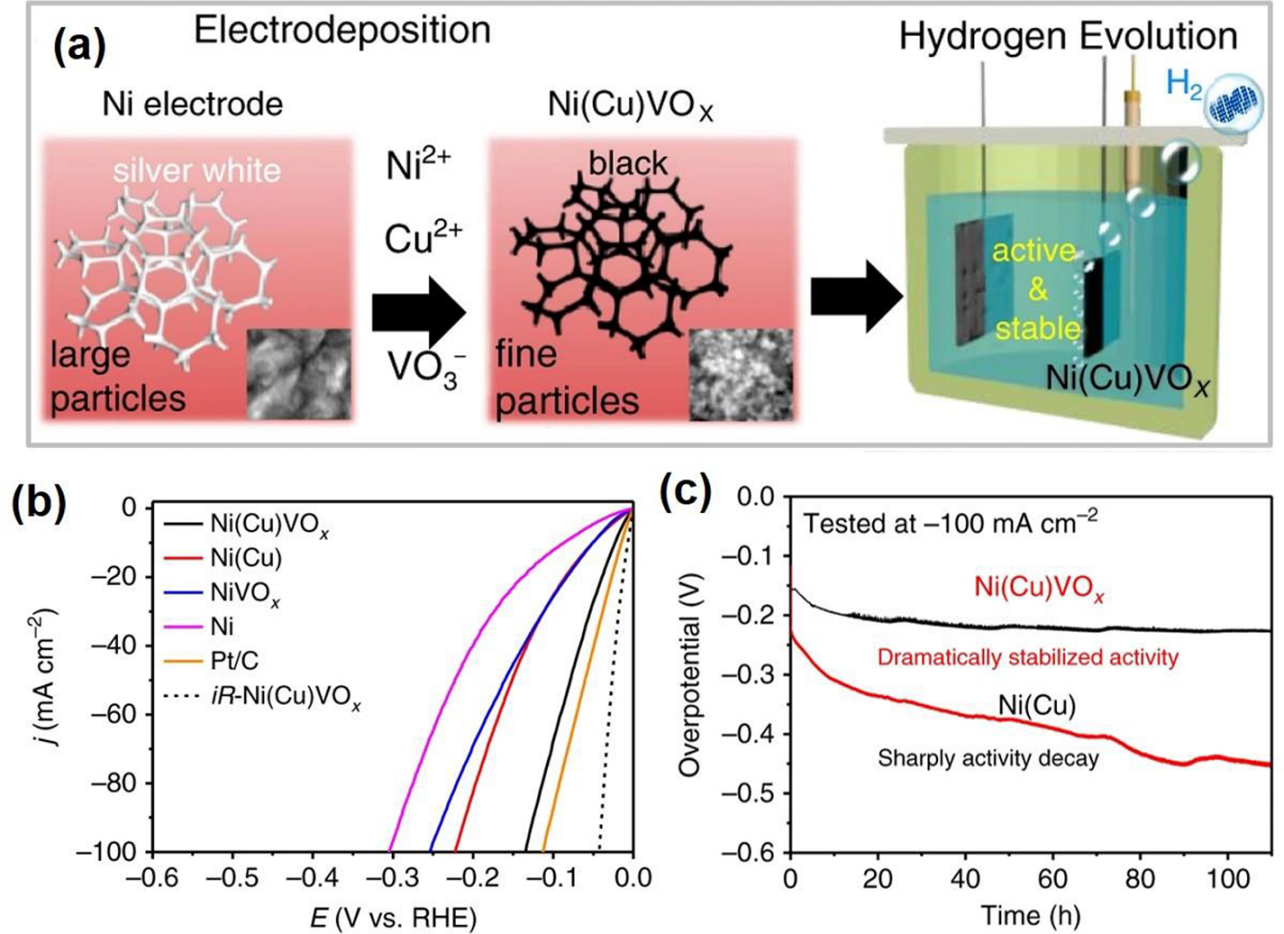
(a) Schematic to prepare the Ni(Cu)VO_*x*_ electrode for HER electrolysis. (b) LSV curves for Ni(Cu)VO_*x*_ and control samples without *iR* compensation. The black dotted curve is the HER activity on the Ni(Cu)VO_*x*_ electrode with *iR* correction. (c) Chronopotentiometric curve of Ni(Cu)VO_*x*_ in comparison with Ni(Cu). Reproduced with permission from ref (397). Copyright 2020 under CC BY license.

Besides the battery application, the nanostructured VO_*x*_ materials/composites have been considered as a good catalyst for the HER due to the creation of the more active site and modification of the adsorption and desorption of H atoms.^397^ Guan et al.^398^ prepared 2D Zn-VO_*x*_-Co ultrathin nanosheets on carbon fiber paper by an electrodeposition method. The Zn-VO_*x*_-Co electrocatalysts contain the amorphous Co metal phase and crystalline Zn–Co alloy phase, which gives the materials a good HER performance (an overpotential of 46 mV at 10 mA cm^–2^ and a Tafel slope of 75 mV dec^–1^). Furthermore, Zhao et al.^397^ adopted the same method to fabricate a Ni(Cu)VO_*x*_ catalyst by changing Zn and Co to Ni and Cu (Figure 50a). The Ni(Cu)VO_*x*_ electrode displays a small overpotential of 21 mV at a current density of 10 mA cm^–2^ and a Tafel slope of 28 mV dec^–1^ (Figure 50b), which is comparable to the commercial 20% Pt/C catalyst (15 mV @ j = 10 mA cm^–2^ and 25 mV dec^–1^). Meanwhile, the Ni(Cu)VO_*x*_ electrode also remains relatively stable for more than 100 h HER at 100 mA cm^–2^ (Figure 50c). The good HER performance is due to the existence of Ni–O–VO_*x*_ sites, which promote the formation of highly disordered metallic Ni structures and further induce electron transfer from Ni to VO_*x*_.

The VO_*x*_ based composites also exhibit good OER performance. Dong et al.^399^ synthesized 3D nanoflower-like VO_*x*_ nanosheets (VO_*x*_/NiS/NF) by a hydrothermal method, which showed the OER performance with a low overpotential (330 mV at 50 mA cm^–2^) and a small Tafel slope (121 mV dec^–1^). Yang et al.^400^ synthesized a ternary Co-VO_*x*_-P catalyst with a nanoflower structure directly onto the Ni foam. The as-prepared materials demonstrated excellent catalytic performance for both the HER (an overpotential of 98 mV at 10 mA cm^–2^ and a Tafel slope of 59 mV dec^–1^) and OER (an overpotential of 230 mV at 100 mA cm^–2^ and a Tafel slope of 64 mV dec^–1^) under an alkaline environment.

## 9 Conclusions and Future Outlook

In the past two decades, there has been accelerated development of vanadium oxides due to the fact that they are the most promising candidates in versatile applications, such as batteries, energy saving smart windows, sensing, catalysts, optoelectronic devices, etc. In this review, we have discussed the V–O binary phase diagram, the structure and synthesis methods of five thermodynamically stable vanadium oxides (V_2_O_3_, V_3_O_5_, VO_2_, V_3_O_7_, V_2_O_5_) and some metastable vanadium oxides (V_2_O_2_, V_4_O_9_, V_6_O_13_) with selected applications on hydrogen evolution catalysis, supercapacitors, batteries, smart windows, and some other aspects. The battery, supercapacitor, and HER/OER performances of these vanadium oxides are summarized for comparison to provide an overview of the research in the field (Tables 1, 2, and 3).

**Table 1:** Electrochemical Properties of the Typical Vanadium Oxides for Metal-Ion Batteries

materials	battery types	Ccapacity (mAh g^–1^)/current density (A g^–1^)	retention (%)/Ccycle numbers/current density (A g^–1^)	rate capacity (mAh g^–1^)/current density (A g^–1^)	ref
VO_*x*_–rGO heterostructure	ZIBs	443/0.1	∼92%/1000/20	174.4/100	(396)
V_2_O_3_ hollow spheres	LIBs	785/0.1	Over 100%/700/2	361/2	(76)
V_2_O_3_ porous nanofibers	KIBs	240/0.05	94.5%/500/0.05	134/1	(68)
V_2_O_3_	ZIBs	625/0.1	97.7%/1000/5	486/20	(77)
V_2_O_3_ with CNTs	SIBs	612/0.1	100%/6000/2	207/10	(361)
			70%/10000/10		
Carbon-confined V_2_O_3_	ZIBs	633.1/0.2	90.3%/10000/12	271.4/24	(401)
V_3_O_5_	LIBs	412/0.2	78.5%/2000/50	125/50	(98)
Graphene quantum dots coated onto the VO_2_ surfaces	LIBs	421/0.1	94%/1500/18	151/36	(237)
Graphene quantum dots coated onto the VO_2_ surfaces	NIBs	306/0.1	88%/1500/18	93/36	(237)
VO_2_ (B) nanorods	KIBs	209.2/0.05	86%/500/0.5	141.4/2	(238)
VO_2_ (B) nanorods	MIBs	391/0.025	41.9%/60/0.85	341/0.1	(402)
VO_2_ hollow microspheres	LIBs	203/0.1	80%/1000/2	134/2	(240)
VO_2_ interwoven nanowires coated with Carbon quantum dots	LIBs	427/0.1	112%/500/19.2	168/19.2	(403)
VO_2_ with graphene ribbons	LIBs	415/0.4	93%/1000/37.2	204/37.2	(404)
VO_2_ (B) nanobelt forest	LIBs	475/0.1	63%/47/0.1	100/27	(405)
VO_2_ nanorods	ZIBs	325.6/0.05	86%/5000/3	72/5	(239)
VO_2_ nanofibers	ZIBs	357/0.1	N.A.	171/51.2	(406)
V_3_O_7_ nanowire templated graphene scrolls	LIBs	321/0.1	87.3%/400/2	162/3	(120)
V_3_O_7_·H_2_O	ZIBs	370/0.375	80%/200/3	270/3	(121)
H_2_V_3_O_8_	MIBs	231/0.01	77%/100/0.04	97/0.08	(108)
H_2_V_3_O_8_ nanowires	ZIBs	423.8/0.1	94.3%/1000/5	113.9/5	(407)
H_2_V_3_O_8_ nanowires with graphene	ZIBs	394/0.1	87%/2000/6	270/6	(408)
V_4_O_7_	LIBs	291/0.05	∼100%/100/3	159/3	(409)
V_4_O_9_	ZIBs	420/0.235	78.8%/1000/9.4	233.4/23.5	(375)
3D V_6_O_13_ nanotextiles	LIBs	326/0.02	80%/100/0.5	134/0.5	(383)
V_6_O_13_ nanosheet	LIBs	331/0.1	98%/150/1	150/2	(391)
V_6_O_13_	ZIBs	360/0.2	92%/2000/4	145/24	(392)
V_6_O_13_ microflowers	SIBs	159.8/0.16	73.5%/30/0.16	N.A.	(410)
Oxygen-deficient V_6_O_13_	ZIBs	401/0.2	95%/200/0.2	223/5	(411)
V_5_O_12_·6H_2_O	ZIBs	440/0.5	∼95%/400/10	158/15	(395)
V_5_O_12_·6H_2_O nanobelt	ZIBs	354.8/0.5	∼94%/1000/2	228/5	(412)
V_10_O_24_·12H_2_O	ZIBs	164.5/0.2	90.1%/3000/10	80/10	(413)
V_10_O_24_·12H_2_O	ZIBs	365.3/0.2	83.2%/3000/5	127.2/80	(414)
V_7_O_16_ nanotube	ZIBs	314.6/0.1	80.5%/950/2.4	87.8/9.6	(415)
V_2_O_5_ hollow microclew	LIBs	145.3/0.1	94.4%/50/0.1	94.8/10	(308)
V_2_O_5_ hollow nanosphere	SIBs	159.3/0.04	72.6%/100/0.16	112.4/0.64	(416)
V_2_O_5_ nanowires	AIBs	305/0.125	89.5%/20/0.125	N.A.	(417)
V_2_O_5_	ZIBs	470/0.2	91.1%/4000/5	386/10	(418)
V_2_O_5_ nanosheet	ZIBs	224/0.1	81.3%/30/0.1	N.A.	(419)
V_2_O_5_ nanofibers	ZIBs	319/0.02	81%/500/0.6	104/3	(376)
Porous V_2_O_5_	LIBs	142/0.075	∼90%/100/0.075	86.7/8.2	(420)
V_2_O_5_/C	SIBs	255/0.015	95%/30/0.015	170/0.294	(309)
V_2_O_5_-polyaniline superlattice	MIBs	270/0.1	61.5%/500/4	130/4	(421)
Polyaniline intercalated V_2_O_5_	NIBs	307/0.5	42%/100/5	69/20	(422)
V_2_O_5_·*n*H_2_O	SIBs	338/0.05	73%/50/0.5	96/1	(423)
V_2_O_5_·*n*H_2_O	ZIBs	372/0.3	71%/900/6	248/30	(310)
V_2_O_5_·*n*H_2_O	CIBs	204/0.14	86%/350/0.7	28/2.8	(424)
3D V_2_O_5_/RGO/CNT	LIBs	304/0.0294	90%/80/0.294	100/5.88	(311)
Na_0.33_V_2_O_5_	ZIBs	367.1/0.1	93%/1000/1	96.4/2	(312)
Mn_0.01_V_1.99_O_5_	LIBs	251/0.294	80%/50/0.294	171/1.47	(313)
UGF-V_2_O_5_/PEDOT	LIBs	297/0.294	98%/1000/17.64	85/23.52	(314)

**Table 2:** Electrochemical Properties of the Typical Vanadium Oxides for Supercapacitors

materials	electrolyte	Specific capacitance (F g^–1^)/Current density (A g^–1^)	Retention (%)/Cycle numbers	ref
VOOH nanosheets	1 M LiClO_4_/PPC	323/0.2	70%/2000	(425)
V_2_O_3_ nanoflakes@C core–shell composites	1 M NaNO_3_	205/0.05	76%/500	(94)
V_2_O_3_/C nanocomposites	5 M LiCl	458.6/0.5	86%/1000	(93)
V_2_O_3_@C core–shell nanorods	5 M LiCl	228/0.5	81%/1000	(426)
V_2_O_3_@C core–shell nanorods	1 M Na_2_SO_4_	192/1	66%/1000	(427)
V_2_O_3_@C core–shell composites	1 M Na_2_SO_4_	223/0.1	39.7%/100	(428)
N-doped carbon coated nest-like V_3_O_7_	1 M Na_2_SO_4_	660.63/0.5	80.47%/4000	(112)
		187.72/50		
V_3_O_7_ nanowires on carbon fiber cloth	1 M Na_2_SO_4_	198/1	97%/100000	(125)
V_3_O_7_·H_2_O nanobelts/CNT/rGO composites	5 M LiCl/PVA	685/0.5	99.7%/10000	(126)
V_3_O_7_-rGO-polyaniline composites	1 M H_2_SO_4_	579/0.2	95%/2500	(429)
VO_2_(B) nanobelts/rGO composites	0.5 M K_2_SO_4_	353/1	78%/10000	(235)
VO_2_ nanosheet	1 M LiClO_4_/PPC	405/1	82%/6000	(430)
VO_2_ nanosheet	6 M KOH	663/10	99.4%/9000	(431)
VO_2_(B)/C core–shell composites	1 M Na_2_SO_4_	203/0.2	10.4%/100	(432)
Graphene foam/VO_2_ nanoflakes/hydrogen molybdenum bronze composites	1 M K_2_SO_4_	485/2	97.5%/5000	(433)
		306/32		
VO_2_ microarrays	1 M Na_2_SO_4_	265/1	100%/3000	(434)
VO_2_@polyaniline coaxial nanobelts	0.5 M Na_2_SO_4_	246/0.5	28.6%/1000	(435)
V_4_O_9_ nanosheets	1.5 M KOH	392/0.5	75%/2000	(373)
V_6_O_13_@C	1 M Na_2_SO_4_	545/0.5	88.3%/2000	(436)
V_6_O_13_ sheets	1 M NaNO_3_	285/0.05	96.7%/300	(437)
V_6_O_13_	1 M LiNO_3_	456/0.6	65%/2000	(438)
Sulfur-doped V_6_O_13–x_@C	5 M LiCl	1353/1.9	92.3%/10000	(439)
RuO_2_ nanoparticle decorated V_2_O_5_ nanoflakes	1 M KCl	421/1	94.6%/10000	(315)
Graphene nanoribbons @ V_2_O_5_ nanostrips	0.5 M Na_2_SO_4_	335.8/1	∼98.5%/10000	(316)
N-doped carbon nanofibers/V_2_O_5_ core/shell	1 M Na_2_SO_4_	595.1/0.5	97%/12000	(317)
Interconnected V_2_O_5_ Nanoporous Network	0.5 M K_2_SO_4_	304/0.1	24%/600	(440)
V_2_O_5_/rGO nanocomposites	8 M LiCl	537/1	84%/1000	(441)
V_2_O_5_/rGO hybrids	1 M Na_2_SO_4_	468.5/1	91.5%/10000	(442)
V_2_O_5_/graphene hybrid aerogels	1 M Na_2_SO_4_	486/0.5	90%/20000	(273)
V_2_O_5_/rGO composite hydrogel	0.5 M Na_2_SO_4_	320/1	70%/1000	(443)
Carbon coated V_2_O_5_ nanorods	0.5 M K_2_SO_4_	417/0.5	76%/1000	(444)
		341/10		
Hollow spherical V_2_O_5_	5 M LiNO_3_	559/3	70%/100	(445)
V_2_O_5_ nanorods	1 M LiClO_4_	347/1	94.3%/10000	(446)
V_2_O_5_ nanorods/rGO	0.75 M NaPF_6_	289/0.01	85%/1000	(447)
V_2_O_5_ nanosheets/rGO	1 M KCl	635/1	94%/3000	(448)
V_2_O_5_ nanobelts	1 M LiClO_4_/ PPC	132.5/1	95%/500	(449)
V_2_O_5_ nanobelts/rGO	0.5 M K_2_SO_4_	310.1/1	90.2%/5000	(319)
V_2_O_5_ microtubules	1 M LiNO_3_	680/1	70%/10000	(271)
V_2_O_5_ nanoparticles	1 M LiClO_4_/ PPC	545/1	70%/500	(450)
V_2_O_5_/graphene hybrid	1 M Na_2_SO_4_	484/0.6	80%/10000	(451)
V_2_O_5_/graphene hybrid aerogel composite	1 M LiClO_4_/ PPC	384/0.1	82.2%/10000	(452)
		197/2		
V_2_O_5_/MWCNT core/shell hybrid aerogels	1 M Na_2_SO_4_	625/0.5	120%/20000	(453)
Carbon coated flowery V_2_O_5_	1 M K_2_SO_4_	417/0.5	100%/2000	(454)
V_2_O_5_/mesoporous carbon microspheres	1 M Al_2_(SO_4_)_3_	290/0.5	88%/10000	(455)
Graphene-wrapped V_2_O_5_ nanospheres	1 M Na_2_SO_4_	612.5/1	89.6%/10000	(456)
V_2_O_5_/polypyrrole	5 M LiNO_3_	448/0.8	81%/1000	(457)
V_2_O_5_/polyaniline	0.5 M LiClO_4_/ PPC	1115/1	90%/4000	(458)
V_2_O_5_@Ni_3_S_2_	1 M KOH	854/1	60%/1000	(330)
V_2_O_5_/Na_0.33_V_2_O_5_	1 M LiClO_4_	334/1	96%/1000	(459)
V_2_O_5_/TiO_2_	1 M LiNO_3_	587/0.5	92%/5000	(460)
V_2_O_5_ nanowire arrays/N-doped graphene aerogel	8 M LiCl	710/0.5	95%/20000	(461)
V_2_O_5_ nanofibers/conductive polymer	1 M Na_2_SO_4_	614/0.5	111%/15000	(462)
V_2_O_5_/nanoporous carbon network	0.5 M K_2_SO_4_	314.6/0.2	89.5%/5000	(463)
V_2_O_5_/Ni foam	1 M KOH	399.7/0.01	96.1%/2000	(464)
V_2_O_5_/WO_3_	1 M H_2_SO_4_	386/0.1	104%/5000	(465)
V_2_O_5_/g-C_3_N_4_	1 M KOH	192.3/0.5	85.7%/5000	(466)
V_2_O_5_@Ti	1 M LiCl	1520/1.5	99%/12000	(467)
V_2_O_5_/vertically aligned CNT	1 M Na_2_SO_4_	284/2	76%/5000	(468)
V_2_O_5_ nanosheets/carbon fiber felt	5 M LiCl	475.5/1	89.7%/6000	(469)

**Table 3:** A Brief Survey of Typical Vanadium Oxides Electrocatalysts

catalyst	catalyst type	electrolyte	η @ *j* = 10 mA cm^–2^ (mV)	Tafel slope (mV dec^–1^)	stability (cycle number or time @ current density)	ref
V_2_O_3_/ MoS_*x*_ /CC	HER	0.5 M H_2_SO_4_	146	45	1000 cycles	(82)
NiFe@V_2_O_3_	HER	1 M KOH	84	85	24 h @ 10 mA cm^–2^	(80)
NiFe@V_2_O_3_	OER	1 M KOH	255	51	1000 cycles	(80)
					24 h @ 10 mA cm^–2^	
Ni_0.8_/V_2_O_3_	HER	1 M KOH	44	38	24 h @ 10 mA cm^–2^	(470)
V_2_O_3_–Ni_3_N	HER	1 M KOH	57	50	24 h @ 10 mA cm^–2^	(471)
V_2_O_3_@Ni	HER	1 M KOH	47	74	1000 cycles	(472)
					10 h @ 10 mA cm^–2^	
V_2_O_3_–CoFe_2_O_4_	HER	1 M KOH	61	58	80 h @ 500 mA cm^–2^	(473)
V_2_O_3_–CoFe_2_O_4_	OER	1 M KOH	226	56	80 h @ 500 mA cm^–2^	(473)
Ni_4_Mo–V_2_O_3_	HER	1 M PBS	40	66	1000 cycles	(474)
					5.5 h @ 50 mA cm^–2^	
MoS_2_/VO_2_	HER	0.5 M H_2_SO_4_	99 @ 1 mA cm^–2^	85	N. A.	(245)
Ni_3_S_2_/VO_2_	HER	1 M KOH	100	114	15 h @ 10 mA cm^–2^	(244)
Ni_3_S_2_/VO_2_	OER	1 M KOH	150	47	15 h @ 10 mA cm^–2^	(244)
Co_3_O_4_/VO_2_/CC	HER	1 M KOH	108	98	10 h @ 10 mA cm^–2^	(246)
porous VO_2_ nanosheets	HER	0.5 M H_2_SO_4_	184	70	120 h @ 90 mA cm^–2^	(247)
porous VO_2_ nanosheets	OER	1 M KOH	209	92	120 h @ 70 mA cm^–2^	(247)
CoV_2_O_6_ - V_2_O_5_/NRGO	OER	1 M KOH	239	50	1000 cycles	(320)
Co–V_2_O_5_	HER	1 M KOH	51	42	24 h @ 10 mA cm^–2^	(328)
V_2_O_5_/Ni(OH)_2_@NF	HER	1 M KOH	39	44	10000 cycles	(329)
Pt (P)–V_2_O_5_/graphene	HER	0.5 M H_2_SO_4_	32	23	1000 cycles	(331)
V_2_O_5_@Ni_3_S_2_	HER	1 M KOH	95	108	9000 cycles	(330)
Ni–Co–P/V_2_O_5_–TiO_2_/GO	HER	1 M KOH	101 @ 100 mA cm^–2^	36	N. A.	(475)
Zn-VO_*x*_-Co	HER	1 M KOH	46	75	36 h @ 100 mA cm^–2^	(398)
Ni(Cu)VO_*x*_	HER	1 M KOH	21	28	125 h @ 100 mA cm^–2^	(397)
VO_*x*_/NiS/NF	OER	1 M KOH	330 @ 50 mA cm^–2^	121	1000 cycles	(399)
Co-VO_*x*_-P	HER	1 M KOH	98	59	1000 cycles	(400)
					24 h @ 30 mA cm^–2^	
Co-VO_*x*_-P	OER	1 M KOH	230 @ 100 mA cm^–2^	64	1000 cycles	(400)
					25 h @ 100 mA cm^–2^	
VO_*x*_@NiFe/NiCoP/TM	HER	1 M KOH	45	34	1000 cycles	(476)
					16 h @ 100 mA cm^–2^	
VO_*x*_@NiFe/NiCoP/TM	OER	1 M KOH	215	37	20 h @ 500 mA cm^–2^	(476)
Co(VO_*x*_)	HER	1 M KOH	178 @ 100 mA cm^–2^	40	60 h @ 100 mA cm^–2^	(477)

**Figure 51:**
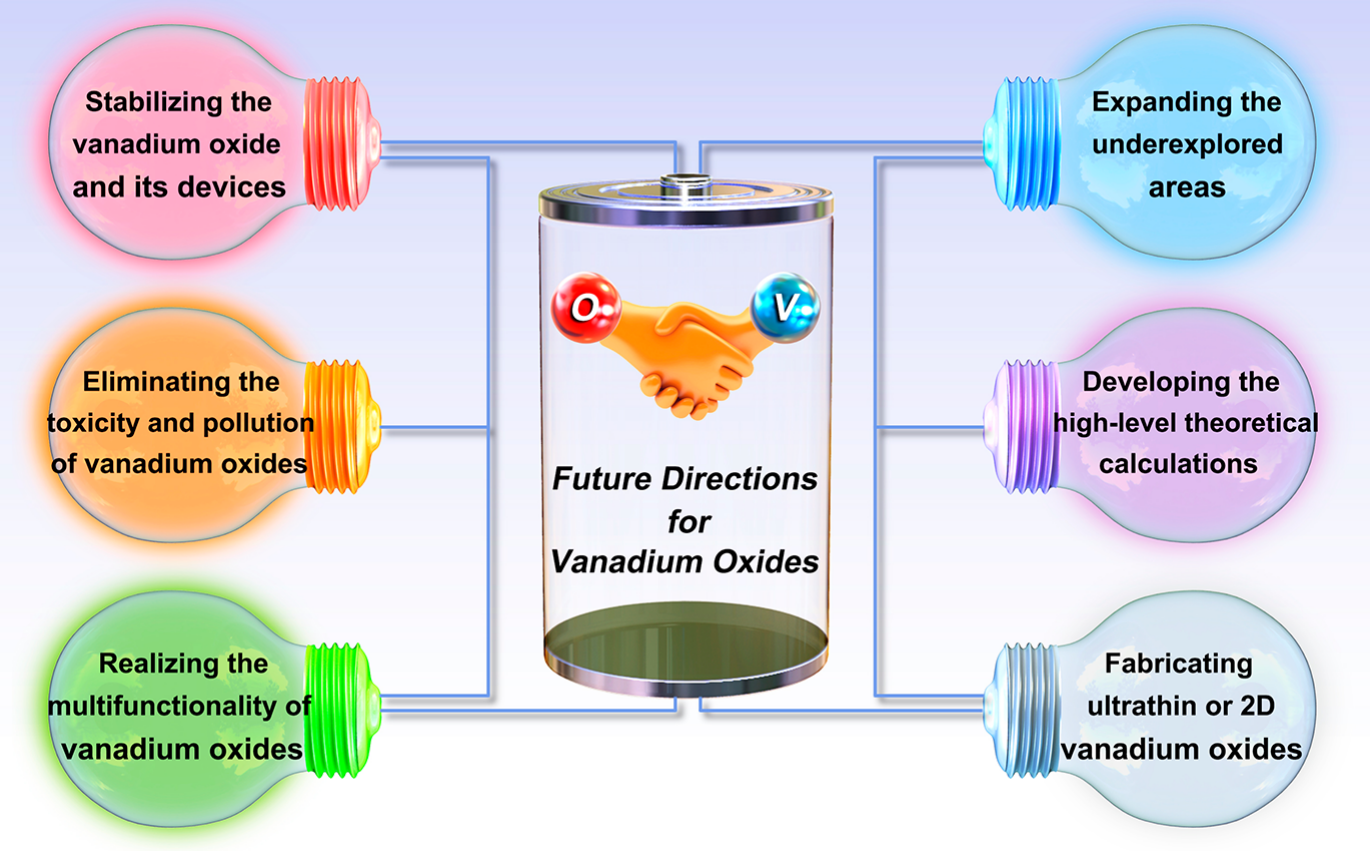
Future development and research directions for vanadium oxides.

While vanadium oxides are important materials for many applications, more detailed, mechanistic and systematical studies are needed to fully explore their potential as the bottleneck is increasingly related to the materials’ quality and device fabrications. We propose a few future works in this field which could be further developed in the following aspects (Figure 51):

(1) Searching the applications of vanadium oxides in underexplored areas. One example is biological thermal imaging by leveraging its thermal phase transition characteristics as the insulator-to-metal phase transition of some vanadium oxides gives sharply enhanced optical absorption above their critical temperature. It has potential applications in biochemistry, especially in the fields of bioimaging. Another example is that some nanostructured vanadium oxides also possess optimal physicochemical properties (e.g., optical, thermal, magnetic properties), which give the opportunity to be applied in intelligent medicine based on micro-/nanorobots.
(2) Development of high-level theoretical calculations to guide the rational design of vanadium oxides and their composites and to better understand the fundamentals of high-performance devices. The use of advanced computational tools and theories enables researchers to understand the origin of complex and interacting phenomena at multiple scales, which could accelerate our understanding of the fundamentals of high-performance devices and improve the operation and design of new materials systems. Additionally, machine learning is a useful toolkit for designing and exploring new vanadium oxides with desired properties. It can also aid the understanding of the complex correlations between structures and properties in vanadium oxides.
(3) Fabrication of ultrathin or two-dimensional vanadium oxides, which could be applied in some new electronic devices. Owing to the atomic-scale thickness of single layers, the 2D materials exhibit tunable electrical properties and bandgaps. Therefore, the 2D vanadium oxides may hold promise for a wide range of applications in low-power electronics, flexible electronics, optoelectronics, catalysis, batteries, and so on. Furthermore, the 2D vanadium oxides may also form novel 2D heterostructures with other ultrathin 2D nanomaterials, which would be of certain interest.
(4) Exploration of new approaches to stabilize the vanadium oxides related devices, especially thermal, light, moisture, and oxygen environmental stability. Long-term device stability is one of the most important challenges for all devices. Most vanadium oxides are sensitive to oxygen and moisture, which may lead to the degradation of the device performances. Furthermore, most stability measurements of the devices are performed under ambient conditions, which limits their application in high temperature, high humidity, and high light intensity conditions. Meanwhile, it is also important to understand the degradation mechanisms in the different types of devices based on vanadium oxides, which could be the key to improving the device stabilities.
(5) Exploring new green chemistry synthesis methods to eliminate the toxicity of vanadium oxides. Vanadium oxides cause a variety of toxic effects such as biochemical changes, neurobehavioral injury, and functional lesions in the liver and bones. Especially, vanadium oxides in breathing air can cause pulmonary problems and DNA damage in leukocytes. The toxicity is more related to the phase structure, stoichiometric ratio, concentration, particle size, and crystalline degree, which could be considered in all the processes of application of vanadium oxides. Therefore, exploring new green synthesis methods and avoiding risks to humans and the environment during vanadium oxides’ production, use, and disposal processes deserve more systematic and comprehensive studies. First, it is important to develop new synthesis protocols with minimum steps to prepare vanadium oxides, such as one-pot synthesis, completely enclosed-system synthesis, and so on. Second, the encapsulation of the devices to confine the toxicity needs to be considered when designing a new device. Third, the release of vanadium oxides to the atmosphere should be controlled during normal operation. For example, the marked decrease in toxicity is confirmed via silica coating on vanadium oxides due to the perception that the toxicity of vanadium oxide is closely related to the solubility and the robust silica barrier can isolate air and water to reduce the solubility.
(6) Realization of the bifunctional, trifunctional, or even multifunctional vanadium oxides to achieve integrated functionality. Different device integrations based on the same materials is expected to greatly reduce the cost and the incompatibility of different materials. Meanwhile, multifunctional materials would reduce the complexity of designing devices and promote the application in designated situations.

Overall, we believe that vanadium oxides are great candidates for future applications in related fields and help solve key challenges in the global warming crisis. Moreover, the integration of vanadium oxides with multidisciplinary fields such as material science, device physics, civil engineering, mechanical design, and bioscience would continue to attract the interest of many scientists from different disciplines for new fascinating fields.

## Abbreviations

1D: one-dimensional
2D: two-dimensional
3D: three-dimensional
4D: four-dimensional
AACVD: aerosol-assisted chemical vapor deposition
AC: activated carbon
AFM: atomic force microscope
AIB: aluminum-ion battery
ALD: atomic layer deposition
AM 1.5: air mass 1.5
BP: black paint
CC: carbon cloth
*C*_dl_: double-layer capacitance
CIB: calcium-ion battery
CNC: carbon nanocoils
CNT: carbon nanotube
CV: cyclic voltammetry
CVD: chemical vapor deposition
DC: direct current
DFT: density functional theory
DNA: DNA
EBD: electron-beam deposition
EN: ethylenediamine
GCD: galvanostatic charge/discharge
GF: graphene foam
GITT: galvanostatic intermittent titration technique
GQD: graphene quantum dot
GVG: graphene foam supported graphene quantum dot anchored VO_2_ arrays
FeFET: ferroelectric field-effect transistor
FET: field-effect transistor
GO: graphene oxide
HCP: high concentration dilution
HER: hydrogen evolution reaction
High-E: high emissivity
HM: hollow microclew
IL: ionic liquid
IR: infrared
ITO: indium tin oxide
KIB: potassium-ion battery
LCP: low concentration dilution
LED: light emitting diode
LIB: lithium-ion battery
LiTFSI: lithium bis-trifluoromethanesulfonimide
Low-E: low emissivity
LWIR: longwave infrared
LSPR: localized surface plasmonic resonance
MAS NMR: magic-angle-spinning nuclear magnetic resonance
MB: methylene blue
MCE: magnetocaloric effect
MD: molecular dynamics
MEMS: microelectromechanical systems
MIB: magnesium-ion battery
MIT: metal–insulator transition
MO: methyl orange
MWCNT: multiwalled carbon nanotube
N.A.: not applicable
NaTFSI: sodium bis-trifluoromethanesulfonimide
NDR: negative differential resistance
NF: Ni foam
NIB: ammonium-ion battery
NIR: near-infrared
NMR: nuclear magnetic resonance
NP: nanoparticle
NRGO: nitrogen-doped reduced graphene oxide
NW: nanowire
OER: oxygen evolution reaction
PBS: phosphate-buffered saline
PDMS: polydimethylsiloxane
PEDOT: poly(3,4-ethylenedioxythiophene)
PEG: polyethylene glycol
PLD: pulsed laser deposition
PNCNF: porous N-doped carbon nanofiber
PPC: propylene carbonate
PS: polystyrene
PVA: poly(vinyl alcohol)
PVC: polyvinyl chloride
PVD: physical vapor deposition
PVDF-HFP: polyvinylidene fluoride-hexafluoropropylene
PVP: polyvinylpyrrolidone
PZT: Pb(Zr_0.52_Ti_0.48_)O_3_
RC: radiative cooling
RCRT: radiative cooling regulating thermochromic
ReRAM: resistive random-access memory
rGO: reduced graphene oxide
RhB: rhodamine B
RF: radio frequency
RT: room temperature
SA-VO_2_: surface amorphized VO_2_
SC: supercapacitor
SCE: saturated calomel electrode
SCVA: single-crystalline VO_2_ actuator
SEM: scanning electron microscope
SHE: standard hydrogen electrode
SIB: sodium-ion battery
SWCNT: single- or multiwalled carbon nanotube
TARC: temperature-adaptive radiative coating
TEM: transmission electron microscope
TGA: thermogravimetric analysis
TM: titanium mesh
UGF: ultrathin graphite foam
UHV: ultrahigh vacuum
UV–vis–NIR: ultraviolet–visible-near-infrared
VOG: V_2_O_5_·H_2_O/graphene
VGS: V_3_O_7_ nanowire templated graphene scroll
V_2_O_3_@NC: nitrogen-doped carbon-confined V_2_O_3_
XANES: X-ray absorption near edge structure
XPS: X-ray photoelectron spectroscopy
XRD: X-ray diffraction
ZIB: zinc-ion battery
